# ASXL gain-of-function truncation mutants: defective and dysregulated forms of a natural ribosomal frameshifting product?

**DOI:** 10.1186/s13062-017-0195-0

**Published:** 2017-10-16

**Authors:** Adam M. Dinan, John F. Atkins, Andrew E. Firth

**Affiliations:** 1 Department of Pathology, Division of Virology, University of Cambridge, Cambridge, CB2 1QP UK; 2 School of Biochemistry and Cell Biology, University College Cork, T12 YT57, Cork, Ireland; 3 Department of Human Genetics, University of Utah, Salt Lake City, UT 84112 USA

**Keywords:** Ribosomal frameshifting, Translation, Protein synthesis, ASXL1, ASXL2, HCF-1, BAP1, Additional sex combs-like, Overlapping gene

## Abstract

**Background:**

Programmed ribosomal frameshifting (PRF) is a gene expression mechanism which enables the translation of two N-terminally coincident, C-terminally distinct protein products from a single mRNA. Many viruses utilize PRF to control or regulate gene expression, but very few phylogenetically conserved examples are known in vertebrate genes. Additional sex combs-like (*ASXL*) genes 1 and 2 encode important epigenetic and transcriptional regulatory proteins that control the expression of homeotic genes during key developmental stages. Here we describe an ~150-codon overlapping ORF (termed *TF*) in *ASXL1* and *ASXL2* that, with few exceptions, is conserved throughout vertebrates.

**Results:**

Conservation of the *TF* ORF, strong suppression of synonymous site variation in the overlap region, and the completely conserved presence of an EH[N/S]Y motif (a known binding site for Host Cell Factor-1, HCF-1, an epigenetic regulatory factor), all indicate that *TF* is a protein-coding sequence. A highly conserved UCC_UUU_CGU sequence (identical to the known site of +1 ribosomal frameshifting for influenza virus PA-X expression) occurs at the 5′ end of the region of enhanced synonymous site conservation in *ASXL1*. Similarly, a highly conserved RG_GUC_UCU sequence (identical to a known site of −2 ribosomal frameshifting for arterivirus nsp2TF expression) occurs at the 5′ end of the region of enhanced synonymous site conservation in *ASXL2*.

**Conclusions:**

Due to a lack of appropriate splice forms, or initiation sites, the most plausible mechanism for translation of the ASXL1 and 2 *TF* regions is ribosomal frameshifting, resulting in a transframe fusion of the N-terminal half of ASXL1 or 2 to the *TF* product, termed ASXL-TF. Truncation or frameshift mutants of ASXL are linked to myeloid malignancies and genetic diseases, such as Bohring-Opitz syndrome, likely at least in part as a result of gain-of-function or dominant-negative effects. Our hypothesis now indicates that these disease-associated mutant forms represent overexpressed defective versions of ASXL-TF.

**Reviewers:**

This article was reviewed by Laurence Hurst and Eugene Koonin.

**Electronic supplementary material:**

The online version of this article (10.1186/s13062-017-0195-0) contains supplementary material, which is available to authorized users.

## Background

The shifting of ribosomes between reading frames whilst decoding mRNA transcripts can give rise to protein isoforms with distinct C-terminal sequences, and often quite different lengths, from the equivalent products of standard decoding [1]. Specific features of the mRNA sequence can evolve to increase or modulate the frequency with which frameshifting occurs and, in such cases, the process may be referred to as programmed ribosomal frameshifting (PRF) [2]. PRF is commonly utilized in virus gene expression, where it serves to control the ratio of different enzymatic or structural proteins, or to allow access to overlapping open reading frames (ORFs) thus increasing the coding capacity of small virus genomes [3].

In viruses, the most common type of PRF involves −1 tandem slippage of the P- and A-site tRNAs on a “slippery” heptanucleotide sequence with consensus motif X_XXY_YYZ (where XXX represents any three identical nucleotides, although certain exceptions occur – such as GGU and GUU; YYY represents AAA or UUU; Z represents A, C, or U; and underscores seperate zero-frame codons). Such sequences allow for substantial codon:anticodon re-pairing following a −1 PRF. For efficient −1 PRF, however, an extra stimulatory element is required and this normally takes the form of a downstream RNA stem-loop or pseudoknot structure separated from the heptanucleotide shift site by a 5–9 nt “spacer” region. Such RNA-structure-stimulated frameshifting normally results in a fixed ratio of frameshift to non-frameshift protein products. In two cases, PRF is known to be stimulated by virus proteins binding to the mRNA downstream of the shift site and, in such cases, the frameshifting efficiency can be modulated as levels of virus protein increase over the course of infection [4, 5].

Ribosomes can access the other available reading frame via either +1 or −2 PRF. The *cis*-acting sequences directing “plus direction” frameshifting events have generally been less well-defined than those associated with −1 PRF [6]. However, a number of specific instances have been documented. For example, ~1–2% efficient +1 PRF occurs at a highly-conserved UCC_UUU_CGU sequence during translation of the influenza A virus PA gene, giving rise to the transframe protein PA-X [6, 7]. The precise mechanistic basis for +1 PRF in this case remains unclear; however, it has been proposed to involve P-site slippage of the single isoacceptor phenylalanine tRNA (anticodon 3′-AAG-5′) on the UUU_C tetranucleotide. The identities of the A-site (CGU) and E-site (UCC) codons also affect the PRF efficiency, but there appears to be no strong dependence on more distal sequence elements [7]. Similar sequences are conserved and have been proposed as the sites of +1 frameshifting in chronic bee paralysis and related viruses, amalgaviruses, and fijiviruses [6, 8].

In arteriviruses, ~20%-efficient −2 PRF occurs at a slippery sequence in the nsp2-encoding region of the *pp1a* gene [4, 9, 10]. In this case, the shift site is normally RG_GUU_UUU or RG_GUC_UCU (R = purine), depending on virus species, allowing codon:anticodon re-pairing in the A-site following a −2 shift. Unlike canonical “minus direction” frameshifting, −2 PRF in arteriviruses does not appear to require the presence of a downstream stimulatory RNA secondary structure, but instead involves the binding of a protein complex – comprising the viral nsp1β protein and cellular Poly(C)-Binding Proteins (PCBPs) – to a downstream C-rich sequence separated from the shift site by a “spacer” region of 10 nt [4].

**Fig. 1:**
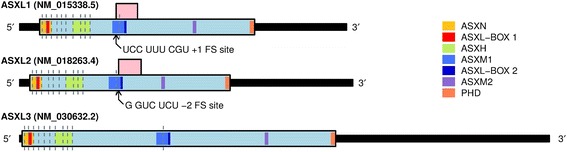
Architecture of human *ASXL* mRNA transcripts. Each transcript comprises 13 exons, with exon 13 being the longest. The zero-frame coding regions (1541, 1435 and 2248 codons respectively) for each transcript are shown in light blue. Exon boundaries are denoted by vertical dashed lines; exon 3 (3 nt) is not visible in the figure. The locations of conserved domains are indicated, according to the colour legend. The conserved +1 and −2 PRF shift sites are shown for *ASXL1* (UCC_UUU_CGU) and *ASXL2* (G_GUC_UCU). Ribosomes which frameshift would translate a conserved +1 frame ORF (pink). ASXN: ASX N-terminal domain; ASXH: ASX homology domain; ASXM: ASX middle domain; PHD: plant homeodomain

There are very few known cases of biologically relevant PRF in vertebrate cellular genes. The gene for antizyme – a negative regulator of cellular polyamine levels – utilizes +1 PRF to regulate synthesis of antizyme as part of a feedback loop where the efficiency of frameshifting increases in response to elevated polyamine levels [11, 12]. As in many retrotransposons, the retrotransposon-derived genes *PEG10*, *PNMA3*, *PNMA5* and *ZCCHC5* contain canonical −1 PRF sites [13, 14]. To our knowledge, no other conserved (i.e. subject to purifying selection) cases of PRF are known in vertebrate nuclear-encoded genes [1]. The limited informational content of +1 PRF sites makes their computational identification challenging. However, it is most feasible in cases where the sequence of the frameshift site is phylogenetically conserved; and where ribosomes which shift frame do not immediately encounter a stop codon, but rather continue to synthesize a so-called “transframe” protein product. During a routine scan of vertebrate genomes, we identified a single highly conserved instance of the influenza A virus UCC_UUU_CGU +1 shift site. This is located in a central region of the *ASXL1* coding sequence, and coincides with the conserved presence of a long +1 frame overlapping ORF (hereafter TF, from “transframe”) and statistically significantly enhanced synonymous site conservation in the zero-frame. A corresponding +1 frame overlapping ORF is also present in *ASXL2*, but here the ORF is associated with a highly conserved arterivirus RG_GUC_UCU −2 shift site (Fig. 1).

*ASXL* genes encode regulatory proteins of the enhancer of trithorax and polycomb (ETP) group, which modulate the expression of homeotic genes during embryogenesis [15, 16]. There are three paralogous members of the *ASXL* family in vertebrates (*ASXL1–3*) [17], and a single homologous gene (*ASX*) in *Drosophila* [18]. ASXL proteins function as epigenetic scaffolds, capable of interacting with chromatin modification complexes, and can also independently bind to a number of transcription factors [16]. Despite their varying lengths, the *ASXL* genes share a conserved architecture; with each gene comprising a total of 13 exons and 12 introns (Fig. 1). Exon 13 is by far the longest in each case, accounting for almost three-quarters of full-length *ASXL* mRNA transcripts, and including the entire 3′ untranslated region (UTR) [17, 19]. The locations of the splice junctions at intron-exon boundaries have been confirmed experimentally, and their sequences are highly conserved [17].

A number of domains are found in ASXL proteins, the relative locations of which are broadly conserved (Fig. 1). Encoded at the extreme N terminus of each protein is the ASXN domain (also known as the HB1, ASXL, restriction endonuclease helix-turn-helix or HARE-HTH domain), which is predicted to facilitate interactions with DNA [20]. Downstream of ASXN is the ASX homology (ASXH) domain (also known as the DEUBAD domain) encoded by exons 9–11, which participates in interactions with epigenetic regulatory proteins, including the BRCA1 Associated Protein 1 (BAP1) deubiquitinating protease [21–23]. The predicted PRF sites in *ASXL1* and *ASXL2* occur within the regions encoding the non-globular ASXM1 domain and the binding site of the transcriptional co-regulator SRC-1 (NCOA1) [24, 25]. Frameshifting at the predicted sites would give rise to truncated forms of the ASXL proteins, ASXL1-TF and ASXL2-TF, lacking the C-terminal ASXM2 and plant homeodomain (PHD) domains, which appear to function primarily in binding to nuclear hormone receptors [25], and to histone proteins [26], respectively, but acquiring a conserved EH[N/S]Y motif close to the C-terminus of TF.

## Results

### A conserved overlapping ORF in a central region of mammalian *ASXL1* and *ASXL2*

Following the identification of UCC_UUU_CGU as the site of +1 PRF in influenza A virus [6], we screened 37,257 human mRNA RefSeq CDSs from the National Center for Biotechnology Information (NCBI) database for in-frame UCC_UUU_CGU sequences. We found 12 unique matches (i.e. excluding the same match occurring in different transcript isoforms) of which one – in *ASXL1* – was conserved in mouse, chimpanzee, cow and chicken. The two paralogous members of the *ASXL* family (*ASXL2* and *ASXL3*) were also inspected. Neither paralogue was found to contain an influenzavirus-like +1 PRF sequence; however, *ASXL2* was found to contain an arterivirus-like −2 PRF shift site sequence, RG_GUC_UCU, at a location similar to that of the *ASXL1* +1 PRF sequence, and conserved in the same species. In human, the *ASXL1* and *ASXL2* shift site sequences are followed by +1-frame ORFs of 153 and 161 codons, respectively. Frameshift translation of the overlapping ORFs would result in transframe fusion proteins of 77 and 89 kDa (ASXL1-TF and ASXL2-TF) compared to 165 and 154 kDa for the full-length zero-frame products (ASXL1 and ASXL2) (Additional file 1: Figure S1).

### Conservation of the frameshift site and overlapping ORF

Following initial identification in a limited number of sequences, we next expanded the datasets for both *ASXL1* and *ASXL2* to include mRNA sequences from each of the major clades of vertebrates. Specifically, we queried the NCBI non-redundant nucleotide (nr/nt) and transcriptome shotgun assembly (TSA) databases to detect orthologues of both genes, resulting in the identification of sequences from 200 species for *ASXL1* and 129 species for *ASXL2* (Additional file 1).

**Fig. 2:**
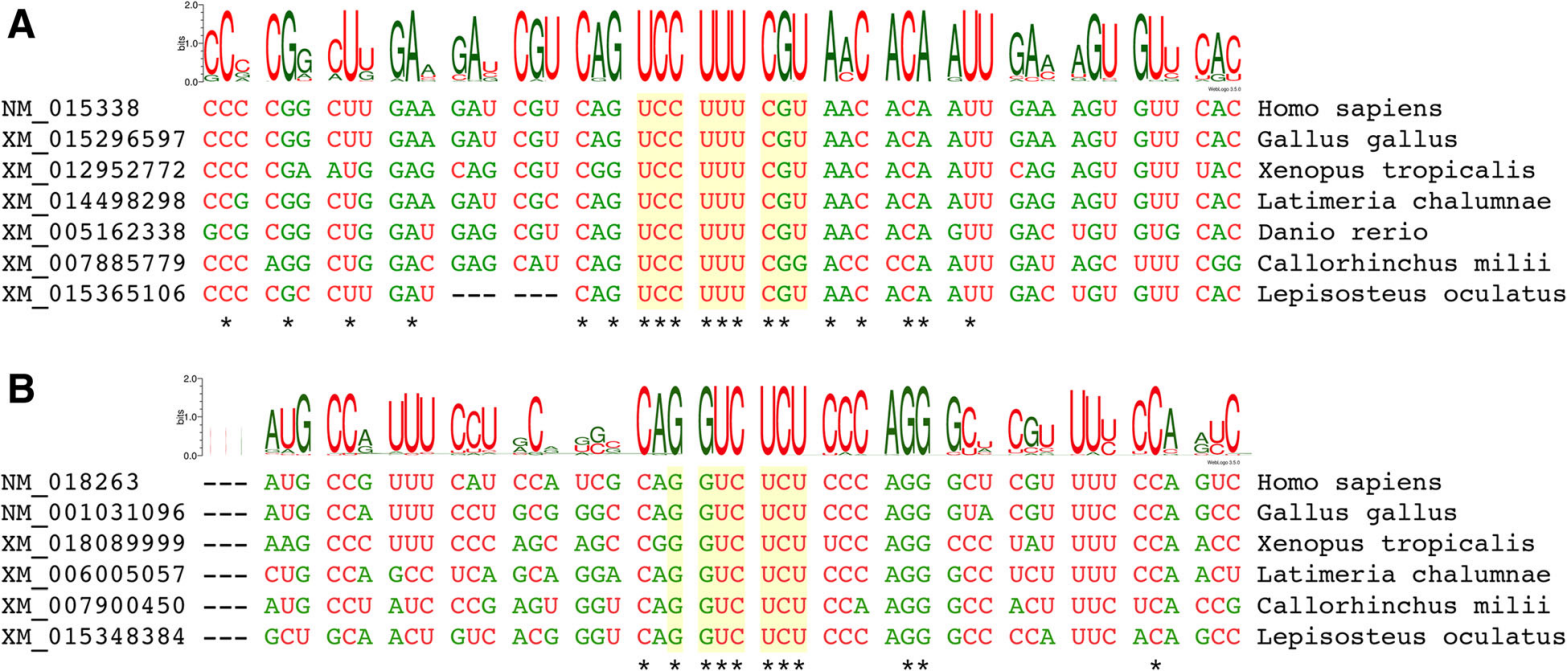
Codon alignments of selected *ASXL* sequences in the vicinity of the predicted frameshift sites. **a** *ASXL1*. **b** *ASXL2*. To avoid over-representation of closely related sequences, sequence logos are based on alignments of 76 (*ASXL1*) and 52 (*ASXL2*) sequences from phylogenetically diverse taxa (see Additional file 1). Selected individual sequences from major vertebrate clades are shown beneath. Asterisks indicate conservation of residues within the full alignments, while dashes indicate insertions or deletions within those alignments. Zero-frame codons are separated by spaces and the predicted frameshift sites are highlighted in yellow

In the case of *ASXL1*, the UCC_UUU_CGU sequence was found to be conserved in each species for which a sequence was identified, with the sole exception of the Australian ghostshark (*Callorhinchus milii*), in which the last nucleotide is a G rather than a U (i.e. UCC_UUU_CGG) (Fig. 2a). Studies with reporter constructs have shown that the presence of a CGG codon at this position, rather than CGU, reduces the efficiency of +1 PRF at the influenza A virus shift site by ~50% [7]. Hence, these data are consistent with the occurrence of PRF in the *ASXL1* gene of the Australian ghostshark, although perhaps at a lower efficiency when compared with other vertebrates. The putative shift site sequence is followed by a +1-frame ORF with median length of 153, 138, 126 and 163 codons in mammals, sauropsids, amphibians and teleost fish, respectively, and 158, 139 and 162 codons in coelacanth (*Latimeria chalumnae*), spotted gar (*Lepisosteus oculatus*) and Australian ghostshark (see Additional file 1).

In the case of *ASXL2* (Fig. 2b), the RG_GUC_UCU sequence was found to be conserved in all taxa, apart from lizards (represented by a single species, *Anolis carolinensis*) and teleost fish. The lizard sequence is apparently divergent from those of other reptiles, in which the RG_GUC_UCU sequence is fully conserved. A single teleost *ASXL2* sequence – from the early-branching *Scleropages formosus* [27] – contains the RG_GUC_UCU sequence, but the corresponding +1-frame *TF* ORF is short (20 codons). In contrast, the sequences from spotted gar and Australian ghostshark contain the RG_GUC_UCU sequence and full-length *TF* ORFs. Hence, a parsimonious interpretation of these data is that *ASXL2* in the last common ancestor of bony and cartilaginous fish contained the *TF* ORF but it was secondarily lost within teleosts. The putative shift site sequence is followed by a +1-frame ORF with median length of 161, 156 and 152 codons in mammals, sauropsids and amphibians, respectively, and 155, 153 and 138 codons in coelacanth, spotted gar and Australian ghostshark (see Additional file 1).

**Fig. 3:**
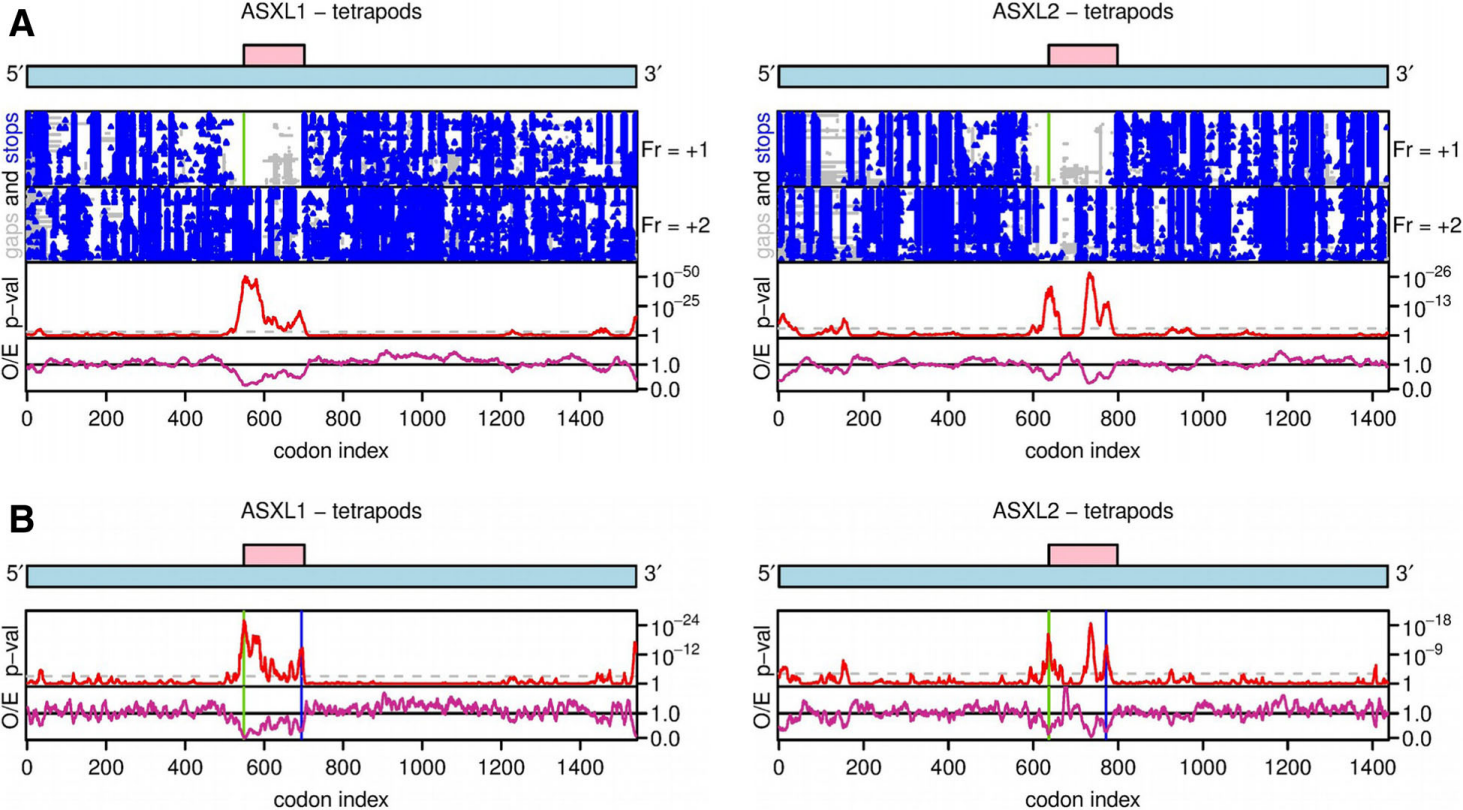
Synonymous site conservation in the *ASXL1* and *ASXL2* coding regions in tetrapods. **a** In each subfigure, the top panel shows a schematic of the zero-frame ORF (pale blue) and the overlapping *TF* ORF (pink). The next two panels show positions of stop codons (blue) in the +1 and +2 reading frames, and alignment gaps (grey) in each sequence of the sequence alignment. The vertical green line in the +1 frame panel shows the position of the putative frameshift site. The bottom two panels show the synonymous site conservation analysis, with the magenta line (lower panel) indicating the ratio of the observed number of substitutions within a given window to the number expected under a null model of neutral evolution at synonymous sites, and the red line (upper panel) showing the corresponding *p*-value. The analysis uses a 25-codon sliding window. The horizontal dashed grey line indicates a *p* = 0.05 threshold after a correction for multiple testing (namely scaling by [25-codon window size]/[*ASXL* CDS length]. **b** As above, but with a 9-codon sliding window. The vertical green and blue lines show the positions of the putative frameshift site and the conserved EH[N/S]Y, respectively. (See Additional file 1: Figs. S2 and S3 for teleost fish besides separate mammalian, sauropsid and amphibian analyses)

Within each well-represented vertebrate clade (i.e. mammals, sauropsids, amphibians and teleost fish), codon-based alignments of the zero-frame *ASXL* coding regions of all identified orthologues were constructed, and synonymous site conservation was assessed as previously described [28]. In each alignment highly significant synonymous site conservation was observed in a region coincident with the +1 frame TF ORF (Fig. 3 and Additional file 1: Figure S2; see Table 1 for *p*-values). The predicted PRF shift sites occur at the 5′ end of the region of conservation and notably often correspond to specific conservation peaks when synonymous site conservation was analyzed at higher resolution (Fig. 3 and Additional file 1: Figure S3). Known dual coding regions (e.g. in viruses) are frequently characterized by significant synonymous site conservation in the overlapping zero-frame, reflecting constraints on sequence evolution imposed by the overlapping feature [28]. Although overlapping non-coding features (such as functional RNA structures) may also lead to enhanced synonymous site conservation, the highly conserved presence of a long open reading frame in two distinct paralogues, i.e. *ASXL1* and *ASXL2*, argues in favour of an overlapping coding sequence.

**Table 1:** Zero-frame observed/expected (obs/exp) synonymous substitution ratios and corresponding *p*-values for the *TF* region

Gene	Taxon	Synonymous site conservation	
		obs/exp	*p*-value
*ASXL1*	mammals	0.33	3.3 × 10^−82^
	sauropsids	0.55	4.4 × 10^−29^
	amphibians	0.43	6.3 × 10^−19^
	tetrapods	0.43	4.8 × 10^−121^
	teleosts	0.52	3.2 × 10^−49^
*ASXL2*	mammals	0.68	7.6 × 10^−22^
	sauropsids	0.86	5.7 × 10^−3^
	amphibians	0.75	6.0 × 10^−4^
	tetrapods	0.74	1.3 × 10^−22^

Note that these *p*-values are for the entire *TF* ORF and therefore can be lower than the 25-codon sliding window *p*-values of Fig. 3 and Additional file 1: Fig. S2

We considered possible explanations for *TF* ORF expression, other than ribosomal frameshifting. In particular, it is conceivable that a previously unrecognized alternative splice variant might fuse the *TF* ORF in-frame with the canonical *ASXL* CDS. Several transcript isoforms have been identified for both *ASXL1* and *ASXL2*, although the majority of these seem to result from the use of alternative poly-adenylation signals in the 3′ UTR [17, 29]. Alternative splice site usage has also been documented. For example, the existence of a second major isoform of *ASXL1* has been experimentally verified in human cells, which results from the utilization of an alternative fourth exon [30]; however, this transcript also does not allow in-frame expression of the *TF* ORF. To systematically search for annotated alternative splice variants (or completely independent mRNAs) that might allow translation of the TF peptide, we queried the NCBI blastp non-redundant protein (nr) database using the translated *TF* ORF sequences of *ASXL1* and *ASXL2* from human. A small number of non-human hits were found in both cases, though all were based upon computational gene predictions and none covered the query sequence completely. Alignment of the zero-frame sequences of *ASXL1* and *ASXL2* with these database hits suggests that the splice junctions have been mis-annotated in the latter, apparently due to missing or incomplete underlying genomic sequence data. For example, the best match to the *ASXL2* TF peptide sequence was a hypothetical protein (A6R68_07077) from *Neotoma lepida* (GenBank accession OBS64385.1). However, the genomic sequence upon which the gene prediction is based (LZPO01097212.1) contains numerous large gaps, which are likely to have affected the prediction of splice junctions, and, in fact, the start codon of the gene has not been annotated. We conclude that there is currently no robust evidence for alternative splicing providing a potential mechanism to access the *ASXL1* or *ASXL2 TF* ORFs.

Independent expression of the *ASXL1* and *ASXL2* TF polypeptides is also unlikely given the lack of appropriately positioned AUG codons within the *TF* ORF sequences (Additional file 1: Figure S4).

### Amino acid composition of the TF peptides

**Fig. 4:**
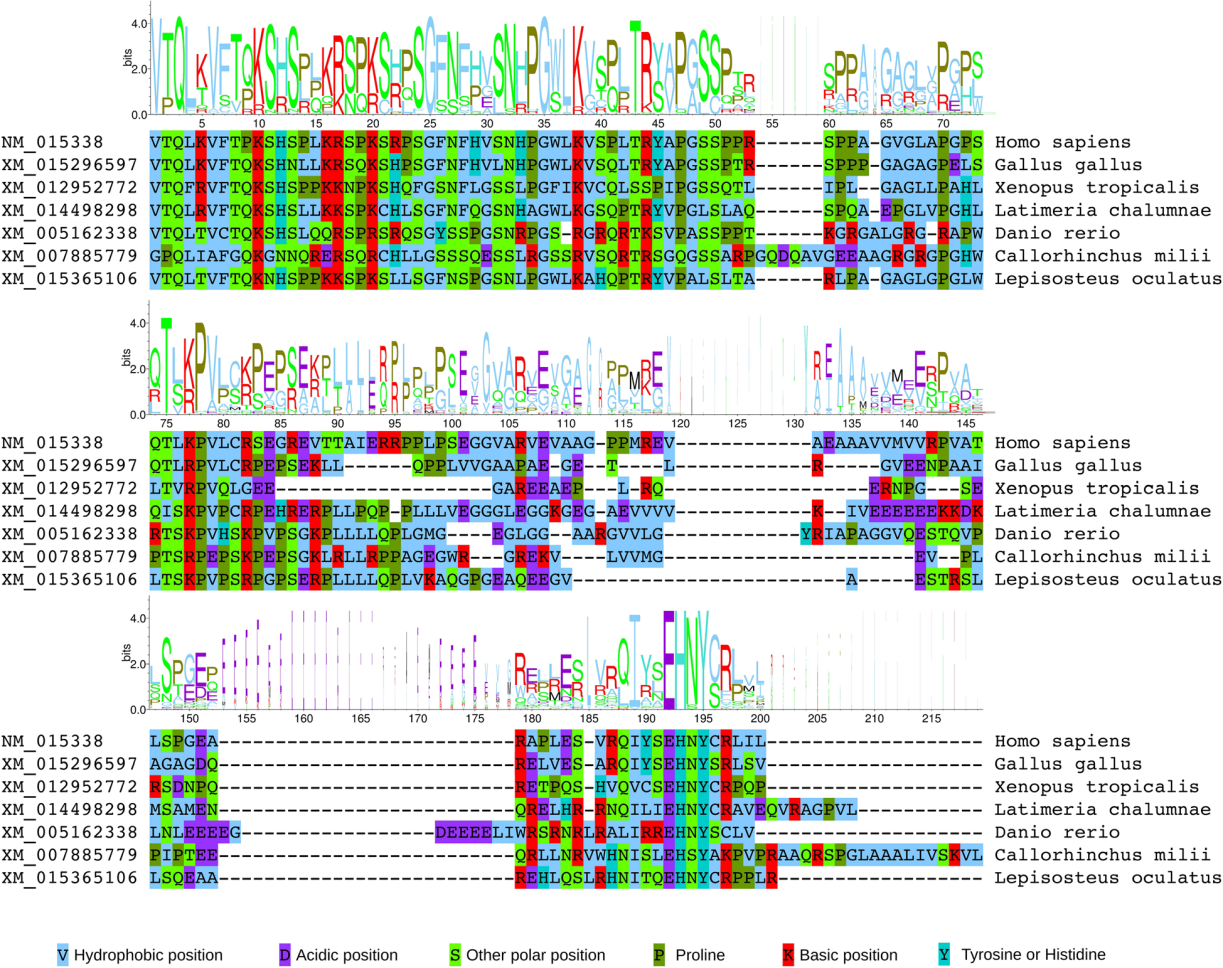
Amino acid composition of *ASXL1* TF peptide sequences. To avoid over-representation of closely related sequences, the sequence logo is based on an alignment of 76 sequences from phylogenetically diverse taxa (see Additional file 1: Figure S2). Selected representative sequences are shown beneath the logo. Partial-width logo letters correspond to positions with alignment gaps (i.e. fewer contributing sequences)

**Fig. 5:**
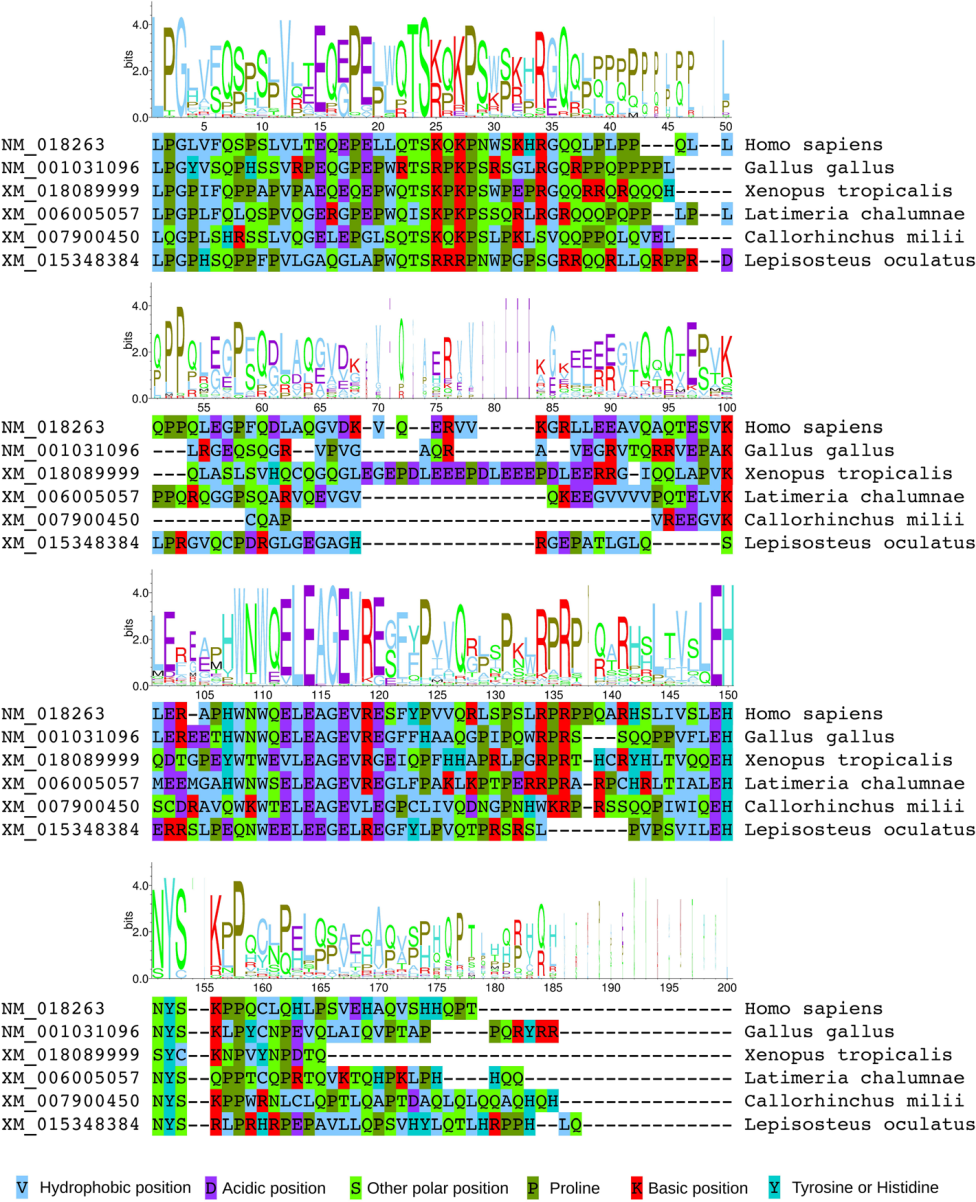
Amino acid composition of *ASXL2* TF peptide sequences. To avoid over-representation of closely related sequences, the sequence logo is based on an alignment of 52 sequences from phylogenetically diverse taxa (see Additional file 1: Figure S2). Selected representative sequences are shown beneath the logo. Partial-width logo letters correspond to positions with alignment gaps (i.e. fewer contributing sequences). Note that teleost fish lack the ASXL2 *TF* ORF

TF peptide sequences were determined by translating the +1 reading frames from the 3′ ends of the predicted PRF shift sites to the nearest in-frame stop codons (see Additional file 1: Figure S1). The TF peptides of phylogenetically representative species from each major vertebrate clade were then aligned, and residue conservation was assessed [31]. For both *ASXL1* (Fig. 4) and *ASXL2* (Fig. 5), conservation of amino acids was observed in the N-terminal region of TF. This relatively high level of conservation could potentially reflect selective constraints on the nucleotide sequence due to potential 3′ PRF-stimulatory elements and/or amino acid coding constraints in the zero-frame-encoded ASXM1 domain (Fig. 1). Nonetheless, it is apparent that amino acids with particular physicochemical properties are maintained at specific sites. For example, there are five positions in the alignment of *ASXL1* TF peptides at which a basic (K/R) amino acid is found in all sequences, four of which are in the N-terminal region (positions 10, 17, 20, and 38 in Fig. 4).

A four amino acid motif, EH[N/S]Y, was found to be common to the C-terminal regions of all TF sequences analysed, both for *ASXL1* and *ASXL2* (Figs. 4 and 5). This motif is commonly followed by a serine (S) or a hydrophobic residue, and – in the case of *ASXL2* only – by a proline (P) residue typically located four amino acids downstream. Additionally, in the case of *ASXL2*, the ~40 amino acid region upstream of the EH[N/S]Y motif is well-conserved; in particular, a distinct LE[A/E]G[E/Q] motif was observed at the beginning of this region (positions 113–117 in Fig. 5).

We searched the eukaryotic linear motif (ELM) database [32] using the predicted human TF peptide sequences to determine whether any of the highly conserved regions might comprise known functional motifs. This analysis revealed that the core EH[N/S]Y sequence matches the metazoan Host Cell Factor-1 (HCF-1) binding motif (HBM), which has the consensus [D/E]HxY [33, 34]. For both *ASXL1* and *ASXL2*, this was the most significant database match for the entire TF peptide sequence (*p* = 5.1 × 10^−5^). The EH[N/S] Y motifs also coincide with distinct peaks in synonymous site conservation (Fig. 3 and Additional file 1: Figure S3) indicating that these sites are subject to particularly strong evolutionary constraints. A zero-frame amino acid sequence corresponding to +1 frame EH[N/S]Y is necessarily highly constrained – for example, the first two positions can only be R/G and T/A respectively; yet all four amino acids were common at these positions, for example human ASXL1 and ASXL2 have zero-frame RTQLL and GAQLQ respectively at this site, confirming that conservation of +1 frame EH[N/S]Y is not due to zero-frame coding constraints.

### Structural analyses of TF peptides

No significant homology was found between the TF peptide sequences and tertiary structural domains within the InterProScan [35] or NCBI conserved domains [36] databases. We used the Predictor of Natural Disordered Regions (PONDR®) algorithm to infer ordered and disordered segments in the full-length ASXL1 and ASXL2 proteins of humans (Additional file 1: Figure S5; left panel, top and bottom, respectively) and in the predicted frameshift products ASXL1-TF and ASXL2-TF (Additional file 1: Figure S5; right panel, top and bottom, respectively). The TF regions were inferred to be largely disordered (Additional file 1: Figure S5; right panel, highlighted sections). However, it is notable that the C-terminal EH[N/S]Y motifs are predicted to be found within locally ordered segments of the transframe products. Combined with the analyses of residue conservation discussed above, these data suggest that the TF peptides do not harbour functional tertiary structures, but they may exert a regulatory impact by means of conserved short linear motifs.

### Analysis of ribosome profiling datasets

Ribosomal frameshifting into the *TF* ORF would result in a proportion of translating ribosomes terminating at the *TF* stop codon, leading to a step-wise decrease in ribosome density at this site that might be apparent in ribosome profiling datasets [5, 37]. Due to variability in read density as a result of library preparation biases (nuclease, ligation, reverse transcription, PCR, etc), ribosome profiling is unlikely to be sensitive enough to detect intragenic drop-off at levels of ~20% or less, let alone the 1–2% frameshifting, previously measured by metabolic labelling, on the UCC_UUU_CGU shift site in the influenza A virus sequence context. Nonetheless, we inspected *ASXL* genes in ribosome profiling datasets on the GWIPS-viz genome browser [37]. Most datasets did not show noticeable drop-off at the end of the *TF* ORF for either *ASXL1* or *ASXL2*, though a few data sets showed moderate to strong drop-off. To more clearly visualize ribosome drop-off, we removed intronic regions by remapping datasets to *ASXL1* and *ASXL2* mRNA transcripts. Two examples – Jurkat cells from Gawron et al. [38] and MDA-MB-231 cells from Rubio et al. [39] – are shown in Additional file 1: Figure S6. For *ASXL1*, the mean ribosome footprint density downstream of the *TF* ORF was 0.15 times the mean density upstream of the *TF* ORF in Jurkat cells, whereas in MDA-MB-231 cells the ratio was 1.01, indicating that 85% of ribosomes drop off the *ASXL1* mRNA within the *TF* region in Jurkat cells but not in MDA-MB-231 cells. For *ASXL2* the ratios were less extreme and much closer to each other – 0.69 and 0.85 in Jurkat and MDA-MB-231 cells respectively – making it harder to distinguish *TF*-specific drop-off from potential generic decreases in ribosome footprint density perhaps due to other causes.

While the Jurkat *ASXL1* ribosome drop-off ostensibly supports efficient PRF in certain cell types, we are suspicious that this particular result is an artefact of heterozygous somatic mutations. Jurkats are a pseudodiploid cell line, with polyploidy occurring in a moderate percentage of cells. Analysis of Gawron et al. RiboSeq reads (typically ~30 nt) mapping to the *ASXL1 TF* region revealed two indel mutations – a GCCCG to GCCCCG insertion present in 29 of 74 reads and a AGGGGGGGGU to AGGGGGGGU deletion present in 5 of 5 reads. The former mimics a −1 frameshift leading to premature termination in the middle of the *TF* ORF. The latter mimics a +1 frameshift leading to termination at the *TF* stop codon. Interestingly, to our knowledge Jurkat cells have not been reported to have indels in *ASXL1* (https://cansar.icr.ac.uk/cansar/cell-lines/JURKAT/), and analysis of genomic sequencing datasets (SRX2596625, SRX2596624; 150-nt reads) also did not reveal indels at these sites. Thus the Gavron et al. indels may be specific to their isolate of the Jurkat cell line. In summary, therefore, current ribosome profiling datasets neither support nor contradict the PRF hypothesis. However, given that the PRF efficiencies involved may be of order a few percent, it is likely that *ASXL* PRF would not be detectable by this approach.

## Discussion

Hitherto, most documented cases of PRF have been found to occur within mobile genetic elements and particularly in the genomes of RNA viruses [1]. Less attention has been paid to frameshifting as a gene expression mechanism in cellular organisms; however, there are a small number of notable instances of both prokaryotic and eukaryotic chromosomal genes whose expression is dependent upon PRF [1]. The efficiency of frameshifting in two such cases (prokaryotic release factor 2, and eukaryotic antizyme) is regulated via feedback loops. For example, the antizyme genes of yeast and metazoa consist of two partially overlapping reading frames (ORF1 and ORF2), and +1 PRF at the last codon of the former is required for the translation of full-length, biochemically active antizyme proteins [40]. Frameshifting at these sites is responsive to cellular polyamine levels, which are, in turn, decreased by the actions of the antizyme proteins; thereby completing an autoregulatory circuit [11, 41]. The antizyme frameshift site consists of a highly conserved UCC_UGA sequence, as well as more divergent flanking sequences, including a specific 5′ sequence and a pseudoknot located 3′ of the shift site [40, 42].

In the case of the putative frameshift sites in the *ASXL1* and *ASXL2* genes, we found no clear evidence for the existence of conserved flanking RNA structures, either pseudoknots or stem loops (see Methods). It is possible that more rapidly evolving, lineage-specific structures might play a role in either or both cases; or that *trans*-acting factors might be involved, as has been shown for −1 PRF in cardioviruses and −2 PRF in arteriviruses [4, 5]. Clearly, experimental data will be required to determine the propensity of ribosomes to transition between reading frames on *ASXL* mRNAs, and to shed light on the efficiency and stimulators. However, given that the expression levels of *ASXL* genes are tightly controlled across distinct tissues and cell types [17], it seems plausible that PRF might also be regulated in these genes.

Frameshifting at the predicted sites is expected to give rise to the truncated forms, ASXL1-TF and ASXL2-TF. Since the *TF* stop codon is located within the last exon, frameshifting – even if highly efficient – is not expected to lead to nonsense-mediated decay of *ASXL* mRNAs [43, 44]. ASXL1-TF and ASXL2-TF retain the N-proximal ASXN and BAP1-binding ASXH domains (Fig. 1), but contain an alternative C-terminal region harbouring a conserved EHXY motif, homologous to the binding site for the transcriptional co-regulator HCF-1 [45]. HCF-1 interacts with transcription factors [34, 46] and with chromatin modulation complexes [47] via its N-terminal kelch repeat beta propeller domain, to control cell proliferation and the expression of homeotic genes [48, 49], in a cell cycle dependent manner [50–52]. Interestingly, although no direct link has been found between HCF-1 and full-length ASXL1 or ASXL2, full-length ASXL1, ASXL2 and HCF-1 have been shown, independently, to be amongst the strongest binding partners of the BAP1 deubiquitinase [50–56]. ASXL proteins interact with BAP1 to form the polycomb repressive deubiquitinase (PR-DUB) complex, which removes monoubiquitin from histone 2A at lysine 119 (H2AK119) [21, 23]. Our data now raise the intriguing possibility that the frameshifted isoforms ASXL1-TF and ASXL2-TF might compete with BAP1 for binding to HCF-1 and thus provide an alternative link between BAP1 and HCF-1 in a reconfigured complex that also lacks the ASXL C-proximal histone-interacting PHD domain.

Nonsense and frameshift-inducing mutations giving rise to truncated isoforms of ASXL1 are among the most frequently observed mutations in myeloid malignancies [24, 57]. Such mutations are usually heterozygous suggesting a gain-of-function or dominant negative effect [44, 58, 59] although loss-of-function has also been proposed [60–62]. Similarly, heterozygous germline truncation of ASXL1 is the underlying cause of Bohring-Opitz syndrome [63], and related but distinct neurodevelopmental anomalies are associated with heterozygous germline truncation of ASXL2 [44]. As the mutant isoforms typically contain the ASXH domain (Fig. 1), they retain the ability to associate with BAP1 [58]. The resulting protein complexes exhibit enhanced H2AK119 deubiquitinating activity, leading to secondary loss of histone H3 lysine 27 trimethylation (H3K27me3) marks and the widespread activation of genomic loci which are otherwise epigenetically silenced [58]. The mutant isoforms typically have a domain structure similar to ASXL-TF except that they generally (but not always) lack the C-terminal EH[N/S]Y motif. Further, the mutant forms will presumably be translated at the normal level of ASXL (though the resulting mutant proteins may be unstable) whereas ASXL-TF should be less efficiently expressed and/or regulated. Thus we predict that disease-associated ASXL truncation mutants represent defective overexpressed versions of a natural functionally important ribosomal frameshifting product ASXL-TF.

## Conclusions

In this study, we have demonstrated the existence of conserved overlapping ORFs in the +1 frames of the vertebrate *ASXL1* and *ASXL2* genes. These “*TF* ORFs” coincide with highly significant synonymous site conservation in the corresponding zero-frame sequences, as is typically observed for dual coding regions, where selective constraints operate on more than one reading frame [28]. The *TF* ORFs are not canonically translatable within any known splice variants of the human *ASXL* genes. However, the presence of highly conserved motifs at the 5′ ends of the *TF* ORFs, identical to known +1/−2 PRF shift sites, suggests that the TF peptides may be expressed as transframe fusions with zero-frame ASXL via ribosomal frameshifting. The third paralogue, *ASXL3*, is more divergent and has neither a conserved long overlapping +1 frame ORF nor a conserved +1-frame EHxY motif.

## Methods

The initial set of 37,257 human mRNA RefSeqs was downloaded from NCBI in January 2010.

To identify further orthologues of *ASXL1* and *ASXL2*, tblastn searches of the nr/nt NCBI database were carried out, using selected reference species for each major vertebrate clade. For clades with poor representation in the nr/nt database, sequences from the transcriptome shotgun assembly (TSA) database were added, where available. To avoid the detection of non-orthologous genes, a minimum query coverage threshold of 80% was used to filter all results. Nucleotide sequences for each gene were retrieved from NCBI. RefSeq entries with missing or incomplete sequence data that precluded further analyses were discarded. Where multiple transcript isoforms were present for a given species, the lowest numbered isoform was used for analyses.

The *ASXL2* gene predictions of many sauropsids (reptiles and birds) have large (~150 aa) N-terminal deletions relative to those of all other major vertebrate clades. Because we set a minimum query coverage threshold of 80%, these genes were excluded from our analyses. However, examination of the relevant genomic and transcriptomic sequences shows that all contain conserved copies of the frameshift site – with the exception of lizards, as discussed above – and large *TF* ORFs in the expected frames.

For each of *ASXL1* and *ASXL2*, full-length zero-frame coding nucleotide sequences were translated, aligned as amino acids with MUSCLE [64], and the amino acid alignments were used to guide codon-based nucleotide alignments using EMBOSS tranalign [65]. The zero-frame sequences of all mRNAs were scanned for the relevant +1 or −2 PRF sites. Sequence alignments were analysed for synonymous site conservation using synplot2 [28] with amino acid PhyML guide trees [66]; for these analyses, alignments were mapped to reference sequence coordinates by removing alignment columns that contained a gap character in the reference sequence (mammalian/tetrapod, sauropsid, amphibian and teleost reference sequences respectively ASXL1: NM_015338 – *Homo sapiens*, XM_015296597 – *Gallus gallus*, XM_012952772 – *Xenopus tropicalis*, XM_005162338 – *Danio rerio*, and ASXL2: NM_018263 – *Homo sapiens*, NM_001031096 – *Gallus gallus*, XM_018089999 – *Xenopus tropicalis*).

For sequence logos, we selected subsets of sequences (76 for *ASXL1* and 52 for *ASXL2*; see Additional file 1) that more uniformly covered the sampled vertebrate phylogeny, to enable a representative assessment of nucleotide and amino acid composition. Sequence logos for visualization of amino acid conservation within the TF peptides, and nucleotide conservation at the putative frameshift sites, were created using WebLogo [31]. The Predictor of Natural Disordered Regions (PONDR®) [67] was used to predict disordered regions within the ASXL and ASXL-TF proteins, using the VL-XT algorithm.

To search for potentially stable RNA structures adjacent to putative frameshift sites, we extracted the 120-nt regions downstream of all putative shift sites and aligned them using Clustal Omega [68]. Consensus structures from those alignments were predicted using RNAalifold [69]. We also scanned each individual sequence for potential pseudoknots using PKNOTS [70].

RiboSeq datasets were retrieved from the NCBI short reads archive (accessions SRR2733100, SRR1573934 and SRR1573935 for Jurkat RiboSeq, MDA-MB-231 RiboSeq and MDA-MB-231 RNASeq, respectively) and mapped to human rRNA, then to the *ASXL1* and *ASXL2* transcipts (NM_015338.5 and NM_018263.4 respectively). Reads were mapped using bowtie version 1 [71], with parameters -v 2 --best (i.e. maximum 2 mismatches, report best match). Ribosome footprint densities were calculated for the regions upstream and downstream of the *TF* ORF, excluding five codons proximal to the start and stop codons and the frameshift site. Footprints were counted as mapping to this region if the 5′ end coordinate with a +12 nt offset (the approximate ribosome P-site position) mapped within this region.

To identify *ASXL1 TF*-region indels in the Gawron et al. Jurkat RiboSeq dataset, all 15-mers from 330 nt upstream of the *TF* ORF to 74 nt downstream of the *TF* ORF were queried against all post-rRNA subtraction sequencing reads. The resulting reads were inspected by blast [72] (blastn to *ASXL1* mRNA, selection of alignments with >0 gaps) and velvet [73] (de novo assembly with velvet and blastn of contigs to *ASXL1* mRNA). Once the two indels had been identified, the wildtype and mutant sequences at each site were used to extract and count the number of raw reads containing the wildtype or mutant sequences. Genomic DNA sequencing of Jurkat cell NCBI short read archive datasets SRX2596625 and SRX2596624 were queried using NCBI blastn with parameters, algorithm = blastn, max target sequences = 500, word size = 15, no low complexity filtering, and query = NM_015338.5 nt 2228–2426 (i.e. the region between the two indels plus 60 nt on either side), either wildtype sequence or the sequence with the two indel mutations, and the results inspected for presence/absence of indels.

## Reviewers’ comments

### Reviewer’s report 1: Eugene Koonin, NCBI, NLM, NIH, USA

The manuscript by Dinan et al. reports a previously unnoticed programmed frameshift in ASXL, an important human gene, and make inferences regarding the functionality of the frameshift proteins and the effects of its disruption by mutation. The analysis is done very carefully and discussed thoroughly, so I have no substantial criticisms. My only concern is that the manuscript is far too long for a Discovery Note. I think it has to be a regular research article, which will involve some restructuring, particularly, in the Abstract.

Authors’ response: *We thank Professor Koonin for his positive assessment of the manuscript. We have now restructured the manuscript as a regular research article.*

### Reviewer’s report 2: Laurence Hurst, University of Bath, UK

Summary: An interesting possible example of programmed frame shifting in vertebrates. The evidence is consistent but a lack of direct evidence of the short form of the protein and of its functionality render the case only partially made. Possible alternative interpretations of the data cannot yet be fully discounted.

Authors’ response: *We agree that other possible explanations cannot be completely discounted pending experimental work. However we were cautious in our assessment of the evidence and avoided making definitive statements about frameshifting, instead opting for terms such as “hypothesis”, “putative”, “most plausible”,* etc. *We also carefully considered possible alternative explanations in the paragraph beginning “We considered possible explanations for TF ORF expression, other than ribosomal frameshifting”.*

After an initial trawl for vertebrate sequences containing a conserved motif associated with programmed frameshifting in viruses, the authors identify a gene that looks like it might use this mechanism (ASXL1). A further member of the gene family (ASXL2) might similarly use programmed frameshifting but employing a different motif. We can question whether there are alternative interpretations of the data as presented and what other evidence would both make a more convincing case that the frameshifted protein is made and if made, functional. Evidence for the programmed frameshift presented by the authors is suggestive but not definitive.

Authors’ response: *Only experimental verification will provide definitive evidence. This will be difficult if the frameshifting efficiency is of order 1–2%, as predicted from the nature of the shift site. Given that the bioinformatic observation has great relevance to understanding the disease phenotype of ASXL truncation mutants, we feel that there is merit in timely publication of the bioinformatic observations.*

It is hard to know what to make of the evidence that one other member of the family has a different motif associated with frameshifts – could this be an ascertainment bias? How often would you find such motifs if you looked at a random gene?

Authors’ response: *From a set of 9736 human-chimpanzee-cow-mouse-chicken orthologues we found 25 genes with a G_GUC_UCU sequence conserved between human and chicken (MUSCLE amino acid alignment, back-translated to codon alignment). Only eight of these were also conserved in cow, chimpanzee and mouse. One of these is ASXL2.*

*It is true that there are other potential −2 frameshift motifs, although all known ones in vertebrate systems (*i.e. *those identified in arterivirus species) contain UU_UUU, UC_UCU or UU_UUC for codon:anticodon re-pairing in the A-site following a −2 frameshift* [1]. *As can be seen from* Fig. 2b *, the G_GUC_UCU stands out alone as being completely conserved while adjacent codon positions show variation. One may say that of 61 × 61 possible non-stop codons at these two adjacent sites, only 12 in 3721 combinations (*i.e. *NUC_UCU, NUU_UUU, NUU_UUC) would give one of the known viable A-site −2 slippage pentanucleotides, while only 4 in 3721 (*i.e. *UUU_CGN) would give rise to the UUU_CG that is the core of the only known mammalian +1 frameshift pattern that does not require a stop codon in the zero-frame A-site as in the antizyme genes* [6]*.*

While it looks like a coincidence too strong to be dismissed as an accident, it is curious that the usual RNA structures associated with frameshifts aren’t seen (but these can be quite far away [1]).

Authors’ response: *On the contrary, like many +1 frameshift sites, the influenza virus UCC_UUU_CGU +1 frameshift site apparently has no stimulatory RNA secondary structure* [1, 7]*. Similarly, the only confirmed case of −2 frameshifting on a G_GUC_UCU sequence (*i.e. *in arteriviruses) has no stimulatory RNA secondary structure* [1, 4]*.*

The strongest evidence that there is a functional frameshifted protein produced is all indirect: the conservation of synonymous sites in the zero (normal) reading frame and a conserved protein-level motif in the frameshifted sequence. In addition, the frameshifting 5′ motif is also conserved.

Authors’ response: *And also the conserved presence of a lengthy overlapping ORF across the entire vertebrate lineage in ASXL1, and across vertebrates except teleost fish in ASXL2.*

Conservation of the frameshift initiation motifs in ASXL1 is supportive, but this is also how this site was identified so is not independent evidence.

Authors’ response: *The site was identified due to its conservation in just human, mouse, chimpanzee, cow and chicken, but was then found to be conserved in 200 species spanning mammals, birds, reptiles, amphibians, teleost fish, shark, gar, and coelacanth.*

Moreover with motifs this small other explanations could be considered, such as miRNA pairing sites [74], transcriptional enhancers or RNA binding protein motifs in the zero frame [75–78].

Authors’ response: *The conserved presence of the lengthy overlapping TF ORF across the entire vertebrate lineage argues for a translational explanation. Given the absence of suitable AUG initiators in the TF ORF, and the presense of a conserved ribosomal frameshift site corresponding precisely to the 5′-most peak in synonymous site conservation (*Additional file 1 *: Figure S3), the simplest explanation is ribosomal frameshifting. We agree that, pending experimental verification, other possibilities cannot be ruled out.*

Likewise, can we be confident that the putative conserved C terminal motif in the non-zero frame is not similarly constrained in the zero frame that would result in off frame apparent protein conservation?

Authors’ response: *This was discussed in the manuscript – see the text: “The EH[N/S]Y motifs also coincide with distinct peaks in synonymous site conservation (*Fig. 3 *and* Additional file 1 *: Figure S3) indicating that these sites are subject to particularly strong evolutionary constraints. A zero-frame amino acid sequence corresponding to +1 frame EH[N/S]Y is necessarily highly constrained – for example, the first two positions can only be R/G and T/A respectively; yet all four amino acids were common at these positions, for example human ASXL1 and ASXL2 have zero-frame RTQLL and GAQLQ respectively at this site, confirming that conservation of +1 frame EH[N/S]Y is not due to zero-frame coding constraints.”*

The interpretation of the conservation of synonymous sites in the zero frame is yet more complex as the expected TF protein end is computationally predicted to be disordered (sort of what you might expect if this was not a programmed frameshift but instead just part of the rubbish that cells produce all the time or a computational artefact).

Authors’response: *On the contrary, overprinted genes often tend to have relatively large amounts of predicted disorder* [79]*. It seems that the dual coding constraints imposed by overlapping genes makes them more amenable to evolving within disordered regions, or to themselves contain disordered regions.*

If it is disordered, why need there be selection on the frameshift protein to impact zero frame synonymous sites, as disordered domains tend to be under much weaker purifying selection [80]?

Authors’ response: *“Disorder” is not the same as “completely random”. Even disordered regions of proteins can be subject to purifying selection, and disordered regions contain locally ordered regions and/or other functionally important sequences. Note in particular that the conserved HCF-1 binding motif in TF corresponds precisely to one of the peaks in synonymous site conservation within the overlapping ORF region (*Additional file 1 *: Figure S3).*

Further, a closer look at synonymous site conservation in ASXL2 suggests a section of the overlap might have lower (not higher) than than expected synonymous site conservation (Fig. 3a and b, right panel). Indeed, in both genes synonymous site conservation appears to be weakened at the central overlap section. This would fit either with the authors’ model and reduced constraint on disordered proteins, or with the possibility that there is selection on the zero phase at DNA or RNA level at a few intragene locations one of which happens to include a putative frameshifting motif (which may or may not initiate frameshifting).

Authors’ response: *Despite the lack of purifying selection on the middle region of the TF ORF (which could be due to it being a linker region) there is still a conserved absence of +1 frame stop codons in this region.*

The authors argue against the presence of an out of frame alternative splice form and against a different initiation site. Elimination of alternative splice forms as an explanation requires much deep sequencing and in the end would rely on evidence of absence. Transcriptomics is pretty deep in humans so absence of evidence is pretty good evidence.

Regarding, the alternative initiation site, they presume that transcripts must start with ATG, but nonATG initiation is known [81]. Can this be ruled out? Indeed, nonATG starts are associated with viral expression [3] (HERVH-associated [82] ESRG for example uses a non-canonical start codon [83]).

Authors’ response: *We are very familiar with references* [81] *and* [3] *and certainly did not forget the possibility of non-AUG initiation. However a long-winded discussion of presence or absence, and conservation and context of every “standard” non-AUG initiator (*i.e. *CUG, GUG, UUG, ACG, AUA, AUU, AUC), besides the possibility of non-standard non-AUG initiators in the context of dicistrovirus-like IRESes* [84]*, would not add clarity to the manuscript. In the absence of evidence for a suitable transcript where such a codon would be accessible* via *5′-end-dependent scanning, independent initiation in the TF frame is unlikely. The note on lack of suitable AUG initiators only serves to reinforce this. Of note, the highly conserved frameshift sites which correspond closely to the 5′-most peak in synonymous site conservation (*Additional file 1 *: Figure S3) do not contain any of the standard non-AUG initiators.*

I note too that nonATG starts can be associated with CAG repeats [85] one of which (CAG)_4_ occurs at position 76,695 in the genomic sequence (in exon 9). Whether this is anything other than a spurious finding is hard to say, but I don’t think unusual initiation can be so easily dismissed.

Authors’ response: *Being in exon 9, the (CAG)*_*4*_ *site in question is not relevant since there are many intervening stop codons between here and the TF ORF. Any +1 frame initiation here would not allow access to the TF ORF. Also, on the canonical ASXL1 transcript, there are no fewer than 13 AUG codons (in human) between the ASXL1 initiation AUG and the (CAG)*_*4*_ *, thus it is hard to imagine how pre-initiation scanning ribosomes could come anywhere near the (CAG)*_*4*_ *sequence.*

If the authors consider it possible that the sequences might make use of one viral trick – frameshifting – why could an alternative viral trick not be an equally good (or at least alternative) explanation?

Authors’ response: *As explained above, potential non-AUG initiation would require an independent transcript, likely with no +0 or +2 frame AUGs upstream of a potential +1 frame non-AUG initiator, and in addition one would need to propose that the conserved shift sites serve some other unknown purpose.*

Evidence from deep transcriptomics to exclude a transcript running across the putative frameshift but not much further 5′ would strengthen the case for a programmed frameshift rather than alternative initiation site.

Authors’ response: *Unfortunately if one starts to look for very rare transcripts in the public databases one will (i) not be able to conclude whether they are complete transcripts or just fragments, and (ii) enter the realm of transcriptional noise and experimental artifact.* E.g. *a common mutation in ASXL1 is an insertion of an additional G in a GGGGGGGG sequence near the 3′ end of the TF region (a deletion at this site would allow access to the C-terminus of TF). However, ref.* [86] *comment that a substantial fraction of the published mutants at this site are actually due to sequencing errors presumably as a result of the homopolymeric run.*

In this context, I notice that if you do a BLASTP of the ASXL1 TF sequence (just the TF portion) you pick up the C terminal section of a rather short predicted protein in naked mole rats (EHB11723). This starts at position 282 in the 360 amino acid mole rat sequence, making this protein about half the length of the human putative ASXL1-TF and suggestive of the possibility of an alternative start codon.

Authors’ response: *This analysis was already discussed in the manuscript. We carried out searches* via *BLASTP of the NCBI non-redundant database using the human ASXL TF amino acid sequences as queries. Very few matches were found – just three hits for each of ASXL1 and ASXL2 TF, using default search parameters. In every case, the sequence of the hit was predicted computationally from the genomic sequence of that organism, and each of the associated genomic sequences contains large gap regions that likely confounded the automated gene annotation. These six predicted protein sequences are ASXL1 – aa EHH65433.1, Macaca fascicularis, DNA CM001285.1; aa EHB11723.1, Heterocephalus glaber, DNA JH171429.1 (naked mole rat); and aa EPY85029.1, Camelus ferus, DNA KB016696.1; ASXL2 – aa XP_013150379.1, Falco peregrinus, DNA NW_004929857.1; aa XP_009330622.1, Pygoscelis adeliae, DNA NW_008825559.1; and aa OBS64385.1, Neotoma lepida, DNA LZPO01097212.1.*

*In the naked mole rat hypothetical protein sequence, EHB11723, the annotation starts in the zero-frame close to the canonical ASXL1 initiation site and enters the C-terminal half of TF* via *a predicted splice. So this has nothing to do with “an alternative start codon” and also doesn’t explain the synonymous site conservation and conserved absence of stop codons in the 5′ half of the TF region.*

*Sequencing databases are now so large that for many analyses one will pick up a number of aberrant sequences in isolated taxa, but these cannot be taken as evidence for an alternative mechanism unless the same features are conserved among multiple species.*

However, I see nothing in ENSEMBL that suggests an abbreviated transcript in human ASXL1, but for ASXL2 they annotate a transcript with an earlier initiation site with 10 rather than 13 exons and a smaller protein (ENST00000404843.5). It would be good if the authors can provide a more uptodate analysis of the various annotated forms and how they relate to the various hypotheses.

Authors’ response: *The ORF in this transcript misses the first 260 codons of the main ORF in NM_018263 (human ASXL2 NCBI RefSeq) and also 259 codons from the last exon of the RefSeq due to an additional splicing event. It does not however provide non-frameshifting access to the TF ORF. This transcript form appears to be based on the single cDNA clone BC042999 and the splice junction is not supported by EST or TSA databases, so it may be aberrant. Since it is not relevant to TF ORF expression we chose not to mention it in the manuscript.*

*We retrieved all currently annotated splice forms for Homo sapiens from the most recent release of the NCBI RefSeq database (* *ftp://ftp.ncbi.nlm.nih.gov/genomes/refseq/vertebrate_mammalian/Homo_sapiens/latest_assembly_versions/GCF_000001405.37_GRCh38.p11/* *; July 2017 ). In total, there are six isoforms annotated for each protein. These isoforms encode proteins with lengths ranging from 1313 aa to 1628 aa for ASXL1, and from 1175 aa to 1435 aa for ASXL2, owing largely to several distinct initiation sites being annotated. Crucially, in both cases, variation in amino acid sequence is restricted exclusively to N-terminal regions, far from the TF region. For ASXL1, all sequences are identical from ~300 aa upstream of the putative frameshift site until the end of the protein; while for ASXL2, all sequences are identical from ~370 aa upstream of the putative frameshift site until the end of the protein.*

To some degree all these issues would be irrelevant were there direct evidence for the truncated form of the protein and for its functionality. More generally then, the most obvious omission in the analysis is the lack of more direct evidence that the truncated proteins, as predicted by the frameshifting hypothesis, are produced and functional. The Riboseq data is cautiously interpreted by the authors (to their credit) and doesn’t appear decisive. I agree to that if the frameshift is seen only 1–2% of the time, as in viruses, that Riboseq may well not be refined enough to detect this. Is there no mass spec data to support the presence of the short form protein?

Authors’ response: *Again, the likely 1–2% level of frameshifting would make it very difficult if not impossible to detect TF peptides in publicly available whole-proteome mass spectrometry databases. To find previously unknown peptides – such as from TF – also requires access to raw mass spectra. Even where these are available, we do not have access to corresponding DNA sequencing to rule out possible somatic mutations (*cf. *the section in the manuscript on public ribosome profiling data).*

Even if the frameshift occurs, it could be caught by NMD, the rules for which remain poorly understood [87]. The authors note that as the stop of the frameshift is in the terminal exon in both genes NMD is not expected detect the new stop of the highly abbreviated CDS under normal understanding of NMD [87]. However, the 10 exon form noted above appears to split the last exon into two small exons, meaning a frameshift stop could have the potential to be in the last but one exon and hence caught by NMD.

Authors’ response: *The canonical splice form would still be expected to be resistant to NMD even if this possible alternative transcript form is not.*

It would then be good to see that knockdown of NMD doesn’t lead to upregulation of transcripts of the two genes, but, again, with a low frameshifting rate this may be too subtle to detect. Assuming the protein is made it would be good to have direct evidence for the putative novel interactions from protein interaction data. Indeed, a lovely experiment would be to use HCF-1 as a bait and see if you can extract the TF proteins.

Authors’ response: *Indeed this potential experiment had not escaped our notice.*

To date BioGrid doesn’t mention any such interaction, although it does mention a weak interaction with both ASXL1 and ASXL2.

Authors’ response: *The fact that ASXL-TF is not, at the time of review, in the public domain provides an easy explanation as to why its interactions are not mentioned in BioGrid.*

In conclusion the frameshift model is a parsimonious model to explain the observations, but cannot be considered as more than an hypothesis at present.

Authors’ response: *We agree, and that is the light in which we have presented it.*

## Additional file

Additional file 1: Supplementary figures and supplementary files. (PDF 2721 kb)

## Abbreviations

ASXH: ASX homology
ASXL: Additional Sex Combs-Like
BAP1: BRCA1 Associated Protein 1
ETP: Enhancer of thithorax and polycomb
HARE-HTH: HB1, ASXL, restriction endonuclease helix-turn-helix
HBM: HCF-1 binding motif
HCF-1: Host Cell Factor-1
NCBI: National Center for Biotechnology
ORF: open reading frame
PCBP: Poly(C) Binding Protein
PHD: Plant homeodomain
PR-DUB: Polycomb repressive deubiquitinase
PRF: Programmed ribosomal frameshifting
TF: Transframe
TSA: Transcriptome shotgun assembly
UTR: Untranslated region
