# Supplementary material for: ASXL gain-of-function truncation mutants: defective and dysregulated forms of a natural ribosomal frameshifting product?
Source: Biol Direct. 2017 Oct 16;12:24. doi: 10.1186/s13062-017-0195-0 (PMC5644247; doi:10.1186/s13062-017-0195-0)
Supplement: Additional file 1: — Supplementary figures and supplementary files. (PDF 2721 kb) [file 13062_2017_195_MOESM1_ESM.pdf]

Human *ASXL1* (GenBank accession NM_015338)

Zero-frame product (ASXL1)

MKDKQKKKKERTWAEAARLVLENYSDAPMTPKQILQVIEAEGLKEMRSGTSPLACLNAMLHSNSRGGEGLFYKLPGRISLFTLKKDALQW
SRHPATVEGEEPEDTADVESCGSNEASTVSGENDVSLDETSSNASCSTESQSRPLSNPRDSYRASSQANKQKKKTGVMLPRVVLTPLKVN
GAHVESASGFSGCHADGESGSPSSSSSGSLALGSAAIRGQAEVTQDPAPLLRGFRKPATGQMKRNRGEEIDFETPGSILVNTNLRALINS
RTFHALPSHFQQQLLFLLPEVDRQVGTDGLLRLSSSALNNEFFTHAAQSWRERLADGEFTHEMQVRIRQEMEKEKKVEQWKEKFFEDYYG
QKLGLTKEESLQQNVGQEEAEIKSGLCVPGESVRIQRGPATRQRDGHFKKRSRPDLRTRARRNLYKKQESEQAGVAKDAKSVASDVPLYK
DGEAKTDPAGLSSPHLPGTSSAAPDLEGPEFPVESVASRIQAEPDNLARASASPDRIPSLPQETVDQEPKDQKRKSFEQAASASFPEKKP
RLEDRQSFRNTIESVHTEKPQPTKEEPKVPPIRIQLSRIKPPWVVKGQPTYQICPRIIPTTESSCRGWTGARTLADIKARALQVRGARGH
HCHREAATTAIGGGGGPGGGGGGATDEGGGRGSSSGDGGEACGHPEPRGGPSTPGKCTSDLQRTQLLPPYPLNGEHTQAGTAMSRARRED
LPSLRKEESCLLQRATVGLTDGLGDASQLPVAPTGDQPCQALPLLSSQTSVAERLVEQPQLHPDVRTECESGTTSWESDDEEQGPTVPAD
NGPIPSLVGDDTLEKGTGQALDSHPTMKDPVNVTPSSTPESSPTDCLQNRAFDDELGLGGSCPPMRESDTRQENLKTKALVSNSSLHWIP
IPSNDEVVKQPKPESREHIPSVEPQVGEEWEKAAPTPPALPGDLTAEEGLDPLDSLTSLWTVPSRGGSDSNGSYCQQVDIEKLKINGDSE
ALSPHGESTDTASDFEGHLTEDSSEADTREAAVTKGSSVDKDEKPNWNQSAPLSKVNGDMRLVTRTDGMVAPQSWVSRVCAVRQKIPDSL
LLASTEYQPRAVCLSMPGSSVEATNPLVMQLLQGSLPLEKVLPPAHDDSMSESPQVPLTKDQSHGSLRMGSLHGLGKNSGMVDGSSPSSL
RALKEPLLPDSCETGTGLARIEATQAPGAPQKNCKAVPSFDSLHPVTNPITSSRKLEEMDSKEQFSSFSCEDQKEVRAMSQDSNSNAAPG
KSPGDLTTSRTPRFSSPNVISFGPEQTGRALGDQSNVTGQGKKLFGSGNVAATLQRPRPADPMPLPAEIPPVFPSGKLGPSTNSMSGGVQ
TPREDWAPKPHAFVGSVKNEKTFVGGPLKANAENRKATGHSPLELVGHLEGMPFVMDLPFWKLPREPGKGLSEPLEPSSLPSQLSIKQAF
YGKLSKLQLSSTSFNYSSSSPTFPKGLAGSVVQLSHKANFGASHSASLSLQMFTDSSTVESISLQCACSLKAMIMCQGCGAFCHDDCIGP
SKLCVLCLVVR

Frameshift product (ASXL1-TF; TF sequence in red)

MKDKQKKKKERTWAEAARLVLENYSDAPMTPKQILQVIEAEGLKEMRSGTSPLACLNAMLHSNSRGGEGLFYKLPGRISLFTLKKDALQW
SRHPATVEGEEPEDTADVESCGSNEASTVSGENDVSLDETSSNASCSTESQSRPLSNPRDSYRASSQANKQKKKTGVMLPRVVLTPLKVN
GAHVESASGFSGCHADGESGSPSSSSSGSLALGSAAIRGQAEVTQDPAPLLRGFRKPATGQMKRNRGEEIDFETPGSILVNTNLRALINS
RTFHALPSHFQQQLLFLLPEVDRQVGTDGLLRLSSSALNNEFFTHAAQSWRERLADGEFTHEMQVRIRQEMEKEKKVEQWKEKFFEDYYG
QKLGLTKEESLQQNVGQEEAEIKSGLCVPGESVRIQRGPATRQRDGHFKKRSRPDLRTRARRNLYKKQESEQAGVAKDAKSVASDVPLYK
DGEAKTDPAGLSSPHLPGTSSAAPDLEGPEFPVESVASRIQAEPDNLARASASPDRIPSLPQETVDQEPKDQKRKSFEQAASASFPEKKP
RLEDRQSF**VTQLKVFTPKSHSPLKRSPKSRPSGFNFHVSNHPGWLKVSPLTRYAPGSSPPRSPPAGVGLAPGPSQTLKPVLCRSEGREVT**
TAIERRPPLPSEGGVARVEVAAGPPMREVAEAAAVVMVVRPVATLSPGEARAPLESVRQIYSEHNYCRLIL

Human *ASXL2* (GenBank accession NM_018263)

Zero-frame product (ASXL2)

MREKGRRKKGRTWAEAAKTVLEKYPNTPMSHKEILQVIQREGLKEIRSGTSPLACLNAMLHTNSRGEEGIFYKVPGRMGVYTLKKDVPDG
VKELSEGSEESSDGQSDSQSSENSSSSSDGGSNKEGKKSRWKRKVSSSSPQSGCPSPTIPAGKVISPSQKHSKKALKQALKQQQQKKQQQ
QCRPSISISSNQHLSLKTVKAASDSVPAKPATWEGKQSDGQTGSPQNSNSSFSSSVKVENTLLGLGKKSFQRSERLHTRQMKRTKCADID
VETPDSILVNTNLRALINKHTFSVLPGDCQQRLLLLLPEVDRQVGPDGLMKLNGSALNNEFFTSAAQGWKERLSEGEFTPEMQVRIRQEI
EKEKKVEPWKEQFFESYYGQSSGLSLEDSKKLTASPSDPKVKKTPAEQPKSMPVSEASLIRIVPVVSQSECKEEALQMSSPGRKEECESQ
GEVQPNFSTSSEPLLSSALNTHELSSILPIKCPKDEDLLEQKPVTSAEQESEKNHLTTASNYNKSESQESLVTSPSKPKSPGVEKPIVKP
TAGAGPQETNMKEPLATLVDQSPESLKRKSSLTQEEAPVSWEKRPRVTENRQHQQPFQVSPQPFLNRGDRIQVRKVPPLKIPVSRISPMP
FHPSQVSPRARFPVSITSPNRTGARTLADIKAKAQLVKAQRAAAAAAAAAAAAASVGGTIPGPGPGGGQGPGEGGEGQTARGGSPGSDRV
SETGKGPTLELAGTGSRGGTRELLPCGPETQPQSETKTTPSQAQPHSVSGAQLQQTPPVPPTPAVSGACTSVPSPAHIEKLDNEKLNPTR
ATATVASVSHPQGPSSCRQEKAPSPTGPALISGASPVHCAADGTVELKAGPSKNIPNPSASSKTDASVPVAVTPSPLTSLLTTATLEKLP
VPQVSATTAPAGSAPPSSTLPAASSLKTPGTSLNMNGPTLRPTSSIPANNPLVTQLLQGKDVPMEQILPKPLTKVEMKTVPLTAKEERGM
GALIATNTTENSTREEVNERQSHPATQQQLGKTLQSKQLPQVPRPLQLFSAKELRDSSIDTHQYHEGLSKATQDQILQTLIQRVRRQNLL
SVVPPSQFNFAHSGFQLEDISTSQRFMLGFAGRRTSKPAMAGHYLLNISTYGRGSESFRRTHSVNPEDRFCLSSPTEALKMGYTDCKNAT
GESSSSKEDDTDEESTGDEQESVTVKEEPQVSQSAGKGDTSSGPHSRETLSTSDCLASKNVKAEIPLNEQTTLSKENYLFTRGQTFDEKT
LARDLIQAAQKQMAHAVRGKAIRSSPELFSSTVLPLPADSPTHQPLLLPPLQTPKLYGSPTQIGPSYRGMINVSTSSDMDHNSAVPGSQV
SSNVGDVMSFSVTVTTIPASQAMNPSSHGQTIPVQAFSEENSIEGTPSKCYCRLKAMIMCKGCGAFCHDDCIGPSKLCVSCLVVR

Frameshift product (ASXL2-TF; TF sequence in red)

MREKGRRKKGRTWAEAAKTVLEKYPNTPMSHKEILQVIQREGLKEIRSGTSPLACLNAMLHTNSRGEEGIFYKVPGRMGVYTLKKDVPDG
VKELSEGSEESSDGQSDSQSSENSSSSSDGGSNKEGKKSRWKRKVSSSSPQSGCPSPTIPAGKVISPSQKHSKKALKQALKQQQQKKQQQ
QCRPSISISSNQHLSLKTVKAASDSVPAKPATWEGKQSDGQTGSPQNSNSSFSSSVKVENTLLGLGKKSFQRSERLHTRQMKRTKCADID
VETPDSILVNTNLRALINKHTFSVLPGDCQQRLLLLLPEVDRQVGPDGLMKLNGSALNNEFFTSAAQGWKERLSEGEFTPEMQVRIRQEI
EKEKKVEPWKEQFFESYYGQSSGLSLEDSKKLTASPSDPKVKKTPAEQPKSMPVSEASLIRIVPVVSQSECKEEALQMSSPGRKEECESQ
GEVQPNFSTSSEPLLSSALNTHELSSILPIKCPKDEDLLEQKPVTSAEQESEKNHLTTASNYNKSESQESLVTSPSKPKSPGVEKPIVKP
TAGAGPQETNMKEPLATLVDQSPESLKRKSSLTQEEAPVSWEKRPRVTENRQHQQPFQVSPQPFLNRGDRIQVRKVPPLKIPVSRISPMP
FHPSQVS**LPGLVFQSPSLVLTEQEPELLQTSKQKPNWSKHRGQQLPLPPQLLQPPQLEGPFQDLAQGVDKVQERVVKGRLLEEAVQAQTE**
SVKLERAPHWNWQELEAGEVRESFYPVVQRLSPSLRPRPPQARHSLIVSLEHNYSKPPQCLQHLPSVEHAQVSHHQPT

Supplementary Figure 1. Amino acid sequences of human ASXL and ASXL-TF polypeptides.

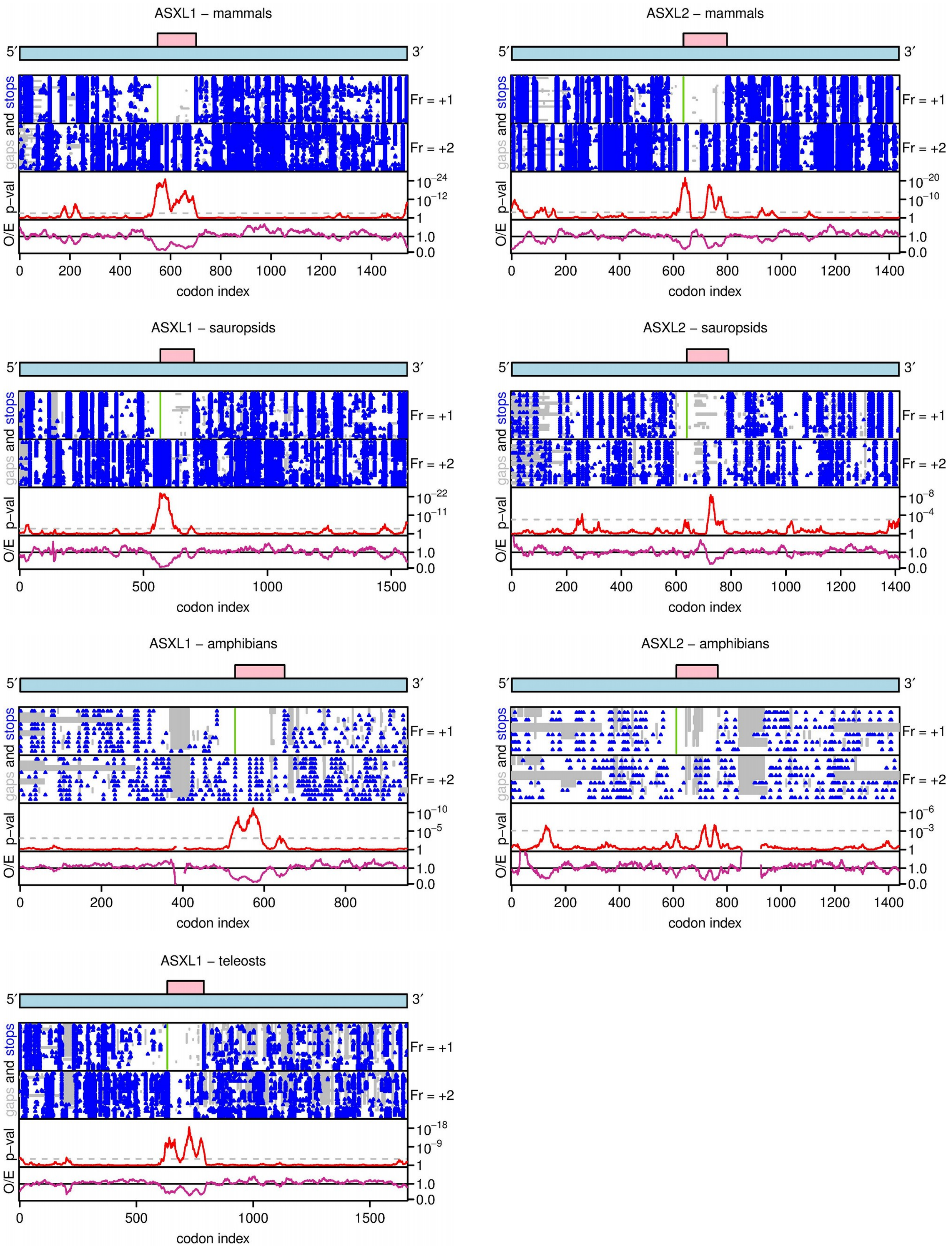

Supplementary Figure 2. Synonymous site conservation in the *ASXL1* and *ASXL2* coding regions in different vertebrate clades. In each subfigure, the top panel shows a schematic of the zero-frame ORF (pale blue) and the overlapping *TF* ORF (pink). The next two panels show positions of stop codons (blue) in the +1 and +2 reading frames, and alignment gaps (grey) in each

sequence of the sequence alignment. The vertical green line in the +1 frame panel shows the position of the putative frameshift site. The bottom two panels show the synonymous site conservation analysis, with the magenta line (lower panel) indicating the ratio of the observed number of substitutions within a given window to the number expected under a null model of neutral evolution at synonymous sites, and the red line (upper panel) showing the corresponding p-value. The analysis uses a 25-codon sliding window. The horizontal dashed grey line indicates a p = 0.05 threshold after a correction for multiple testing (namely scaling by [25-codon window size]/[*ASXL* CDS length].

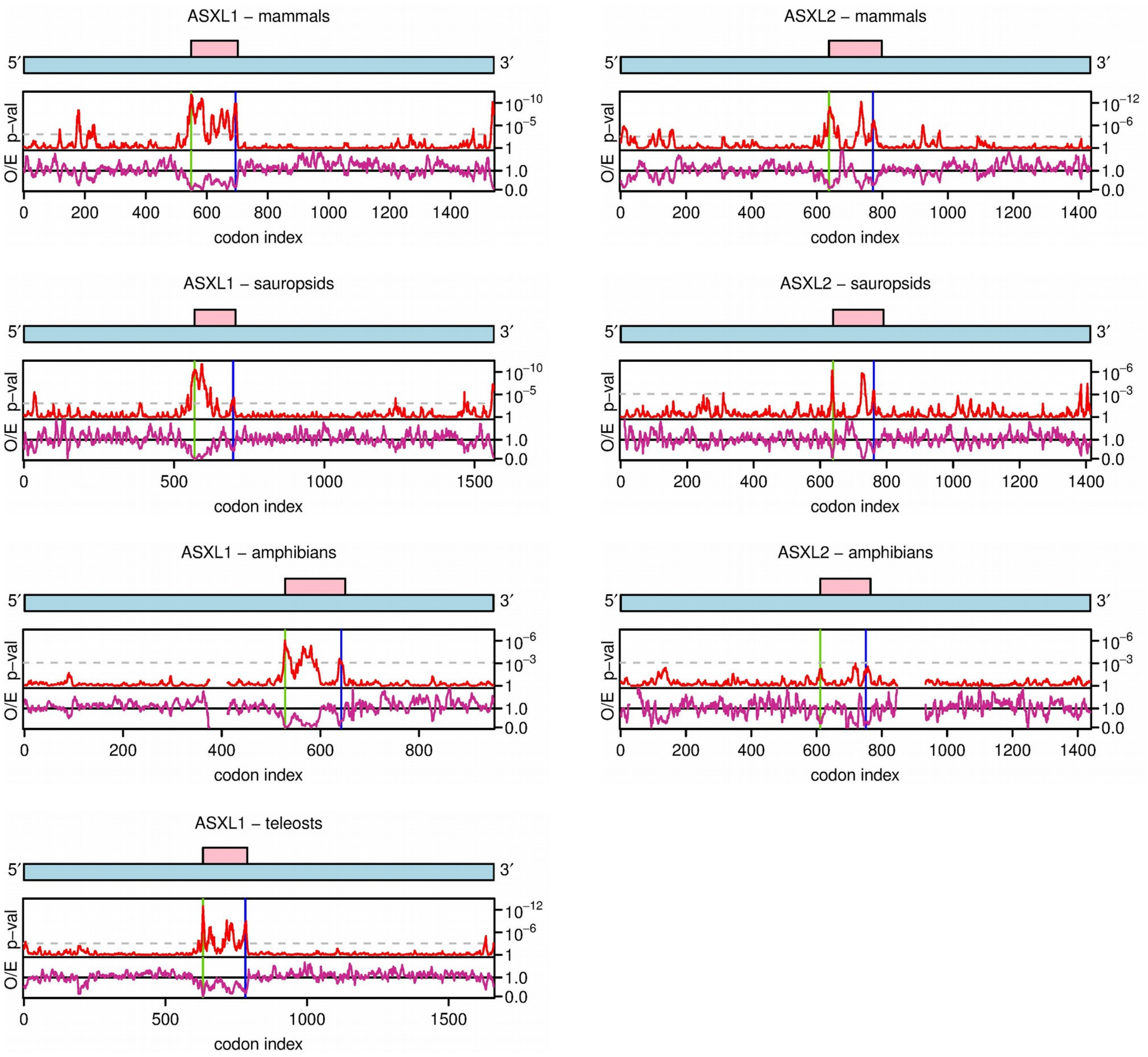

Supplementary Figure 3. Synonymous site conservation in the *ASXL1* and *ASXL2* coding regions in different vertebrate clades. In each subfigure, the top panel shows a schematic of the zero-frame ORF (pale blue) and the overlapping *TF* ORF (pink). The bottom two panels show the synonymous site conservation analysis, with the magenta line (lower panel) indicating the ratio of the observed number of substitutions within a given window to the number expected under a null model of neutral evolution at synonymous sites, and the red line (upper panel) showing the corresponding *p*-value. The analysis uses a 9-codon sliding window. The horizontal dashed grey line indicates a p = 0.05 threshold after a correction for multiple testing (namely scaling by [9-codon window size]/[*ASXL* CDS length]. The vertical green and blue lines show the positions of the putative frameshift site and the conserved EH[N/S]Y, respectively.

Human *ASXL1* (GenBank accession NM_015338) - *TF* ORF region

```
CUG AGC AGU CCC CAU CUG CCA GGC ACA UCC UCU GCA GCA CCC GAC CUG GAG GGU CCC GAA
UUC CCA GUU GAG UCU GUG GCU UCU CGG AUC CAG GCU GAG CCA GAC AAC UUG GCA CGU GCC
UCU GCA UCU CCA GAC AGA AUU CCU AGC CUG CCU CAG GAA ACU GUG GAU CAG GAA CCC AAG
GAU CAG AAG AGG AAA UCC UUU GAG CAG GCG GCC UCU GCA UCC UUU CCC GAA AAG AAG CCC
CGG CUU GAA GAU CGU CAG UCC UUU CGU AAC ACA AUU GAA AGU GUU CAC ACC GAA AAG CCA
CAG CCC ACU AAA GAG GAG CCC AAA GUC CCG CCC AUC CGG AUU CAA CUU UCA CGU AUC AAA
CCA CCC UGG GUG GUU AAA GGU CAG CCC ACU UAC CAG AUA UGC CCC CGG AUC AUC CCC ACC
ACG GAG UCC UCC UGC CGG GGU UGG ACU GGC GCC AGG ACC CUC GCA GAC AUU AAA GCC CGU
GCU CUG CAG GUC CGA GGG GCG AGA GGU CAC CAC UGC CAU AGA GAG GCG GCC ACC ACU GCC
AUC GGA GGG GGG GGU GGC CCG GGU GGA GGU GGC GGC GGG GCC ACC GAU GAG GGA GGU GGC
AGA GGC AGC AGC AGU GGU GAU GGU GGU GAG GCC UGU GGC CAC CCU GAG CCC AGG GGA GGC
CCG AGC ACC CCU GGA AAG UGU ACG UCA GAU CUA CAG CGA ACA CAA CUA CUG CCG CCU UAU
CCU CUA AAU
```

Human *ASXL2* (GenBank accession NM_018263) - *TF* ORF region

```
GUG AGC UGG GAG AAG AGG CCA CGU GUC ACU GAG AAU CGC CAG CAC CAG CAG CCA UUU CAG
GUC UCA CCA CAG CCC UUU CUC AAU AGA GGG GAC AGA AUC CAG GUG CGA AAA GUA CCA CCU
CUC AAG AUC CCG GUC UCC AGA AUC UCC CCC AUG CCG UUU CAU CCA UCG CAG GUC UCU CCC
AGG GCU CGU UUU CCA GUC UCC AUC ACU AGU CCU AAC AGA ACA GGA GCC AGA ACU CUU GCA
GAC AUC AAA GCA AAA GCC CAA CUG GUC AAA GCA CAG AGG GCA GCA GCU GCC GCU GCC GCC
GCA GCU GCU GCA GCC GCC UCA GUU GGA GGG ACC AUU CCA GGA CCU GGC CCA GGG GGU GGA
CAA GGU CCA GGA GAG GGU GGU GAA GGG CAG ACU GCU AGA GGA GGC AGU CCA GGC UCA GAC
AGA GUC AGU GAA ACU GGA AAG GGC CCC ACA CUG GAA CUG GCA GGA ACU GGA AGC AGG GGA
GGU ACG AGA GAG CUU UUA CCC UGU GGU CCA GAG ACU CAG CCC CAG UCU GAG ACC AAG ACC
ACC CCA AGC CAG GCA CAG CCU CAU AGU GUC UCU GGA GCA CAA CUA CAG CAA ACC CCC CCA
GUG CCU CCA ACA CCU GCC GUC AGU GGA GCA UGC ACA AGU GUC CCA UCA CCA GCC CAC AUA
GAG
```

UXX	Last upstream *TF*-frame stop codon
UXX	*TF* ORF stop codon
XXX	Putative frameshift site
AUG	*TF*-frame AUGs (none in *ASXL2*)

Supplementary Figure 4. Nucleotide sequences of the human *TF* ORF regions. Nucleotide sequences are shown for the region between the last upstream in-frame stop codon (orange) and the *TF* ORF stop codon (red); however ribosomes are expected to enter the *TF* frame at the putative frameshift sites (purple). Zero-frame codons are separated by spaces. The absence of *TF*-frame AUG codons in the *TF* region in *ASXL2* and except near the 3′ end of the *TF* ORF in *ASXL1*, argues against independent internal initiation in *TF*.

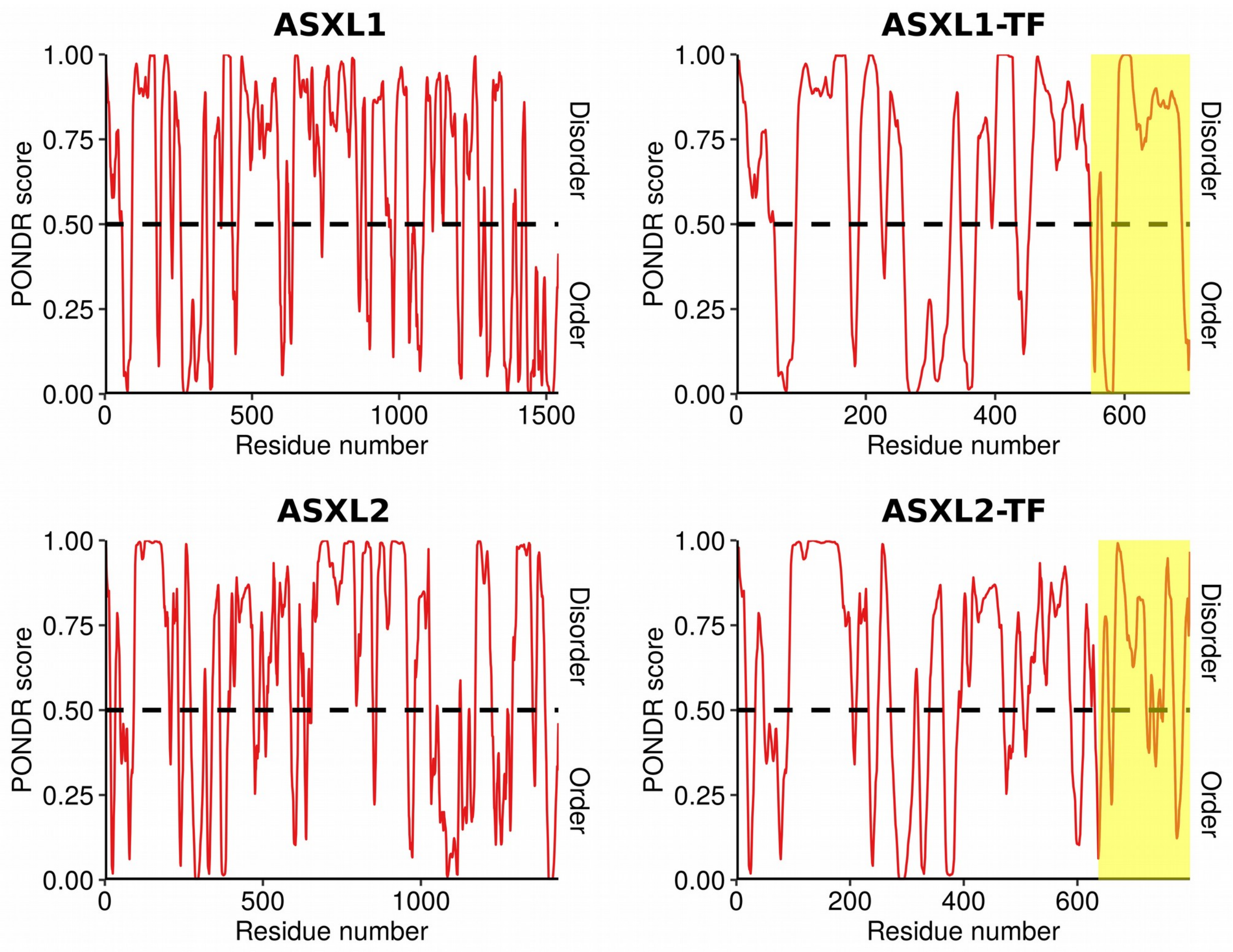

Supplementary Figure 5. Predicted ordered and disordered regions in human ASXL and ASXL-TF polypeptides. The TF region is highlighted in yellow.

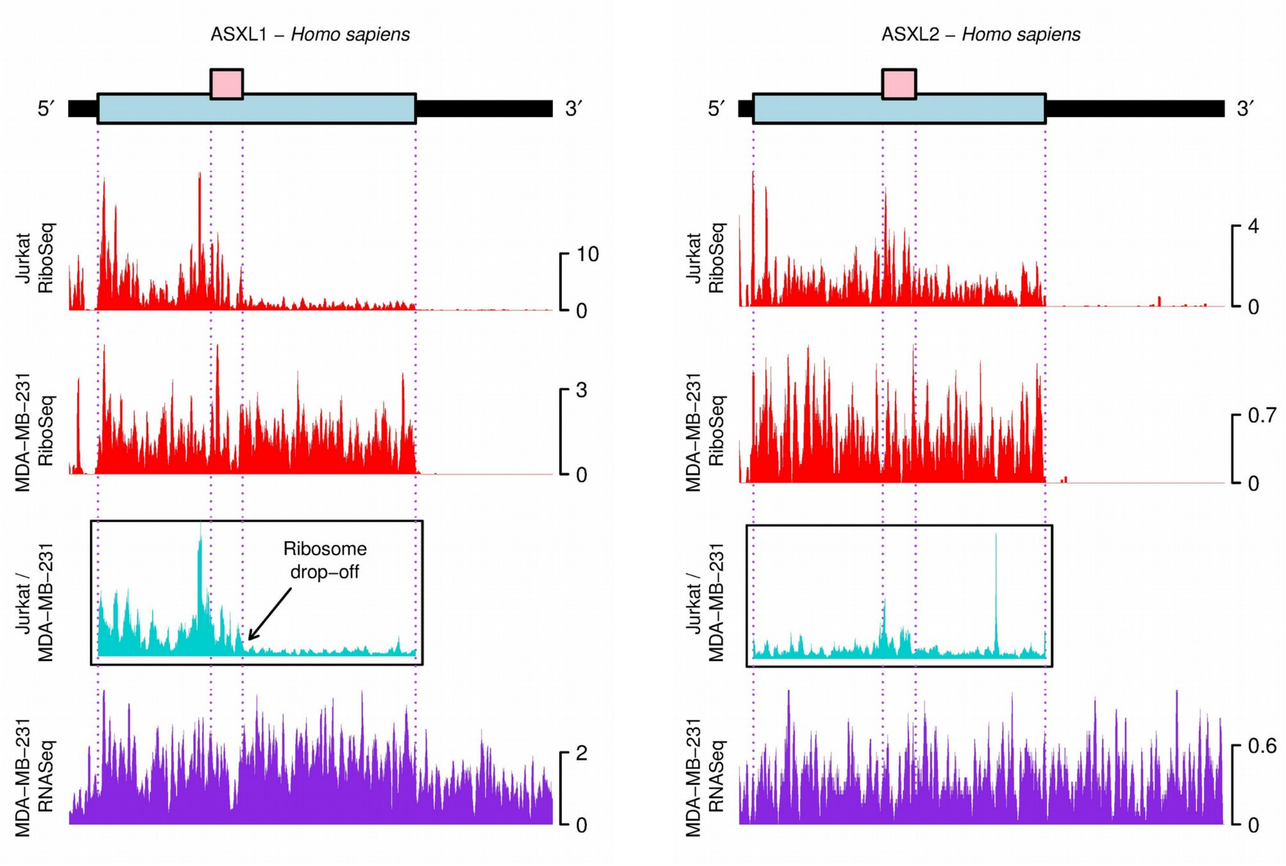

Supplementary Figure 6. Ribosome profiling analysis of human *ASXL*. Transcript maps of *ASXL1* (left) and *ASXL2* (right) are shown at top, with the main *ASXL* ORF in pale blue and the overlapping *TF* ORF in pink. Below, the first two tracks (red) show ribosome profiling from Jurkat T-lymphocyte cells and MDA-MB-231 breast cancer cells. The bottom track (purple) shows RNASeq from MDA-MB-231 breast cancer cells. Plots show histograms of the 5′ ends of reads with a +12 nt offset to map (for ribosome profiling) approximate P-site positions, and smoothed with a 31-nt running mean filter. The y-axis shows the mean read counts after smoothing. The inset plot (turquoise) shows the ratio, within the coding sequence, of the two ribosome profiling datasets after first applying a 61-nt running mean filter to each dataset. Note that the high levels of ribosome drop-off seen in *ASXL1* in Jurkat cells is likely to be a result of suspected somatic mutations in this cell-line rather than highly efficient ribosomal frameshifting (see text).

Supplementary File 1 - accession numbers of sequences used

The subsets of sequences (76 for *ASXL1* and 52 for *ASXL2*) selected to more uniformly cover the phylogeny for the sequence logo analyses are indicated with asterisks.

ASXL1

mammals

XM_015071869	*Acinonyx jubatus*
XM_011223720	*Ailuropoda melanoleuca*
XM_012455205	*Aotus nancymaae*
XM_007193384	*Balaenoptera acutorostrata scammoni*
XM_010841718	*Bison bison bison*
XM_019972246	*Bos indicus*
XM_005887939	*Bos mutus*
XM_600364	*Bos taurus*
XM_006064407	*Bubalus bubalis*
XM_017971854	*Callithrix jacchus*
XM_010960874	*Camelus bactrianus* *
XM_010993907	*Camelus dromedarius*
XM_014554657	*Camelus ferus*
XM_005634922	*Canis lupus familiaris* *
XM_018057698	*Capra hircus*
XM_008074492	*Carlito syrichta* *
XM_013159847	*Cavia porcellus*
XM_017514550	*Cebus capucinus imitator* *
XM_004442480	*Ceratotherium simum simum* *
XM_012052496	*Cercocebus atys*
XM_005384868	*Chinchilla lanigera*
XM_008020495	*Chlorocebus sabaeus*
XM_006860770	*Chrysochloris asiatica*
XM_011959875	*Colobus angolensis palliatus*
XM_004687292	*Condylura cristata* *
XM_016975522	*Cricetulus griseus*
XM_004464052	*Dasypus novemcinctus*
XM_013010673	*Dipodomys ordii* *
XM_013008731	*Echinops telfairi* *
XM_006881688	*Elephantulus edwardii* *
XM_008140954	*Eptesicus fuscus*
XM_014845061	*Equus asinus*
XM_005604562	*Equus caballus*
XM_008525258	*Equus przewalskii*
XM_016187970	*Erinaceus europaeus*
XM_019826661	*Felis catus*
XM_010628451	*Fukomys damarensis* *
XM_008577080	*Galeopterus variegatus*
XM_004061980	*Gorilla gorilla gorilla*
XM_004840576	*Heterocephalus glaber*
XM_019645788	*Hipposideros armiger*
NM_015338	*Homo sapiens* *
XM_013356440	*Ictidomys tridecemlineatus* *

XM_012951056	*Jaculus jaculus* *
XM_006740976	*Leptonychotes weddellii*
XM_007446853	*Lipotes vexillifer*
XM_010591603	*Loxodonta africana*
XM_005568667	*Macaca fascicularis*
XM_015149251	*Macaca mulatta*
XM_011766345	*Macaca nemestrina*
XM_011979776	*Mandrillus leucophaeus*
XM_017648617	*Manis javanica*
XM_015481723	*Marmota marmota marmota*
XM_005086071	*Mesocricetus auratus*
XM_012754776	*Microcebus murinus*
XM_005363226	*Microtus ochrogaster*
XM_016200632	*Miniopterus natalensis*
XM_007474452	*Monodelphis domestica* *
NM_001039939	*Mus musculus*
XM_013053414	*Mustela putorius furo*
XM_014550680	*Myotis brandtii*
XM_015566642	*Myotis davidii*
XM_014456449	*Myotis lucifugus*
XM_008834843	*Nannospalax galili*
XM_003273514	*Nomascus leucogenys*
XM_004585717	*Ochotona princeps* *
XM_004393041	*Odobenus rosmarus divergens*
XM_004272710	*Orcinus orca* *
XM_007934547	*Orycteropus afer afer*
XM_008256142	*Oryctolagus cuniculus* *
XM_012804162	*Otolemur garnettii*
XM_012151672	*Ovis aries musimon*
XM_015099825	*Ovis aries*
XM_003814739	*Pan paniscus*
XM_019458810	*Panthera pardus*
XM_015537063	*Panthera tigris altaica*
XM_005958892	*Pantholops hodgsonii* *
XM_016937663	*Pan troglodytes*
XM_009216344	*Papio anubis*
XM_006985536	*Peromyscus maniculatus bairdii*
XM_007122347	*Physeter catodon*
XM_012637708	*Propithecus coquereli*
XM_006922041	*Pteropus alecto*
XM_011380179	*Pteropus vampyrus*
XM_019748488	*Rhinolophus sinicus*
XM_017884118	*Rhinopithecus bieti*
XM_010352765	*Rhinopithecus roxellana*
XM_016148805	*Rousettus aegyptiacus* *
XM_003932071	*Saimiri boliviensis boliviensis*
XM_012553621	*Sarcophilus harrisii* *
XM_012933645	*Sorex araneus*
XM_005672837	*Sus scrofa* *
XM_004370455	*Trichechus manatus latirostris*
XM_014590207	*Tupaia chinensis* *
XM_019950775	*Tursiops truncatus*

XM_008703387	*Ursus maritimus*
XM_006202660	*Vicugna pacos*

sauropsids

XM_009084380	*Acanthisitta chloris* *
XM_019500328	*Alligator mississippiensis* *
XM_005011000	*Anas platyrhynchos* *
XM_008120859	*Anolis carolinensis* *
XM_013198496	*Anser cygnoides domesticus*
XM_009870040	*Apaloderma vittatum* *
XM_009279537	*Aptenodytes forsteri*
XM_013958321	*Apteryx australis mantelli* *
XM_011578340	*Aquila chrysaetos canadensis*
XM_010308576	*Balearica regulorum gibbericeps*
XM_010133415	*Buceros rhinoceros silvestris* *
XM_014956841	*Calidris pugnax*
XM_008497766	*Calypte anna*
XM_010162275	*Caprimulgus carolinensis*
XM_009694853	*Cariama cristata*
XM_009993988	*Chaetura pelagica*
XM_009893390	*Charadrius vociferus* *
XM_007062481	*Chelonia mydas*
XM_010120851	*Chlamydotis macqueenii*
XM_008172168	*Chrysemys picta bellii*
XM_005499680	*Columba livia*
XM_008634138	*Corvus brachyrhynchos*
XM_010396950	*Corvus cornix cornix*
XM_015881880	*Coturnix japonica*
XM_019539485	*Crocodylus porosus*
XM_009562634	*Cuculus canorus*
XM_009640313	*Egretta garzetta* *
XM_005444837	*Falco cherrug* *
XM_005239907	*Falco peregrinus*
XM_016303293	*Ficedula albicollis* *
XM_015296597	*Gallus gallus* *
XM_019509967	*Gavialis gangeticus*
XM_015426170	*Gekko japonicus* *
XM_005422200	*Geospiza fortis*
XM_009914948	*Haliaeetus albicilla*
XM_010569380	*Haliaeetus leucocephalus*
XM_017822613	*Lepidothrix coronata*
XM_018073998	*Manacus vitellinus*
XM_010722615	*Meleagris gallopavo*
XM_010178604	*Mesitornis unicolor*
XM_009471438	*Nipponia nippon*
XM_009933575	*Opisthocomus hoazin* *
XM_015647794	*Parus major* *
XM_009482169	*Pelecanus crispus*
XM_006117868	*Pelodiscus sinensis* *
XM_010283841	*Phaethon lepturus* *
XM_009511061	*Phalacrocorax carbo*
XM_005524727	*Pseudopodoces humilis*

XM_010084600	*Pterocles gutturalis* *
XM_009318746	*Pygoscelis adeliae*
XM_009093027	*Serinus canaria*
XM_014890102	*Sturnus vulgaris*
XM_002192704	*Taeniopygia guttata*
XM_009962456	*Tyto alba* *
XM_005491248	*Zonotrichia albicollis* *

amphibians

HADQ01029003	*Bombina bombina* *
GEGK01045837	*Leptobrachium boringii* *
GEGL01031110	*Megophrys* *
GECV01019509	*Microhyla fissipes* *
XM_018556374	*Nanorana parkeri* *
GEGH01003348	*Polypedates megacephalus* *
GDDO01068111	*Rana catesbeiana* *
GEGG01008506	*Rhacophorus dennysi* *
XM_018234430	*Xenopus laevis* *
XM_012952772	*Xenopus tropicalis* *

teleost fish

XM_007246841	*Astyanax mexicanus* *
XM_014031322	*Austrofundulus limnaeus*
XM_012820794	*Clupea harengus* *
XM_008318529	*Cynoglossus semilaevis* *
XM_015396824	*Cyprinodon variegatus* *
XM_005162338	*Danio rerio* *
XM_010875623	*Esox lucius* *
XM_012877575	*Fundulus heteroclitus* *
XM_005948017	*Haplochromis burtoni*
XM_019891036	*Hippocampus comes* *
XM_017487517	*Ictalurus punctatus* *
XM_017438828	*Kryptolebias marmoratus* *
XM_019259947	*Larimichthys crocea*
XM_018701579	*Lates calcarifer*
XM_004570926	*Maylandia zebra*
XM_006807161	*Neolamprologus brichardi*
XM_015967370	*Nothobranchius furzeri* *
XM_010784192	*Notothenia coriiceps* *
XM_005465660	*Oreochromis niloticus* *
XM_011473016	*Oryzias latipes* *
XM_020089223	*Paralichthys olivaceus*
XM_007575434	*Poecilia formosa*
XM_015048376	*Poecilia latipinna* *
XM_015004530	*Poecilia mexicana*
XM_008412961	*Poecilia reticulata*
XM_005752308	*Pundamilia nyererei*
XM_017708670	*Pygocentrus nattereri* *
XM_014135258	*Salmo salar* *
XM_016503785	*Sinocyclocheilus anshuiensis*
XM_016273902	*Sinocyclocheilus grahami*

XM_016513134	*Sinocyclocheilus rhinocerous* *
XM_008282558	*Stegastes partitus* *
XM_011619777	*Takifugu rubripes* *
XM_014475275	*Xiphophorus maculatus*

other

GEUG01028913	*Amia calva* *
XM_007885779	*Callorhinchus milii* *
XM_014498298	*Latimeria chalumnae* *
XM_015365106	*Lepisosteus oculatus* *

ASXL2

mammals

XM_015064116	*Acinonyx jubatus*
XM_019808476	*Ailuropoda melanoleuca*
XM_012453268	*Aotus nancymaae*
XM_007191266	*Balaenoptera acutorostrata scammoni*
XM_010840577	*Bison bison bison*
XM_019970597	*Bos indicus*
XM_005895687	*Bos mutus*
XM_002691461	*Bos taurus*
XM_006046228	*Bubalus bubalis*
XM_008981035	*Callithrix jacchus*
XM_010948539	*Camelus bactrianus* *
XM_010992973	*Camelus dromedarius*
XM_006190213	*Camelus ferus*
XM_003432160	*Canis lupus familiaris* *
XM_005686939	*Capra hircus*
XM_008063472	*Carlito syrichta* *
XM_003472847	*Cavia porcellus*
XM_017527302	*Cebus capucinus imitator* *
XM_004418269	*Ceratotherium simum simum* *
XM_012044335	*Cercocebus atys*
XM_005400412	*Chinchilla lanigera*
XM_007971691	*Chlorocebus sabaeus*
XM_006835227	*Chrysochloris asiatica*
XM_011950426	*Colobus angolensis palliatus*
XM_012727612	*Condylura cristata* *
XM_003497015	*Cricetulus griseus*
XM_013013735	*Dipodomys ordii* *
XM_004696413	*Echinops telfairi* *
XM_006880618	*Elephantulus edwardii* *
XM_008162352	*Eptesicus fuscus*
XM_014856740	*Equus asinus*
XM_001918196	*Equus caballus*
XM_008542771	*Equus przewalskii*
XM_007516745	*Erinaceus europaeus*
XM_006930426	*Felis catus*
XM_010604841	*Fukomys damarensis* *

XM_008577069	*Galeopterus variegatus*
XM_004028952	*Gorilla gorilla gorilla*
XM_004839122	*Heterocephalus glaber*
XM_019643761	*Hipposideros armiger*
NM_018263	*Homo sapiens* *
XM_005322571	*Ictidomys tridecemlineatus* *
XM_004663740	*Jaculus jaculus* *
XM_006732866	*Leptonychotes weddellii*
XM_007470793	*Lipotes vexillifer*
XM_003411920	*Loxodonta africana*
XM_005576367	*Macaca fascicularis*
XM_015111861	*Macaca mulatta*
XM_011738081	*Macaca nemestrina*
XM_011972683	*Mandrillus leucophaeus*
XM_017666555	*Manis javanica*
XM_015479905	*Marmota marmota marmota*
XM_005079112	*Mesocricetus auratus*
XM_012788149	*Microcebus murinus*
XM_005360858	*Microtus ochrogaster*
XM_016209133	*Miniopterus natalensis*
XM_001380127	*Monodelphis domestica* *
NM_001270988	*Mus musculus*
XM_004745948	*Mustela putorius furo*
XM_005874900	*Myotis brandtii*
XM_015566378	*Myotis davidii*
XM_014446882	*Myotis lucifugus*
XM_008834991	*Nannospalax galili*
XM_012497462	*Nomascus leucogenys*
XM_004582643	*Ochotona princeps* *
XM_004627442	*Octodon degus*
XM_004394909	*Odobenus rosmarus divergens*
XM_012532454	*Orcinus orca* *
XM_007666728	*Ornithorhynchus anatinus* *
XM_008254661	*Oryctolagus cuniculus* *
XM_003787447	*Otolemur garnettii*
XM_012158262	*Ovis aries musimon*
XM_004005731	*Ovis aries*
XM_003827052	*Pan paniscus*
XM_019450044	*Panthera pardus*
XM_005978907	*Pantholops hodgsonii* *
XM_016948189	*Pan troglodytes*
XM_003908363	*Papio anubis*
XM_006981457	*Peromyscus maniculatus bairdii*
XM_007108431	*Physeter catodon*
XM_012652406	*Propithecus coquereli*
XM_006910290	*Pteropus alecto*
XM_011356536	*Pteropus vampyrus*
XM_008764542	*Rattus norvegicus*
XM_019712755	*Rhinolophus sinicus*
XM_017876853	*Rhinopithecus bieti*
XM_010383018	*Rhinopithecus roxellana*
XM_016121738	*Rousettus aegyptiacus* *

XM_010345790	*Saimiri boliviensis boliviensis*
XM_012539839	*Sarcophilus harrisii* *
XM_013996276	*Sus scrofa* *
XM_004377581	*Trichechus manatus latirostris*
XM_006162196	*Tupaia chinensis* *
XM_004312104	*Tursiops truncatus*
XM_008700579	*Ursus maritimus*
XM_006197176	*Vicugna pacos*

sauropsids

XM_019497675	*Alligator mississippiensis* *
XM_014524044	*Alligator sinensis*
XM_013200279	*Anser cygnoides domesticus* *
XM_009275117	*Aptenodytes forsteri* *
XM_011598858	*Aquila chrysaetos canadensis*
XM_010142315	*Buceros rhinoceros silvestris* *
XM_014961567	*Calidris pugnax* *
XM_010002204	*Chaetura pelagica* *
XM_009890372	*Charadrius vociferus* *
XM_007062817	*Chelonia mydas*
XM_005284123	*Chrysemys picta bellii* *
XM_015859722	*Coturnix japonica*
XM_009563752	*Cuculus canorus* *
XM_014287174	*Falco cherrug* *
NM_001031096	*Gallus gallus* *
XM_010565910	*Haliaeetus leucocephalus* *
XM_017838512	*Lepidothrix coronata* *
XM_010708024	*Meleagris gallopavo* *
XM_005146457	*Melopsittacus undulatus* *
XM_009467461	*Nipponia nippon* *
XM_009943397	*Opisthocomus hoazin* *
XM_009909487	*Picoides pubescens* *
XM_007422356	*Python bivittatus* *
XM_009669652	*Struthio camelus australis* *

amphibians

GFBM010877296	*Ambystoma mexicanum* *
XM_018574722	*Nanorana parkeri* *
GEGH01064887	*Polypedates megacephalus* *
GDDO01077260	*Rana catesbeiana* *
XM_018264742	*Xenopus laevis* *
XM_018089999	*Xenopus tropicalis* *

other

XM_007900450	*Callorhinchus milii* *
XM_006005057	*Latimeria chalumnae* *
XM_015348384	*Lepisosteus oculatus* *

Supplementary File 2 - vertebrate *ASXL1* and *ASXL2* TF peptide sequences

ASXL1

Predicted ASXL1 TF peptide sequences following a +1 PRF at the conserved UCC_UUU_CGU sequence. The conserved EH[N/S]Y motif is highlighted in purple.

mammals

```
XM_012553621 Sarcophilus harrisii               VTQLKVFTPKSRSLLKRSPKSRPSGFNFHVSNRPGWLKVSPLTRYAPALSPTQNTPAGAELVPELSQTLRPALCKPEPR------EKL--PQPPLEVGVGQGEVVEGTPMKEAVE--------VRLVTIPSTGEPRGLTEGVRQIYNEHNYCRLVMS
XM_007474452 Monodelphis domestica              VTQLKVFTPKSRSLLKRSPKSRPSGFNFHVSNRPGWLKVSPLTRYAPGLSPTQSTPAGAELVPEPSQTLRPALYKPEPR------EKL--PQPPSEVGVGQGEAVEGTVTKEAVE--------VRLVTIPSTGEPRGLTEGVRQIYNEHNYCRLVMS
XM_004464052 Dasypus novemcinctus               VTQLKVFTPKSHSPLKRSPKYRPSGFNFHVSNHPGWLKVSPLTKYAPGLSPSRTPPAGVGLVPGPLQTLKPVLCRPEPS------ERR--PPLPSEGGVARVEVAAGPPMREVAE-AAAVVMVVRPVITPSPGESQTSLESVRQIYSEHNYCRLIL-
XM_004585717 Ochotona princeps                  VTQLQVVTPRSHSPLKRSPKSRPSGFNFHASNHPGWLKVSPLTRYAPGSSLSRGPPAGAGLAPGPSQTLKPVPARPQGREATTAIERR--PPLPSEGGVARVEVAEGPPMREAAE-TAAVVMVVRPVASLAPAEPQAALESVRQIYSEHNYCRLVL-
XM_016187970 Erinaceus europaeus                VTQLKVFTPKSHSPPKRSPKSRPSGFNFHVSNHPGWLKVSPLTRYVPASSPSQSPPAGVGPMPGPSQTLKPVLCRSEGQEATTAIERQ--PPLPSEGGVARVEVAAGPRMREVAD-TATVVMVVRPVATVSPGEAQTPLESVRQIYSEHNYCRLVL-
XM_008834843 Nannospalax galili                 VTQLKVFTPKSHSPLKRSPKSRPSGFNFHVSNHPGWLKVGPLTRYAPASSQSRSPPAGVGLVPGPLQTLKPVLCRPEGREATTAIEKR--LPLPSEGGVARVEVAAGPSMREVAE-ATAVVMVVRPVVTLSPGEPRAPLETVRQIYNEHNYCRFVL-
XM_012951056 Jaculus jaculus                    VTQLKVFTPKSHSPLKRSPKSRPSGFNFHVSNHPGWLKVSPLTRYVPGLSPSRSPPAGAGLVPGPSQTLKPVLCRPEGQEATTAIERQ--PPLPSEGGVARVEVAAGPPMREVVE---AVVMVVRPVATLNPGESQAPLERVRQIYSEHNYCRLVL-
XM_006881688 Elephantulus edwardii              VTQLKVFTPKSHSPLKKSPKSRPSGFNFHASNHPGWLKVSPLTRYAPGSSPTRNPPAGAGLAPGPLQTLKPVLCRPEPS------ERR--PPLPSEGGVARVEVAAGPPMREVAE-ATTVVMVVRPVATLSPGEPRVPLESVRQIYSEHNYCRLVL-
XM_006860770 Chrysochloris asiatica             VTQLKVFTPKSPSPLKKSLKSRPSGFNFHASNHPGWLKVSPLTRYAPGSSPARNPPAGAALAPGPSQTLKPVLCRPEPS------ERR--PPLPSEGGVARVEVAAGPPIREVAE-AATVVMVVRPVATLSSGEPRAPLESVRQIYSEHNYCRLVL-
XM_013008731 Echinops telfairi                  VTQLKVFTPKSHSPLKRSPKSRPSGFNFHASNHPGWLKVSPLTRYAPGSSLSQSPPAGVGPVLGPSQTLKPVLCRPEPS------ERR--PPLPSEGGVARVEVAAGPLMREVAE-AAALVMVVRPVATLSPGEPRAPLENARQIYSEHNYCRLVL-
XM_007934547 Orycteropus afer afer              VTQLKVFTPKSHSPLKRSPKSRPSGFNFHASNHPGWLKVSPLTRYAPGSSPSRNPPTGAELAPGPSQTLKPVLCRPEPS------ERR--PPLPSEGGVARVEVAAGPPMREAAE-AAAVVMVVRPVATLSPGEPRAPLESVRQIYSEHNYSRLVL-
XM_004370455 Trichechus manatus latirostris     VTQLKVFTPKSHSPLKRSPESRPSGFNFHVSNHPGWLKVSPLTRYAPGSSPIRNLPAGAGLAPGPSQTLKPVLCRPEPS------ERR--PPLPSEGGVARVEVAAGPPMKEVAE-AAAVVMVVRPVATLSPGEPRAPLESVRQIYSEHNYCRLVL-
XM_010591603 Loxodonta africana                 VTQLKVFTPKSHSPLKRSPESRPSGFNFHVSNHPGWLKVSPLTKYAPASSPSRIPPVGAGLVPGPSQTLKPVLCRPEPS------ERR--PPLPSEGGVARVEVAAGPPMREVAE-AAAVVMVVRPVATLSPGEPRAPLESVRQIYSEHNYCRLVL-
XM_012933645 Sorex araneus                      VTQLKVFTPKSHSPPKRSPKSRPSGFNFHVSNHPGWLKVSPLTRYAPELSPSQSLPAGAGQGPGPSQTLKPVLCRSEGREATTA-ERR--PPLPSEGGVARVEVAAGPPTREVAE--TAVVMVVRPVATLSLGEARAPLESVRQIYSEHNYCRLVL-
XM_008256142 Oryctolagus cuniculus              VTQLKVFTPKSHSPLKRSPKSRPSGFNFHASNHPGWLKVSPLTRYAPGSSLPRSPPAGAGLAPGPSQTLKPVLGRPEGREATTAIERR--PPLPSEGGVARVEVAAGPPMREVAE-AAAVVMVVRPVATLNPREPQAPLESVRQIYSEHNYCRLVL-
XM_008074492 Carlito syrichta                   VTQLKVFTPKSHSPLKRSPKSHPSGFNFHVSNHPGWLKVSPLTRYAPGSSPSRSPPAGAGLVPGPSQTLKPVLCRSEGREATTAIERR--PPRPAEAGGARVEVAAGPSMREVAE-ATAVVMVVRPVATLSPGEPRAPLESIRQIYSEHNYCRLVL-
XM_014590207 Tupaia chinensis                   VTQLKVFTPKSHSPLKRSPKSRPSGFNFHVSNHPGWLKVSPLTRYAPGSSPSRSPPAGDGLVPGPSQTLKPVLCRSEGREATTAIERR--PPLPSGGGVAGVEVAAGPPMREVAE-TAAVVMVVRPVTTLSAGEPQAPLESVRQIYSEHNYCRLVL-
XM_013010673 Dipodomys ordii                    VTQLKVFTPKSHSPLKRSPKSRPSGFNFHVSNHPGWLKVSPLTRYAPGSSPSRTPPAGAGLVPGPSQTLKPVLCRPEGREATTAIERR--PPLPSEGGVARVEVATGPLMREVAE-AATVVMLVRPVATLSPGEPQAPLERVRQIYSEHNYCRLVL-
XM_005086071 Mesocricetus auratus               VTQLKVFTPKSHSPLKRSPKSRPSGFNFHVSNHPGWLKVGPLTRYAPGSSPSRSPPAGVGLVPGPSQTLKPVLCRPEGQEATTAIERR--PPLPSEGGVARVEVAVGPSMREVAE-AAAVVMVVRPVATLSPGEPPVPLERVRQIYSEHNYCRLVL-
XM_016975522 Cricetulus griseus                 VTQLKVFTPKSHSPQKRSPKSRPSGFNFHVSNHPGWLKVGPLTRYAPGSSPSQSPPAGVGLVPGPSQTLKPVLCRPEGREATTAIERR--PPLPSEGGVARVEVAVGPSMREVAE-AAAVVMVVRPLATLSPGEPQVPLERVRQIYSEHNYCRLVL-
XM_006985536 Peromyscus maniculatus bairdii     VTQLKVFTPKSHSPLKRSPKSRPSGFNFHVSNHPGWLKVGPLTRYAPGLSPSRSPPAGVGLVPGPSQTLKPVLCRPEGREATTAIERR--QPLPSEGGVARVEVAVGPSMREVAE-AAAVVMVVRPVATLSPGEPQAPLERVRQIYSEHNYCRLVL-
NM_001039939 Mus musculus                       VTQLKVFTPKSHSPLKRSPKSRPSGFNFHVSNHPGWLKVGPLTRYAPGSSPSRSPPAGVGLVPGPSQTLKPVLCRPEGREVTTAIERR--PPLPSEGGVARVEVAVGPSMREVAE-TAAVVMVVRPVATLSPGEPQAPLERVRQIYSEHNYCRLVL-
XM_005363226 Microtus ochrogaster               VTQLKVFTPKSHSPLKRSPKSRPSGFNFHVSNHPGWLKVGPLTRYAPGSSPSQSPPAGVGLVPGPLQTLKPVLCRPEGREATTAIERR--PPLPSEGGVARVEVAVGPSMREVAE-AAAVVMVVRPVTTLSPGEPQAPLERVRQIYSEHNYCRLVL-
XM_004840576 Heterocephalus glaber              VTQLKVFTPKSHSPLKRSPKSHPSGFNFHVSNHPGWLKVSPLTRYAPGSSPSRSPPAGAGLVPEPLQTLKPVLCRPEGQEATTVIERR--PPLPSEGGVARVEVAAGPPMREVAE-AAAVVMVVRPVVTLSPGEPRAPPESVRQIYSEHNYCRLVL-
XM_010628451 Fukomys damarensis                 VTQLKVFTPKSHSPLKRSPKSRPSGFNFHVSNHPGWLKVSPLTRYAPGSSPSQSPPAGAGLVPGPLQTLKPVLCSPEGREATTVIERR--PPLPSEGGVARVEVAAGPPMREVAE-AAAVVMVVRPVASLSPGEPRAPPESVHQIYSEHNYCRLVL-
XM_005384868 Chinchilla lanigera                VTQLKVFTPKSHSPLKRSPKSRPSGFNFHVSNHPGWLKVSPLTRYAPGSSPSQSPLAGAGLLPGPSQTLKPVLCRPEGREATTAMERR--PPLLSEGGVARVEVAAGPPMREVAE-AAAVVMVVRPVATLSPGESRAPPESVRQIYSEHNYCRLVL-
XM_013159847 Cavia porcellus                    VTQLKVFTPKSHSPLKRSPKSRPSGFNFHVSNHPGWLKVSPLTRYAPGSSLSQSPPAGAGLVPGPSQTLKPVLCRPEGREATTAIERR--PPLPSEGGVARVEVAAGPPMREVAE-AAAVVMVVRPVATLSPGEPRAPPENVRQIYSEHNYCRLVL-
XM_015481723 Marmota marmota marmota            VTQLKVFTPKSHSPLKRSPKSRPSGFNFHVSNHPGWLKVSPLTRYVPGSSPSQSLPTGAGLVPGPSQTLKPVLCRPEGQEATTAIERR--PPLPSEVGVAQVEVAAGPPMREVAE-AAAVVMVVRPVATLSPGEPRAPLESVRQIYSEHNYCRLVL-
XM_013356440 Ictidomys tridecemlineatus         VTQLKVFTPKSHSPLKRSPKSRPSGFNFHVSNHPGWLKVSPLTRYVPGSSPSQSLPTGAGLVPGPSQTLKPVLCRPEGREATTAIERR--PPLPSEVGVARVEVAAGPPMREVAE-AAAVVMVVRPVATLSPGEPRAPLESVRQIYSEHNYCRLVL-
XM_012804162 Otolemur garnettii                 VTQLKVFTPKSHSPLKRSPKSRPSGFNFHVSNHPGWLKVSPLTRYAPGSSPSRSPPTGAGLAPGPSQTLKPVLCKSEGREATNAIERR--PPLPSEGGVARVEVAAGPPMREVAEAAAAVVMVVRPVATLSQGEPRAPLESVRQIYSEHNYCRLVL-
XM_012754776 Microcebus murinus                 VTQLKVFTPKSHSPLKRSPKSRPSGFNFHVSNHPGWLKVSPLTRYAPGSSPSRSPPAGAGLAPGPSQTLKPVLCKPEGREVTTAIERR--PPLPSEGGVARVEVAAGPPMREVAEAAAAVVMVVRPVATLSPGEPRAPLERVRQIYSEHNYCRLVL-
XM_012637708 Propithecus coquereli              VTQLKVFTPRSHSPLKRSPKSRPSGFNFHVSNHPGWLKVSPLTRYAPGSSPSRSPPAGAGLAPGPSQTLKPVLCKPEGREVTTAIERR--PLLPSEGGVARVEVAAGPPMREVAEAAAAVVMVVRPVATLSPGEPRAPLESVRQIYSEHNYCRLVL-
XM_012455205 Aotus nancymaae                    VTQLKVFTPKSHSPLKRSPKSRPSGFNFHVSNHPGWLKVSPLTRYAPGSSPSRSPPAGAGPVPGPSQTLKPVLCRSEGREVTTAIERR--PPLPSEGGVARVEVAAGPPMREAVE-AAAVVMVVRPVATLSPGEARAPLESVRQIYSEHNYCRLVL-
XM_017971854 Callithrix jacchus                 VTQLKVFTPKSHSPLKRSPKSRPSGFNFHVSNHPGWLKVSPLTRYAPGSSPSRSPPAGAGPVPGPSQTLKPVLCRSEGREVTTAIERR--PPLPSEGGVARVEVAAGPPMREAVE-AAAVVMVVRPVATLIPGEARAPLESVRQIYSEHNYCRLVL-
XM_017514550 Cebus capucinus imitator           VTQLKVFTPKSHSPLKRSPKSRPSGFNFHVSNHPGWLKVSPLTRYAPGSSPSRSPPAGAGPAPGPSQTLKPVLCRSEGREVTTAIERR--PPLPSEGGVARVEVAAGPPMREAVE-AAAVVMVVRPVATLSPGEARAPLESVRQIYSEHNYCRLGL-
XM_003932071 Saimiri boliviensis boliviensis    VTQLKVFTPKSRSPLKRSPKSRPSGFNFHVSNHPGWLKVSPLTRYAPGSSPSRSPPAGAGPAPGPSQTLKPVLCRSEGREVTTAIERR--PPLPSEGGVARVEVAAGPPMREAVE-AAAVVMVVRPVATLSPGEARAPLESVRQIYSEHNYCRLIL-
XM_012052496 Cercocebus atys                    VTQLKVFTPKSHSPLKRSPKSHPSGFNFHVSNHPGWLKVSPLTRYAPGSSPPRSPPAGAGLAPGPSQTLKPVLCRSEGREVMTAIERR--PPLPSEGGVARVEVAAGPSMREVAE-AAAVVMVVRPVATLSPGEARAPLESVRQIYSEHNYCRLIL-
XM_008020495 Chlorocebus sabaeus                VTQLKVFTPKSHSPLKRSPKSRPSGFSFHVSNHPGWLKVSPLTRYAPGSSPPRSPPAGAGLAPGPSQTLKPVLCRSEGREVMTAIERR--PPLPSEGGVARVEVAAGPPMREVAE-AAAVVMVVRPVATLSPGEARAPLESVRQIYSEHNYCRLIL-
XM_005568667 Macaca fascicularis                VTQLKVFTPKSHSPLKRSPKSRPSGFNFHVSNHPGWLKVSPLTRYAPGSSPPRSPPAGAGLAPGPSQTLKPVLCRSEGREVMTAIERR--PPLPSEGGVARVEVAAGPPMREVAE-AAAVVMVVRPVATLSPGEARAPLESVRQIYSEHNYCRLIL-
XM_011979776 Mandrillus leucophaeus             VTQLKVFTPKSHSPLKRSPKSRPSGFNFHVSNHPGWLKVSPLTRYAPGSSPPRSPPAGAGLAPGPSQTLKPVLCRSEGREVMTAIERR--PPLPSEGGVARVEVAAGPPMREVAE-AAAVVMVVRPVATLSPGEARAPLESVRQIYSEHNYCRLIL-
XM_009216344 Papio anubis                       VTQLKVFTPKSHSPLKRSPKSRPSGFNFHVSNHPGWLKVSPLTRYAPGSSPPRSPPAGAGLAPGPSQTLKPVLCRSEGREVMTAIERR--PPLPSEGGVARVEVAAGPPMREVAE-AAAVVMVVRPVATLSPGEARAPLESVRQIYSEHNYCRLIL-
XM_015149251 Macaca mulatta                     VTQLKVFTPKSHSPLKRSPKSRPSGFNFHVSNHPGWLKVSPLTRYAPGSSPPRSPPAGAGLAPGPSQTLKPVLCRSEGREVMTAIERR--PPLPSEGGVARVEVAAGPPMREVAE-AAAVVMVVRPVATLSPGEARAPLESVRQIYSEHNYCRLIL-
XM_011766345 Macaca nemestrina                  VTQLKVFTPKSHSPLKRSPKSRPSGFNFHVSNHPGWLKVSPLTRYAPGSSPPRSPPAGAGLAPGPSQTLKPVLCRSEGREVMTAIERR--PPLPSEGGVARVEVAAGPPMREVAE-AAAVVMVVRPVATLSPGEARAPLESVRQIYSEHNYCRLIL-
XM_016937663 Pan troglodytes                    VTQLKVFTPKSHSPLKRSPKSRPSGFNFHVSNHPGWLKVSPLTRYAPGSSPPRSPPAGVGLAPGPSQTLKPVLCRSEGREVTTAIERR--PPLPSEGGVARVEVAAGPPMREVAE-AAAVVMVVRPVATLSPGEARAPLESVRQIYSEHNYCRLIL-
NM_015338    Homo sapiens                       VTQLKVFTPKSHSPLKRSPKSRPSGFNFHVSNHPGWLKVSPLTRYAPGSSPPRSPPAGVGLAPGPSQTLKPVLCRSEGREVTTAIERR--PPLPSEGGVARVEVAAGPPMREVAE-AAAVVMVVRPVATLSPGEARAPLESVRQIYSEHNYCRLIL-
XM_003814739 Pan paniscus                       VTQLKVFTPKSHSPLKRSPKSRPSGFNFHVSNHPGWLKVSPLTRYAPGSSPPRSPPAGVGLAPGPSQTLKPVLCRSEGREVTTAIERR--PPLPSEGGVARVEVAAGPPMREVAE-AAAVVMVVRPVATLSPGEARAPLESVRQIYSEHNYCRLIL-
XM_004061980 Gorilla gorilla gorilla            VTQLKVFTPKSHSPLKRSPKSRPSGFNFHVSNHPGWLKVSPLTRYAPGSSPPRSPPAGVGLAPGPSQTLKPVLCRSEGREVTTAIERR--PPLPSEGGVARVEVAAGPPMREVAE-AAAVVMVVRPVATLSPGEARAPLESVRQIYSEHNYCRLIL-
XM_003273514 Nomascus leucogenys                VTQLKVFTPKSHSPLKRSPKSRPSGFNFHVSNHPGWLKVSPLTRYAPGSSPPRSPPAGVGLAPGPSQTLKPVLCRSEGQEVTTAIERR--PPLPSEGGVARVEVAAGPPMREVAE-AAAVVMVVRPVATLSPGEARAPMESVRQIYSEHNYCRLIL-
XM_011959875 Colobus angolensis palliatus       VTQLKVFTPKSHSPLKRSPKSRPSGFNFHVSNHPGWLKVSPLTRYAPGSSPPRSPPAGAGLAPGPSQTLKPVLCRSEGQEVTTAIERR--PPLPSEGGVARVEVAAGPPMREVAE-AAAVVMVVRPVATSSPGEARAPLESVRQIYSEHNYCRLIL-
XM_017884118 Rhinopithecus bieti                VTQLKVFTPKSHSPLKRSPKSRPSGFNFHVSNHPGWLKVSPLTRYAPGSSPPRSPPAGAGLAPGPSQTLKPVLCRSEGQEVTTAIERR--PPLPSEGGVARVEVAAGPPMREVAE-AAAVVMVVRPVATLSPGEARAPLESVRQIYSEHNYCRLIL-
XM_010352765 Rhinopithecus roxellana            VTQLKVFTPKSHSPLKRSPKSRPSGFNFHVSNHPGWLKVSPLTRYAPGSSPPRSPPAGAGLAPGPSQTLKPVLCRSEGQEVTTAIERR--PPLPSEGGVARVEVAAGPPMREVAE-AAAVVMVVRPVATLSPGEARAPLESVRQIYSEHNYCRLIL-
XM_014554657 Camelus ferus                      VTQLKVFTPKSHSPPKRSPKSRPSGFNFHVSNHPGWLKVSPLTRYAPASSPSRSPPAGAGLAPGPLQTLKPVLCRSEGREATTAIRKAAXXXXXXXGGVARVEVAAGPPMREVAE-TAAVVMVVRPVATLSPGEPRAPLESVRQIYSEHNYCRLVL-
XM_008577080 Galeopterus variegatus             VTQLKVFTPKSHSPLKRSPKSRPSGFNFHVSNHPGWLKVSPLTRYAPGSSPSRSPPAGAGLVPGPSQTLKPVLCKSEGREAPTAIERR--PQLPSEGGVARVEVAAGPPMREVAE-MAAVVMVVRPVATLSAGEPRAPLESMRQIYSEHNYCRLVF-
XM_004687292 Condylura cristata                 VTQLKVFTPKSHSPPKRSPKSRPSGFNFHVSNHPGWLKVSPLTRFAPASSPSRSPPAGAGLVPGPSQTLKPVLCRSEGPEATTAIERR--PPLPSEAGVARVEVAAGPPMREVAE-AAAVVMVVRPMATLSPGDPRAPLENVRQIYSEHNYCRLVL-
XM_004442480 Ceratotherium simum simum          VTQLKVFTPKSHSPPKRSPKSRPSGFNFHVSNHPGWLKVSPLTRYAPASSPSRSPPAGAGLVPGPSRTLKPVLCRSEGREATTAIERR--LPLPSEAGVARVEVAAGPPMREVAE-AAPVVMVVRPVATLSPEETRGPLESVRQIYSEHNYCRLVL-
XM_008140954 Eptesicus fuscus                   VTQLKVFTPKSHSPPKRSPKSRPSGYNFHVSNHPGWLKVSPLTRYAPELSPSRSPPAGAGLVPGPSQTLKPVLCRSEGREATTAIERR--PPLPSEGGVARVEVAAGPPMREVAE-AAAVVMVVRPVATLSPEEPRAPLESVRQIYSEHNYCRLVL-
XM_014550680 Myotis brandtii                    VTQLKVFTPKSHSPPKRSPKSRPSGYNFHVSNHPGWLKVSPLTRYAPELSPSRSPPAGAGLVPGPSQTLKPVLCRSEGREATTAIERR--PPLPSEGGVARVEVAAGPPMREVAE-AAAVVMVVRPVATLSPEEPRAPLESVRQIYSEHNYCRLVL-
XM_015566642 Myotis davidii                     VTQLKVFTPKSHSPPKRSPKSRPSGYNFHVSNHPGWLKVSPLTRYAPELSPSRSPPAGAGLVPGPSQTLKPVLCRSEGREATTAIERR--PPLPSEGGVARVEVAAGPPMREVAE-AAAVVMVVRPVATLSPEEPRAPLESVRQIYSEHNYCRLVL-
XM_014456449 Myotis lucifugus                   VTQLKVFTPKSHSPPKRSPKSRPSGYNFHVSNHPGWLKVSPLTRYAPELSPSRSPPAGAGLVPGPSQTLKPVLCRSEGREATTVIERR--PPLPSEGGVARVEVAAGPPMREVAE-AAAVVMVVRPVATLSPEEPRAPLESVRQIYSEHNYCRLVL-
XM_013053414 Mustela putorius furo              VTQLKVFTPKSHSPPKRSPKSRPSGFNFHVSNHPGWLKVSPLTRFAPASSPSRSPPAGAGLVPGPSQTLKPVLCKSEGREATTVIERR--PPLPSEGGVARVEVAAGPPMREVAE-AAAVVMVVRPVPTLSPAEPRVPLESVRQIYSEHNYCRLVL-
XM_015537063 Panthera tigris altaica            VTQLKVFTPRSHSPPKRSPKSRPSGFNFHVSNHPGWLKVSPLTRYAPASSPSRSPPAGAGLVPGPSQTLKPVLCKSEGREATTVIERR--APLPSGGGGGRVEVAAGPPMREVAE-AAAVVMVVRPVATLSPAEPRAPLESVRQIYSEHNYCRLVL-
XM_006740976 Leptonychotes weddellii            VTQLKVFTPKSHSPPKRSPKSRPSGFNFHVSNHPGWLKVSQLTRYVPASSPSRSPPAGAGLVPGPSQTLKPVLCKSEGREATTVIERR--PPLPSEGGVARVEVATGPPMREVAE-AAAVVMVVRPVATLSPAEPRAPLESVRQIYSEHNYCRLVL-
XM_004393041 Odobenus rosmarus divergens        VTQLKVFTPKSHSPPKRSPKSRPSGFNFHVSNHPGWLKVSPLTRYVPASSPSRSPPAGAGLVPGPSQTLKPVLCKSEGREATTVIERR--PPLPSEGGVARVEVATGPPMREVAE-AAAVVMVVRPVATLSPAEPRAPLESVRQIYSEHNYCRLVL-
XM_015071869 Acinonyx jubatus                   VTQLKVFTPRSHSPPKRSPKSRPSGFNFHVSNHPGWLKVSPLTRYAPASSPSRSPPAGAGLVPGPSQTLKPVLCKSEGREATTVIERR--PPLPSEGGVARVEVAAGPGMREVAE-AAAVVVVVRPVATLSPVEPRAPLESVRQIYSEHNYCRLVL-
XM_011223720 Ailuropoda melanoleuca             VTQLKVFTPKSHSPPKRSPKSRPSGFNFHVSNHPGWLKVSPLTRYAPASSPSRSPPAGAGLVPGPSQTLKPMLCKSEGREATTAIERR--PPLPSEGGVARVEVAAGPPMREVAE-AAAVVMVVRPVATLSPAEPRAPLESVRQIYSEHNYCRLVL-
XM_019458810 Panthera pardus                    VTQLKVFTPRSHSPPKRSPKSRPSGFNFHVSNHPGWLKVSPLTRYAPASSPSRSPPAGAGLVPGPSQTLKPVLCKSEGREATTVIERR--PPLPSEGGVARVEVAAGPPMREVAE-AAAVVMVVRPVATLSPAEPRAPLESVRQIYSEHNYCRLVL-
XM_019826661 Felis catus                        VTQLKVFTPRSHSPPKRSPKSRPSGFNFHVSNHPGWLKVSPLTRYAPASSPSRSPPAGAGLVPGPSQTLKPVLCKSEGREATTVIERR--PPLPSEGGVARVEVAAGPPMREVAE-AAAVVMVVRPVATLSPAEPRAPLESVRQIYSEHNYCRLVL-
XM_005634922 Canis lupus familiaris             VTQLKVFTPKSHSPPKRSPKSRPSGFNFHVSNHPGWLKVSPLTRYAPASSPSRSPPAGAGLVPGPSQTLKPVLCKSEGREATTVIERR--PPLPSEGGVARVEVAAGPPMREVAE-AAAVVMVVRPVATLSPVEPRTPLESVRQIYSEHNYCRLVL-
XM_008703387 Ursus maritimus                    VTQLKVFTPKSHSPPKRSPKSRPSGFNFHVSNHPGWLKVSPLTRYAPASSPSRSPPAGAGLVPGPSQTLKPVLCKSEGREATTVIERR--PPLPSEGGVARVEVAAGPPMREVAE-AAAVVMVVRPVATLSPVEPRAPLESVRQIYSEHNYCRLVL-
XM_005672837 Sus scrofa                         VTQLKVFTPKSHSPPKRSPKSRPSGFSFHVSNHPGWLKVSPLTRYAPASSPSRSPPAGAGLAPGPLQTLKPVLCKSEGREATTAIERR--PPLPSEGGVARVEVAAGPPMREVAE-AAAVVMVVRPVATLSPGEPRVPLECVRQIYSEHNYCRLIL-
XM_014845061 Equus asinus                       VTQLKVFTPKSHSPPKRSPKSRPSGFNFHVSNHPGWLKVSPLTRYAPASSPSRSPPAGAGLAPGPSQTLKPVLCRSEGREATTAIERR--LPLPSEGGVARVEVAAGPPMREVAE-AAAVVMVVRPVATLSPEEPQALLESVRQIYSEHNYCRLVL-
```

```
XM_005604562 Equus caballus                       VTQLKVFTPKSHSPPKRSPKSRPSGFNFHVSNHPGWLKVSPLTRYAPASSPSRSPPAGAGLAPGPSQTLKPVLCRSEGREATTAIERR--LPLPSEGGVARVEVAAGPPMREVAE-AAAVVMVVRPVATLSPEEPQALLESVRQIYSEHNYCRLVL-
XM_008525258 Equus przewalskii                    VTQLKVFTPKSHSPPKRSPKSRPSGFNFHVSNHPGWLKVSPLTRYAPASSPSRSPPAGAGLAPGPSQTLKPVLCRSEGREATTAIERR--LPLPSEGGVARVEVAAGPPMREVAE-AAAVVMVVRPVATLSPEEPQALLESVRQIYSEHNYCRLVL-
XM_005887939 Bos mutus                            VTQLKVFTPKSHSPPKRSPKSRPSGFNFHVSNHPGWLKVSPLTRYVPASSPSRSPPAGAGLVPGPLQTLKPVLCRSEGREATPAXXXX--PPLPSEVGVARVEVAAGPPMREVAE-AAAVVMVVRPVATLSPGGPRVPLESVRQIYSEHNYCRLVL-
XM_019748488 Rhinolophus sinicus                  VTQLKVFTPKSHSPPKRNPKSRPSGFNFHVSNHPGWLKVSPLTRYVPASSPSRSPPAAAGLAPGPLQTLKPVLCRSEGREATTAIERR--PPLPSEGGVARVEVAAGPPMREVAE-AAAVVMVVRPVATLSPGEPRAPLESVRQIYSEHNYCRFIL-
XM_016200632 Miniopterus natalensis               VTQLKVFTPKSHSPPKRSPKSRPSGYNFHVSNHPGWLKVSPLTRYAPALSPSRSPPAGAGLAPGPSQTLKPVLCRSEGREATTAMERR--PPLPSEGGVARVEVAAESPMREVAE--AAVVMVVRPVATLSPGEPRAPLESVRQIYSEHNYCRLVL-
XM_016148805 Rousettus aegyptiacus                VTQLKVFTPKSHSPPKRSPKSRPSGFNFHVSNHPGWLKVSPLTRYAPASSPSRSPPAGAGLAPGHLQTLKPVLCRSEGREATTAIERR--PPLPSEGGVARVEVTAGPPMREVAE-AAALVMVVRPVATLSPGEPRAPLESVRQIYSEHNYCRLIL-
XM_011380179 Pteropus vampyrus                    VTQLKVFTPKSHSPPKRSPKSRPSGFNFHVSNHPGWLKVSPLTRYAPASSPSRSPPAGPGLAPGHLQTLKPVLCRSEGREATTAIERR--PPLPSEGGVARVEVTAGPPMREVAE-AAALVMVVRPVATLSPGEPRAPLETARQIYSEHNYCRLIL-
XM_006922041 Pteropus alecto                      VTQLKVFTPKSHSPPKRSPKSRPSGFNFHVSNHPGWLKVSPLTRYAPASSPSRSPPAGPGLAPGHLQTLKPVLCRSEGREATTAIERR--PPLPSEGGVARVEVTAGPPMREVAE-AAALVMVVRPVATLSPGEPRAPLESARQIYSEHNYCRLIL-
XM_006202660 Vicugna pacos                        VTQLKVFTPKSHSPPKRSPKSRPSGFNFHVSNHPGWLKVSPLTRYAPASSPSRSPPAGAGLAPGPLQTLKPVLCRSEGREATTAIERR--PPLPSEGGVARVEVAAGPPMREVAE-TAAVVMVVRPVATLSPGEPRAPLESVRQIYSEHNYCRLVL-
XM_010960874 Camelus bactrianus                   VTQLKVFTPKSHSPPKRSPKSRPSGFNFHVSNHPGWLKVSPLTRYAPASSPSRSPPAGAGLAPGPLQTLKPVLCRSEGREATTAIERR--PPLPSEVGVARVEVAAGPPMREVAE-TAAVVMVVRPVATLSPGEPRAPLESVRQIYSEHNYCRLVL-
XM_010993907 Camelus dromedarius                  VTQLKVFTPKSHSPPKRSPKSRPSGFNFHVSNHPGWLKVSPLTRYAPASSPSRSPPAGAGLAPGPLQTLKPVLCRSEGREATTAIERR--PPLPS------------------------------PVATLSPGEPRAPLESVRQIYSEHNYCRLVL-
XM_007122347 Physeter catodon                     VTQLKVFTPKSHSPPKRSPKSRPSGFNFHVSNHPGWLKVSPLTRYAPASSPSRSPPAGAGLAPGPLQTLKPVLCRSEGREATTAIERR--PPLPSEGGVARVEVAAGPPMREVAE-ASAVVMVVRPVATLIPGEPQVPLESVRQIYSEHNYCRLVL-
XM_007446853 Lipotes vexillifer                   VTQLKVFTPKSHSPPKRSPKSRPSGFNFHVSNHPGWLKVSPLTRYAPASSPSRSPPAGAGLAPGPLQTLKPVLCRSEGREATTAIERR--PPLPSEGGVARVEVAAGPAMREVAE-ASAVVMVVRPVATLSPGEPQVPLESVRQIYSEHNYCRLVL-
XM_004272710 Orcinus orca                         VTQLKVFTPKSHSPPKRSPKSRPSGFNFHVSNHPGWLKVSPLTRYAPASSPSQSPPAGAGLAPGPLQTLKPVLCRSEGREATTAIERR--PPLPSEGGVARVEVAAGPPMREVAE-ASAVVMVVRPVATLSPGEPQVPLESVRQIYSEHNYCRLVL-
XM_007193384 Balaenoptera acutorostrata scammoni  VTQLKVFTPKSHSPPKRSPKSRPSGFNFHVSNHPGWLKVSPLTRYAPASSPSRSPPAGAGLAPGPLQTLKPVLCRSEGREATTAIERR--PPLPSEGGVARVEVAAGPPMREVAE-ASAVVMVVRPVATLSPGEPQVPLESVRQIYSEHNYCRLVL-
XM_019950775 Tursiops truncatus                   VTQLKVFTPKSHSPPKRSPKSRPSGFNFHVSNHPGWLKVSPLTRYAPASSPSRSPPAGAGLAPGPLQTLKPVLCRSEGREATTAIERR--PPLPSEGGVARVEVAAGPPMREVAE-ASAVVMVVRPVATLSPGEPQVPLESVRQIYSEHNYCRLVL-
XM_017648617 Manis javanica                       VTQLKVFTPKSHSPPKRSPKSRPSGFNFHVSNHPGWLKVSPLTRYAPASSPSRSPPAGAGLVPGPSQTLKPVLCRSEGREATTAIERR--PPLPSEGGVARVEVAAGPPMREVAE-AAAVVMVVRPVATLSPGEPRVPLESVRQIYSEHNYCRLVF-
XM_019645788 Hipposideros armiger                 VTQLKVFTPKSHSPPKRSPKSRPSGFNFHVSNHPGWLKVSPLTRYVPASSPSRSPPAGAGLVPGPLQTLKPVLCRSEGREATTAIERR--PPLPSEGGVARVEVAAGPPMREVAE-AAAVVMVVRPVATLSPGESRAPLESVRQIYSEHNYCRLIL-
XM_006064407 Bubalus bubalis                      VTQLKVFTPKSHSPPKRSPKSRPSGFNFHVSNHPGWLKVSPLTRYVPASSPSRSPPAGAGLVPGPLQTLKPVLCRSEGRAATTAVERR--PPLPSEVGVARVEVAAGPPMREVAE-AAAVVMVVRPVATLSPGEPRVPLESVRQIYSEHNYCRLVL-
XM_600364    Bos taurus                           VTQLKVFTPKSHSPPKRSPKSRPSGFNFHVSNHPGWLKVSPLTRYVPASSPSRSPPAGAGLVPGPLQTLKPVLCRSEGREATTAIERR--PPLPSEVGVARVEVAAGPPMREVAE-AAAVVMVVRPVATLSPGGPRVPLESVRQIYSEHNYCRLVL-
XM_019972246 Bos indicus                          VTQLKVFTPKSHSPPKRSPKSRPSGFNFHVSNHPGWLKVSPLTRYVPASSPSRSPPAGAGLVPGPLQTLKPVLCRSEGREATTAIERR--PPLPSEVGVARVEVAAGPPMREVAE-AAAVVMVVRPVATLSPGGPRVPLESVRQIYSEHNYCRLVL-
XM_010841718 Bison bison bison                    VTQLKVFTPKSHSPPKRSPKSRPSGFNFHVSNHPGWLKVSPLTRYVPASSPSRSPPAGAGLVPGPLQTLKPVLCRSEGREATTAIERR--PPLPSEVGVARVEVAAGPPMREVAE-AAAVVMVVRPVAPLSPGGPRVPLESVRQIYSEHNYCRLVL-
XM_005958892 Pantholops hodgsonii                 VTQLKVFTPKSHSPPKRSPKSRPSGFNFHVSNHPGWLKVSPLTRYVPASSPSRSPPAGAGLVPGPLQTLKPVLCRSEGREATTAIERR--PPPP------RVEVAAGPPMREVAE-AAAVVMVVRPVATLSPGEPRVPLESVRQIYSEHNYCRLVL-
XM_018057698 Capra hircus                         VTQLKVFTPKSHSPPKRSPKSRPSGFNFHVSNHPGWLKVSPLTRYVPASSPSRSPPAGAGLVPGPLQTLKPVLCRSEGREATTAIERR--PPLPSEVGVARVEVAAGPPMREVAE-AAAVVMVVRPVATLSPGEPRVPLETVRQIYSEHNYCRLVL-
XM_012151672 Ovis aries musimon                   VTQLKVFTPKSHSPPKRSPKSRPSGFNFHVSNHPGWLKVSPLTRYVPASSPSRSPPAGAGLVPGPLQTLKPVLCRSEGREATTAIERR--PPLPSEVGVARVEVAAGPPMREVAE-AAAVVMVVRPVATLSPGEPRVPLESVRQIYSEHNYCRLVL-
XM_015099825 Ovis aries                           VTQLKVFTPKSHSPPKRSPKSRPSGFNFHVSNHPGWLKVSPLTRYVPASSPSRSPPAGAGLVPGPLQTLKPVLCRSEGREATTAIERR--PPLPSEVGVARVEVAAGPPMREVAE-AAAVVMVVRPVATLSPGEPRVPLESVRQIYSEHNYCRLVL-
```

sauropsids

```
XM_008497766 Calypte anna                         VTQLKVFTQKSHSLLKRSQKSHPSGFNFHVLNHPGWLKVSPLTRYAPESSPTRSPPA-GAGPGPEPSQTSKPELCRPEPSEK--LPQQPSE------EGVAQVEGE--AGMKEVVVE-------------------EEDPAAAPSTGDPREPVGSARQICSEHNYCRPPV
XM_019509967 Gavialis gangeticus                  VTQLKVFTQKSHSLLKRSPKSHPSGFNFHVSNHPGWLKVSQLTRYAPGSSPTRSPPV-GAGQVPEHSQTLKPVLCKPEPSER--LLQ-PLE------VGVGQEEEGGAAQIKE-----------------------EETPVAIQSTGNQRELMESVRQIYNEHNYCRLPL
XM_019539485 Crocodylus porosus                   VTQLKVFTQKSHSLLKRSPKSHPSGFNFHVSNHPGWLKVSQLTRYAPGSSPTRSPLV-GAGQVPEHSQTLKPVLCKPEPSER--LLQ-PLE------AGVGQEEGGVAAQIKE-----------------------EETPVAIQSTGDQRELMESVRQIYNEHNYCRLPL
XM_019500328 Alligator mississippiensis           VTQLKVFTQKSHSLLKRSPKSHPSGFNFHVSNHPGWLKVSQLTRYAPGSSPTRSPLA-GAGQVPEHSQTLKPVLCKPEPSER--LLQ-PLE------AGVGQEEEGGAAQIKE-----------------------EETPVAIQSTGDQRELMESVRQIYNEHNYCRLPL
XM_006117868 Pelodiscus sinensis                  VTQLKVFTQKSHSLLKRSQKSHPSGFNFHVSSHPGWLRVSPLTRYAPGSSPTQSPLV-GAGLVPEHSQTLKLVLCKPEPSER--PLQLPLE------VGVGQEEEG-TAPIKEVVVV-------------------VENPVAIESPGDQRELMEGVRQIYNEHNYCRLSI
XM_007062481 Chelonia mydas                       VTQLKVFTQKSHSLLKRNQKSHPSGFNFHVSNHPGWLRVSPLTRYAPGSSPTRSPLV-GAELVPEHSQTLKLVLCKPEPSER--PLQLPLE------VGVGQEEEG-TAPIKE--VV-------------------EETPVAIQSTGDQRELMEGVRQIYNEHNYCRLSI
XM_008172168 Chrysemys picta bellii               VTQLKVFTQKSHSLLKRNQKSHPSGFNFHVSNHPGWLRVSPLTRYAPGSSPTRNPLV-GAELVPEHSQTLKLVLCKPEPSER--PLQLPLE------VGVGQEEEG-TAPIKE--VV-------------------EENPVAIQSTGDQRELMENVRQIYNEHNYCRLSI
XM_015881880 Coturnix japonica                    VTQLKVFTQKSHSLLKRSQKSHPSGFNFHVLNHPGWLKVSQLTRYATGSSPTRSPPP-GAGAGPELSQTLRPVLCRPEPSEK--LLQPPLV------VGEAPAEAE--TLRGE-----------------------EENPAAIAGVEDQRELDESAHQIYSEHNYSRLSV
XM_010722615 Meleagris gallopavo                  VTQLKVFTQKSHNLLKRSQKSHPSGFNFHVLNHPGWLKVSQLTRYAPGSSPTRSPPP-GAGAGPELSQTLRPVLCRPEPSEK--LLQPPLV------VXAAPAEGE--TL--R--GV-------------------EENAAAIADAGDQRELVESAHQIYSEHNYCRLSV
XM_015296597 Gallus gallus                        VTQLKVFTQKSHNLLKRSQKSHPSGFNFHVLNHPGWLKVSQLTRYAPGSSPTRSPPP-GAGAGPELSQTLRPVLCRPEPSEK--LLQPPLV------VGAAPAEGE--TL--R--GV-------------------EENPAAIAGAGDQRELVESARQIYSEHNYSRLSV
XM_014890102 Sturnus vulgaris                     VTQLKVFTQKSHSLLKRSPKSHPSGFNFHVLNHPGWLKVSPLTRSALGSSPARSPPA-GAAPGPGPSQTLRPVLCKPEPSVK--PPLPPSE------EGAAPAGAE--APMKE--EV-------------------EENPVAVPSTGDPRELMENVRQIYSEHNYSCLFI
XM_018073998 Manacus vitellinus                   VTQLKVFTQKSHSLLKRSPKSHPSGFNFHVLNHPGWLKVSPLTRSAPGSSPARSPPA-GAAPGPGPSQTLRPVLCKPEPSAK--PPPPPSE------EGVAQAGGE--APMKE--EV-------------------EENPAGVPSTGDPRELMESVRQIYNEHNYSRLFL
XM_017822613 Lepidothrix coronata                 VTQLKVFTQKSHSLLKRSPKSHPSGFNFHVLNHPGWLKVSPLTRSAPGSSPARSPPA-GAAPGPGPSQTLRPVLCKPEPSAK--PPPPPSE------EGVAQAGGE--APMKE--EV-------------------EENPAGVPSTGDPRELMESVRQIYNEHNYSRLFL
XM_002192704 Taeniopygia guttata                  VTQLKVFTQKSHSLLKRSPKSHPSGFNFHVLNHPGWLKVSPLTRSAPASSPARSPPA-GAAPGPAPSQTLRPVLCKPEPSAK---PPPPSE------EGAAPAGGE--VPMKE--AV-------------------EENPAAVPSTEDPRELMENVRQIYSEHNYSCLFI
XM_005491248 Zonotrichia albicollis               VTQLKVFTQKSHSLLKRSPKSHPSGFNFHVLNHPGWLKVSPLTRSAPGSSPARSPPA-GAAPGPAPSQTLRPVLCKPEPSAR--PPPPPSE------EGVALVGGE--APRKE--AV-------------------EENPAAAPSTGDPRELMENVRQIYSEHNYSCLFI
XM_016303293 Ficedula albicollis                  VTQLKVFTQKSHSLLKRSPKSHPSGFNFHVLNHPGWLKVSPLTRSAPGSSPARSPPA-GAAPGPAPSQTLRPVLCKPEPSAK--PPPPPSE------EG-APAGGE--APMKE--EV-------------------EESPAAVPSTGDPRELMENVRQIYSEHNYSCLFI
XM_009084380 Acanthisitta chloris                 VTQLKVFTQKSHSLLKRSPKSHPSGFNFHVLNHPGWLKVSPLTRSAPGSSPARSPPA-GAGPGPGPSQTLRPVLCKPEPSAK---PPPPSE------EGVAPEGGE--APMKE--AV-------------------EENPAAVPSTGDPRELMENVRQIYNEHNYSRLFI
XM_005422200 Geospiza fortis                      VTQLKVFTQKSHSLLKRSPKSHPSGFNFHVLNHPGWLKVSPLTRSAPGSSPARSPPA-GAALGPGPSQTLRPVLCKPEPSAK--PPPPPSE------EGVALVGGE--APMKE--AV-------------------EENPAAVPSTGDPRELMENVRQIYSEHNYSCLFI
XM_009093027 Serinus canaria                      VTQLKVFTQKSHSLLKRSPKSHPSGFNFHVLNHPGWLKVSPLTRSAPGSSPARSPPA-GAAPGPGPSQTLRPVLCKPEPSAK--PPPPPSE------EGVALVGGE--APMKE--VV-------------------EENPAAVPSTGDPRELMENVRQIYSEHNYSCLFI
XM_010396950 Corvus cornix cornix                 VTQLKVFTQKSHSLLKRSPKSHPSGFNFHVLNHPGWLKVSPLTRSAPGSSPARSPPA-GAAPGPGPSQTLRPVLCKPEPSAK--PPPPPLE------EGAAPVGGE--APMKE--AV-------------------EENPAAVPSTGDPRELMENVHQIYSEHNYSRLFI
XM_008634138 Corvus brachyrhynchos                VTQLKVFTQKSHSLLKRSPKSHPSGFNFHVLNHPGWLKVSPLTRSAPGSSPARSPPA-GAAPGPGPSQTLRPVLCKPEPSAK--PPPPPLE------EGAAPVGGE--APMKE--AV-------------------EENPAAVPSTGDPRELMENVHQIYSEHNYSRLFI
XM_015647794 Parus major                          VTQLKVFTQKSHSLLKRSPKSHPSGFNFHVLNHPGWLKVSPLTRSAPGSSPARSPPA-GAAPGPGPSQTLRPVLCKPEPSAK--PPPPPSE------EGAAPAGGE--APMKE--AV-------------------EENPAAVPSTGDPRELMENVRQIYSEHNYSRLFI
XM_005524727 Pseudopodoces humilis                VTQLKVFTQKSHSLLKRSPKSHPSGFNFHVLNHPGWLKVSPLTRSAPGSSPARSPPA-GAAPGPGPSQTLRPVLCKPEPSAK--PPPPPSE------EGVAPAGGE--APMKE--VV-------------------EENPAAVPSTGDPRELMENVRQIYSEHNYSRLFI
XM_009993988 Chaetura pelagica                    VTQLKVFTQKSHSLLKRSQKSHPSGFNFHVLNHPGWLKVSPLTRYAPGSSPARSPPA-GAGPGPEPWQTSRPEPCRPEPSEK----PLPSG------AGAAPAEGG--ARMQE--GV-------------------EEDPAAEQSAGDPGEIVESVRQIYSEHNYCRLCT
XM_010133415 Buceros rhinoceros silvestris        VTQLKVFTQKSHSLLKRSPKSHPSGFNFHVLNHPGWLKVSPLTRYAPASSPARSPPA-GAGLAPGHSQTLKPVLCRPEPSEK--LLQPPSE------AGVAQEGGE--APTKE--VV-------------------EETPAAVQSTGDPRELTESGRQIYSEHNYCRLPI
XM_010178604 Mesitornis unicolor                  VTQLKVFTQKSHSLLKRSQKSHPSGFNFHVLNHPGWLRVSPLTRYAPGSSPTRSPPA-GAGPGPGHSQTSKPALCKPEPSEK--LPQPPSE------AGAARAEGE--ALTKE--EA-------------------EENPAAAQSTGDQGELTESVRQIYNEHNYCRLSV
XM_010084600 Pterocles gutturalis                 VTQLKVFTQKSHSLLKRSQKSHPSGFNFHVLNHPGWLRVSPLTRYAPGSSPARSPPAPGAGPVPGPSQTLKPVLCRHEPSEK-LLLQPPSE------VGAARVEGE--ALTKE--AA-------------------EENPAAVPSTGDPRELTESVRQIYNEHNYCRLSI
XM_009562634 Cuculus canorus                      VTQLKVFTQKSHSLLKRSPKSHPSGFNFHVLNHPGWLRVSPLTRYAPGSSPTQSPPA-GAGLGPEHSQTLKPVLCRPEPSER--LQQPPLE------EGVAPVEGE--ALTKE--VA-------------------EENPAAVESTGDQRELVESVRQIYNEHNYCRLSI
XM_005499680 Columba livia                        VTQLKVFTQKSHSLLKRSQKSHPSGFNFHVLNHPGWLKVSPLTRFAPGSSPSAADPA-G--------------------------------------------------------------------------------------------LAESARQIYNEHNYCRLSI
XM_005011000 Anas platyrhynchos                   VTQLKVFTQKSHSLLKRSQKSHPSGFNFHVLNHPGWLKVSQLTKYAPGSSPTRSPPA-GAGAGPEHSQTLKPVLCRPEPSEK--LLQLPSE------VGVAQAEGE--ALTKE--VA-------------------EENPTATQSTGDQRELMESVRQIYNEHNYCRLSV
XM_013198496 Anser cygnoides domesticus           VTQLKVFTQKSHSLLKRSQKSHPSGFNFHVLNHPGWLKVSQLTKYAPGSSPTRSPPA-GAGAGPEHSQTLKPVLCRPEPSEK--LLQLPSE------VGAAQAEGE--ALTKE--VA-------------------EENPTATQSTGDQRELMESVRQIYNEHNYCRLSV
XM_010120851 Chlamydotis macqueenii               VTQLKVFTQKSHSLLKRSQKSHPSGFNFHVLNHPGWLKVSPLTRYAPGSSPARSPPA-GAGLAPEPSPILKPVLCKPEPSEK--LLQPPSE------AGVARAEGE--TLMKE--VV-------------------EENPGAIQSTGGQRELTESVRQIYNEHNYCRLSI
XM_009962456 Tyto alba                            VTQLKVFTQKSHSLLKRSQKSHPSGFNFHVLNHPGWLKVSPLTRYAPGSSPARSPPA-GAGLGPEHSQTLRPVLCKPEPSEK--LLQPPSE------GGVAGVEGE--ALTKE--VV-------------------EEQPAATQSTGDQRELTESVRQIYNEHNYCRLSI
XM_009933575 Opisthocomus hoazin                  VTQLKVFTQKSHSLLKRSQKSHPSGFNFHVLNHPGWLKVSPLTRYAPGSSPTRSPPA-GAGLGPTP--------------------RPPSA------VGVARAEGE--APTKE--VA-------------------EENPAATQGAGEQRELVESVRQIYNEHNYCRLSI
XM_013958321 Apteryx australis mantelli           VTQLKVFTQKSHSLLKRSQKSHPSGFNFHVLNHPGWLKVSQLTRYAPGSSPTRSPPA-GAGPGPEHLQTLKPVLCKPEPSEK--LLQPPLE------VGVAQAEGE--ALTKE--VV-------------------EENPVAIQSTGDQRELMESVRQIYNEHNYCRLSI
XM_014956841 Calidris pugnax                      VTQLKVFTQKSHSLLKRSQKSHPSGFNFHVLNHPGWLKVSPLTRYAPGSSPTRSPPA-GAGLGPEPSQTLKPVLCRPEPSEK--LRQPPSE------VGVARAEGE--ALMKE--VV-------------------EENPAAVQSTGDQRELMESVRQIYNEHNYCRLSI
XM_009471438 Nipponia nippon                      VTQLKVFTQKSHSLLKRSQKSHPSGFNFHVLNHPGWLKVSPLTRYAPGSSPTRSPPA-GAGPGPEHSQTLKPVLCKPEPSEK--LPQLPSE------VGVAQAEGE--ALMKE--VV-------------------EENPAAIQSTGDQRELMESVRQIYNEHNYCRLSI
XM_010308576 Balearica regulorum gibbericeps      VTQLKVFTQKSHSLLKRSQKSHPSGFNFHVLNHPGWLKVSPLTRYAPGSSPTRSPPA-GAGLGPEHSQTLKPVLCKPEPSEK--LLQPPLE------VGVARVEGE--TPMKE--VV-------------------EENPAAVQSTGDQRELMESARQIYNEHNYCRLSI
XM_009893390 Charadrius vociferus                 VTQLKVFTQKSHSLLKRSQKSHPSGFNFHVLNHPGWLKVSPLTRYAPGSSPTRSPPA-GAGLGPEHSQTLKPVLCKPEPSEK--LLQPPLE------VGVAQVEGE--APMRE--VV-------------------EENPAAVQSTGNQRELMESVRQIYNEHNYCRLSI
XM_009279537 Aptenodytes forsteri                 VTQLKVFTQKSHSLLKRSQKSHPSGFNFHVLNHPGWLKVSPLTRYAPGSSPTRSPPA-GAGLGPEHSQTLKPVLCKPEPSEK--LLQLPLE------VGVAQVEGE--APMRE--VA-------------------EENPAAMQSTGDQRELMESVRQIYNEHNYCRLSI
XM_009318746 Pygoscelis adeliae                   VTQLKVFTQKSHSLLKRSQKSHPSGFNFHVLNHPGWLKVSPLTRYAPGSSPTRSPPA-GAGLGPEHSQTLKPVLCKPEPSEK--LLQLSLE------VGVAQVEGE--APMKE--VA-------------------EENPAAMQSTGGQRELMESVRQIYNEHNYCRLSI
XM_009870040 Apaloderma vittatum                  VTQLKVFTQKSHSLLKRSQKSHPSGFNFHVLSHPGWLKVSPLTRYAPGSSPTRSPPA-GAALEP--------------------------------------------------------------------------------------ELMESVRQIYSEHNYCRLSI
XM_009511061 Phalacrocorax carbo                  VTQLKVFTQKSHSLLKRSQKSHPSGFNFHVLNHPGWLKVSPLTRYAPGSSPTRSPPA-GAGLEPEHSQTLKPVLCKPEPSEK--LLQLPLE------VGAAQVEGE--ALMKE--VA-------------------EENPAAIQSTGDQRELMESVRQIYNEHNYCRLSI
XM_011578340 Aquila chrysaetos canadensis         VTQLKVFTQKSHSLLKRSQKSHPSGFNFHVLNHPGWLKVSPLTRYAPGSSPTRSPPA-GAGLGPEHSQTLKPVLCKPEPSEK--LLQPPLE------VGVAQVEGE--VLMKE--VV-------------------EENPAAIQSTGDQRELMESVRQIYNEHNYCRLSM
XM_010569380 Haliaeetus leucocephalus             VTQLKVFTQKSHSLLKRSQKSHPSGFNFHVLNHPGWLKVSPLTRYAPGSSPTRSPPA-GAGLGPEHSQTLKPVLCKPEPSEK--LLQPPLE------VGVAQVEGE--VLMKE--VV-------------------EENPAAIQSTGDQRELMESVRQIYNEHNYCRLSM
XM_009914948 Haliaeetus albicilla                 VTQLKVFTQKSHSLLKRSQKSHPSGFNFHVLNHPGWLKVSPLTRYAPGSSPTRSPPA-GAGLGPEHSQTLKPVLCKPEPSEK--LLQPPLE------VGVAQVEGE--VLMKE--VV-------------------EENPAAIQSTGDQRELMESVRQIYNEHNYCRLSM
XM_010283841 Phaethon lepturus                    VTQLKVFTQKSHSLLKRSQKSHPSGFNFHVLNHPGWLKVSPLTRYAPGSSPTRSPPA-GAGLGPEHSQTLKPVLCKPEPSEK--LLQPPLE------VGVAQVEGQ--ALMKE--VV-------------------EENPAAIQSTGGQRELMESVRQIYNEHNYCRLSI
XM_005239907 Falco peregrinus                     VTQLKVFTQKSHSLLKRSQKSHPSGFNFHVLNHPGWLKVSPLTRYAPGSSPTRSPPA-GAGLGPEHSQTLRPVLCKPEPSEK--LLQPPLE------VGVAQVEGE--ALMKE--VV-------------------EENPAAIQSTGDQRELMESIRQIYSEHNYCRLSI
XM_005444837 Falco cherrug                        VTQLKVFTQKSHSLLKRSQKSHPSGFNFHVLNHPGWLKVSPLTRYAPGSSPTRSPPA-GAGLGPEHSQTLRPVLCKPEPSEK--LLQPPLE------VGVAQVEGE--ALMKE--VV-------------------EENPAAIQSTGDQRELMESVRQIYSEHNYCRLSI
XM_010162275 Caprimulgus carolinensis             VTQLKVFTQKSHSLLKRSQKSHPSGFNFHVLNHPGWLKVSPLTRYAPGSSPTRSPPA-GAGPGPEHSQTLKPVLCKPEPSEK--LLQLPLE------VGVAQVEGE--APTKE--VV-------------------EENPAAVQSTGDQGELMESVRQIYNEHNYCRLSI
XM_009694853 Cariama cristata                     VTQLKVFTQKSHSLLKRSQKSHPSGFNFHVLNHPGWLKVSPLTRYAPGSSPTRSPPA-GAGPGPEHSQTLKPVLCKPEPSEK--LLQLPLE------VGVARVEGE--ALMKE--VV-------------------EESPAAVQSTGDQRELTESVRQIYNEHNYCRLSI
XM_009640313 Egretta garzetta                     VTQLKVFTQKSHSLLKRSQKSHPSGFNFHVLNHPGWLRVSPLTRYAPGSSPTRSPPA-GAGLGPEHLQTLKPVLCKPEPSEK--LLQLPLE------VGVAQVEGE--ALMKE--VV-------------------EENPAAVQSTGDQRELMESVRQIYNEHNYCRLSI
XM_009482169 Pelecanus crispus                    VTQLKVFTQKSHSLLKRSQKSHPSGFNFHVLNHPGWLKVSPLTRYAPGSSPTRSPPA-GAGLGPEHSQTLKPVLCKPEPSEK--LLQLPLE------AGVAQVEGE--ALMKE--VV-------------------EENPAAVQSTGDQRELMESVRQIYNEHNYCRLSI
XM_015426170 Gekko japonicus                      VTQLKVFTQKSHSPPKRNPKSHPSGFNFPVSNPPGWLKVSQPTRYVLGLSPIQSPPA-RTETGPRPLLTLRPVLCQAGPSERLLLLPPPLE------VGVGRGEGEAAARMKA--AA-----VVVVAAAAAAPAAEAGNPVAAQSAGGPRELTGSVCQIYSEHSYCRFPT
XM_008120859 Anolis carolinensis                  VTQLKVFTQKSHSPPKRNPKSHPSGFNFPVSNHPGWLKVSQPTRYVLGLSPIQSPPA-RTEVGPQPLLTLRPVLCKAVSSERLLLLPPPLPPPLPLGAGVGR-EGASGAQTKA--GAAGATVVVVTTAAAVAAGGGSGRPAAALGAGGRRELVGSVCPIYSEHSYCRLPS
```

amphibians

```
HADQ01029003 Bombina bombina                VTQFRVVTQKSHDLLKRNPKSLQYGSNFQGSNLPGWIKVCQLTRSVPGSSQVLIPLGGGLLATNLRTVTTVAIT----PDNRLGEGSSGAI-KGGAGLLTVS---EADPQSELLSSRLQIATEHNYCRPLL----
XM_018234430 Xenopus laevis                 VTQFKTFTQKSHSPPKKNPKSLQFGSNFLGSSLPGFIKVCQLTSSIPGSSQTLIPLGAGLLPAHLLTVKPVQLG------EEVVREEAESI-RQEAKPESEK---SENPQRENLESHLQVCAEHNYCRQQPRGTV
XM_012952772 Xenopus tropicalis             VTQFRVFTQKSHSPPKKNPKSHQFGSNFLGSSLPGFIKVCQLSSPIPGSSQTLIPLGAGLLPAHLLTVRPVQLG------EEGAREEAEPL-RQERNPGSER---SDNPQRETPQSHVQVCSEHNYCRPQP----
GEGL01031110 Megophrys                      VTQFRVFTQKSHSPPKKSQKSHQSGSSFLGSNLPGWIKVCQLTRSAQGSSQTLIPLGAGLIPAHLLTVKPVVLN--PQIQEVQAQGEAGVISKGEPGQEPPR---SENPQRNVREDSIQIAAEHNYCRPPL----
GEGK01045837 Leptobrachium boringii         VPQFRVFTQKSHSPPKKSQKSHQSGSSFLGSNLPGWIKVCQVTRSAQGSSQTLIPLGAGLIPAHLLTVKPVVLN--PPLQEVVAQGEAGVVGKGEPGQETSR---PEHPQRDVRADSIQIAAEHNYSRPPL----
XM_018556374 Nanorana parkeri               VTQLQVFTQKSHSPPKKNQKSPQSGSSFLASNLPGWLKVCQLTRSVPGSSQTLIPLGANLLPAHLLTVRPVAMTSRLPSVVAGVRGEVTATGRRAAGPGTPK---GGDQQREAMERSQQIAAEHNYCRPL-----
GEGH01003348 Polypedates megacephalus       VTQLQVFTQKSHSPPKRSQKSPQSGSSFLASNLPGWLKVCQLTRSVPGSSQTLIPLGASLLPAHLLTVKPVAMTSRPPLVEVVAREEGAPQGREEAGAGTPK---GEDQLSEDIERSQQIAAEHNYCRPLQGR--
GEGG01008506 Rhacophorus dennysi            VTQLQVFTQKSHSPPKRNQKSPQSGSSFHASNLPGWLKVCQLTRSVPGSSQTLIPLGANLLPAHLLTVKPVAMTSRPPLVEVVAREEATPPGGDEAGTGTPK---G-DQLREDTERSQQIAAEHNYCRPLQGR--
GECV01019509 Microhyla fissipes             VTQLHVFTQKSHSPPKKSQKSLPSGSSFLASNLPGWLKVCQLTRSVPGSSQTLIPLGANLLPAPLLTVRPVAVTSRPPLVAVVAREGATAPETEGTGPGTPNGGGGGGRLSEDIERSQQIAAEHNYCRPLQ----
GDDO01068111 Rana catesbeiana               VTQLQVFTQKSRSPPKKNQKSHQSGSSFLASNLPGWLKVCQLTRSVPGSSQTLIPLGANLLPAHLLTVRPVAMTSRPPLVAAAAREEATAPG----------------LSTDIARRQQIAAEHNYCRPLQ----
```

other

```
XM_007885779 Callorhinchus milii            GPQLIAFGQKGNNQRERSQRCHLLGSSSQESSLRGSSRVSQRTR--SGQGSSARPGQDQAVGEEAAGRGRGPGHWPTSRPEPSKPEPSGKLRLLRPPA---------GEGWRGREKVLVVM-----GEVPLPI-PTEEQRLLNRVWHNISLEHSYAKPVPRAAQRSPGLAAALIVSKVL
XM_014498298 Latimeria chalumnae            VTQLRVFTQKSHSLLKKSPKCHLSGFNFQGSNHAGWLKGSQPTRYVPGLSLAQSP---------QAEPGLVPGHLQISKPVPCRPEHRERPLLPQPPLLLVEGGGLEGGKG-EGAEVVVVKIVEEEEEEKKDKMSAMENQRELHR-RNQILIEHNYCRAVEQ-----------VRAGPVL
GEUG01028913 Amia calva                     VTQLTVFTQKSHSPPKKSPKSHLSGFNSPGSNHPGWLKAHQHTRYVPALSLTTKL---------PGGAGLAPGLWLTSKPVPSRPGPNERQLLLLQPLGKVP------GLG-EAREVGVIRI--GEGESTRVPTSPGEARGRLQNLRRLISQEHNYCRPL-------------------
XM_015365106 Lepisosteus oculatus           VTQLTVFTQKNHSPPKKSPKSLLSGFNSPGSNLPGWLKAHQPTRYVPALSLTARL---------PAGAGLGPGLWLTSKPVPSRPGPSERPLLLLQPLVKAQ------GPG-EAQEEGVA-------ESTRSLLSQEAAREHLQSLRHNITQEHNYCRPPLR-----------------
```

teleosts

```
XM_011473016 Oryzias latipes                VPQLTVSVQKSRSQQPKNRRCHPFGFNYPESNLHGSKGTPLTRSAHGLCPPERAHAALELGEHELWQTSKPEPSKPEPRGRPLLLLRPLAKGQGRPVSG--CGLLLGYRIATVNDEEESTQDQPSLEEGGRKEPE-----------MRRRRPVKWRSRNRLQTLIRLEHNYSFNM
XM_019891036 Hippocampus comes              VPQLTGSIQRNRSRQRRNPRCLQSGFNCPESNRPGSKGTPPTRSVPASCPLERPRGGRGRGARGRSQTSKPARSKPVPSGKPLLLLQPLPTGQGRTGSG--CGLLLGYRIATVDREHKNIQGRLSQEEE-----------------VEEGEVVGHRSRERLVKRIHLEHSYSVPM
XM_017487517 Ictalurus punctatus            VTQLTVCTQKSRSLQQRNPRSRQSGFSSPGSNLRGSKGRQRTKSVPASCPPTRGRGAVGRG-RAPWRTSRPVPSKPVHSGKPLLLLQPLETGQGLGGAA--RGVVLGYRIAPAGGVRESTRVPLNLEEEEEEEEEEE---------------LMWRARNRLQALIRLEHNYSYPM
XM_007246841 Astyanax mexicanus             VTQLTVCTQKSRSRQPRSPRSRQSGFSSPGSNRPGSKG-QRTKSVPASCPPTRGRGAVGRG-RAPWRTSRPAPSKPVHSGKPLLLLQPLGTGQGLGGAA--RGVVLGYRIAPAGGVRESTRVPLNLEEEEEEEEDEEEE--------EEEEEELMWRNRNRLRALIRLEHNYSYPM
XM_017708670 Pygocentrus nattereri          VTQLTVCTQKSRCRQQRSPRSRQSGFSCPGSNRPGSKGHQRTKSVLASCPPTRGRGAVGRG-RAPWRTSRPVPSKPVHSGKPLLLLQPLGTGQGLGGAA--RGVVLGYRIAPAGGVRESTRVPLNLEEEEEEEEEEE----------DEAEQELMWRNRNRLRALIRLEHNYSYPI
XM_005162338 Danio rerio                    VTQLTVCTQKSHSLQQRSPRSRQSGYSSPGSNRPGSRGRQRTKSVPASSPPTKGRGALGRG-RAPWRTSKPVHSKPVPSGKPLLLLQPLGMGEGLGGAA--RGVVLGYRIAPAGGVQESTQVPLNLEEEEGDEEEE-----------------LIWRSRNRLRALIRREHNYSCLV
XM_016513134 Sinocyclocheilus rhinocerous   VTQLTVCTQKSHNLQQRSPRSRQSGYSSPGSNRPGSRGRQRTKSVPASSPPMKGHGALGQG-RAPWRTSRPVHSKPVPSGKPLLLLQPLGMGEGLGGAA--RGVVLGYRIAPAGGVRESTQVPLNQEEEEE----------------------LIWRSRNRLRALIRLEHNYSRPV
XM_016503785 Sinocyclocheilus anshuiensis   VTQLTVCTQKSHNLQQRSPRSRQSGYSSPGSNRPGSKGRQRTKSVPASSPPAKGHGALGQG-RAPWRTSRPVPSKPVPSGKPLLLLQPLGMGEGLGGAA--RGVVLGYRIAPAGGVRESTQVPLNLEEEEADEEEE-----------------LIWRSRNRLRALIRLEHNYSCPV
XM_016273902 Sinocyclocheilus grahami       VTQLTVCTQKSHNLQQRSPRSRQSGYSSPGSNHPGSKGRQRTKSVPASSPPAKGRGALGQG-RAPWRTSRPVPSKPVPSGKPLLLLQPLGMGEGLGGAA--RGVVLGYRIAPAGGVRESTQVPLNLEEEEADKEEE-----------------LIWRSRSRLRALIRLEHNYSCPV
XM_012820794 Clupea harengus                VTQLTVCTRKSRSPQQRNPRSHQSGYNSPGSNRPGSRGRQRTKSVPALCPPTRGRGAQGRG-RARSRTSKPGPSRPAPSERQLLLLQPLATGQGRAGAGAARGAVLAYRIAAVGGGPESTRARSSREEAAEEEA-------------------AMWRSRAHLRALIRLEHNYSCPL
XM_010875623 Esox lucius                    VPQSTGSTRRSPSRQLRNPRSHLSGFNSPESNRPGSKGRRPTRSAPASCHPVRARGARGRG-RAPWRTSKPAPSRPGPSARPLLLLQPLGMGQGPAGA----GLVLGYRIAAVEDEHESTQDQSSPEEEEEEHQDP-----------------EVWRSRDRLRALIRLEHNYSCPI
XM_014135258 Salmo salar                    VPQLTVFTRKSRSRQQRSPRSHLSGFNSPESNRPGSKGRQPTRSVPASCPPARARGALGRG-RAPWRTSKPAPSRPGPSARPLLLLQPLGTDQGRAGA----GLVLGYRIAAVEDELESTQDPSSPEEQE------------------------MWRSRDRLRALIRLEHNYSSPN
XM_014475275 Xiphophorus maculatus          VPQLTVSVQRSHCRQPKNPRCHLSGFNCPESNLPGSKGTPPTRSAPELCPRPNARGGRARVARGPLPTSRPALSKPVPNAR--LLLRPLARGQGRPVSG--CGLRLGYRMQQRRRT-ESIQDHRXXXXXXXGXXEE-----------EEEEEMVRLKSRNFLQNLIRLEHNYSFSM
XM_015396824 Cyprinodon variegatus          VPQLTACVQRSRSRQPKSPRCHLSGFNFPESNLPGSKVTPPTRSVPGSCPRRNARGGRGRAARGLWQTSRPAPSKPAPNGR--LLLRPLPRGRGRPVSG--CGLLLGYRIAAANEEAESTQDQSSPEEEEEEEMEE-----------EEEEEPMGVKSRTFLRSLIRLEHNYSFPM
XM_012877575 Fundulus heteroclitus          VPQLTVSVQRSRSRQPKNPRCHLSGFNFPESNLPGSKGTPPTRSVPALCPRPNARGGRGRAARGHWQTSRPAPSKPVPSAR--LLLQPLARGQGRPVSG--CGLLLGYRIAAANEEHESIQDQSSPEEAEEEEMEE--------------EEVTGLRSRNFLRKLIRLEHNYSFSM
XM_015004530 Poecilia mexicana              VPQLTVSVQRSHSRQPKNPRCHLSGFNFPESNLPGSKGTPPTRSAPESCPRPNARGGRVRVARGPSQTSRPALSKPVPNAR--LLLRPLARGQGRPVSG--CGLLLGYRIAAANEEQESIQDQSSQEEEEEEEEEEEE---------EEEEKRVGMKSRNFLQNLIRLEHNYSFSM
XM_007575434 Poecilia formosa               VPQLTVSVQRSHSRQPKNPRCHLSGFNFPESNLPGSKGTPPTRSAPESCPRPNARGGRVRVARGPSQTSRPALSKPVPNAR--LLLRPLARGQGRPVSG--CGLLLGYRIAAANEEQESIQDQSSQEEEEEEEEEEEE---------EEEEKRVGMKSRNFLQNLIRLEHNYSFSM
XM_008412961 Poecilia reticulata            VPQLTVSVQRSHSRQPKNPRCHLSGFNFPESNLPGSKGTPPTRSAPESCPRPNARGGRVRVARGPSQTSRPALSKPVPNAR--LLLRPLARGQGRPVSG--CGLLLGYRIAAANEEQESIQDQSSPEEEEEEEEEE------------EEEERVGMKSRNFLQNLIRLEHNYSFSM
XM_015048376 Poecilia latipinna             VPQLTVSVQRSHSRQPKNPRCHLSGFNFPESNLPGSKGTPPTRSAPESCPRPNARGGRVRVARGPSQTSRPALSKPVPNAR--LLLRPLARGQGRPVSG--CGLLLGYRIAAANEEQESIQDQSSPEEEEEEEEEE--------------EERVGMKSRNFLQNLIRLEHNYSFSM
XM_008318529 Cynoglossus semilaevis         VPQLTVSVQKSHSRQPRNPRCRLSGFNCPESNLPGSKATPPTRSVPGSCRPMRARGGRGRGVHAPWQTSKHVPSKPGPSAKPLLLLQPLPMGRGQRGSG--CGLVLGYRIAAVEEEHESIPDLLNPGEEEEEEEEEEEEEE------EEAVEIKGWMSRDRLRALIHLEHNYSFSM
XM_005465660 Oreochromis niloticus          VPQLTVSVQKSRSRQQKNPRCHLSGFNCPESNLPGSKGTPPTRSVPGSCRPAKARAGRGQGVRAPWRTSKPVHNKPVPSARPLLLLQPLPTGQARPGSG--CGLVLGYRIAAMEDDRESIQDLSSPEEEEVEKKKVKME--------MKQGEVVGWNSRDRLQALIRLEHNYSFLM
XM_006807161 Neolamprologus brichardi       VPQLTVCVQKSRSRQQKNPRCHLSGFNCPESNLPGSKGTPPTRSVPGSCRPAKARAGRGQGVRAPWRTSKPVHNKPVPSARPLLLLQPLPTGQARPGSG--CGLVLGYRIAAMEDDRESIQDLSSPEEEEEVEKKKVKME-------MKQGEVVGWKSRDRLQALIRLEHNYSFLM
XM_005752308 Pundamilia nyererei            VPQLTVCVQKSRSRQQKNPRCHLSGFNCPESNLPGSKGTPPTRSVPGSCRPAKARAGRGQGVRAPWRTSKPVHNKPVPSARPLLLLQPLPTGQARPGSG--CGLVLGYRIAAMEDDRESIQDLSSPEEEEEEVEKKKVKME------MKQGEVVGWKSRDRLQALIRLEHNYSFLM
XM_004570926 Maylandia zebra                VPQLTVCVQKSRSRQQKNPRCHLSGFNCPESNLPGSKGTPPTRSVPGSCRPAKARAGRGQGVRAPWRTSKPVHNKPVPSARPLLLLQPLPTGQARPGSG--CGLVLGYRIAAMEDDRESIQDLSSPEEEEEEVEKKKVKME------MKQGEVVGWKSRDRLQALIRLEHNYSFLM
XM_005948017 Haplochromis burtoni           VPQLTVCVQKSRSRQQKNPRCHLSGFNCPESNLPGSKGTPPTRSVPGSCRPAKARAGRGQGVRAHWRTSKPVHNKPVPSARPLLLLQPLPTGQARPGSG--CGLVLGYRIAAMEDDRESIQDLSSPEEEEEVEKKKVKME-------MKQGEVVGWKSRDRLQALIRLEHNYSFLM
XM_015967370 Nothobranchius furzeri         VPQLTVSVRKNHSRQPKNPRCHLSGFNYPESNLPGSKGTPPTKSVPGSCPPARARVGRGRGGRGLWQTSKPVPSKPVPSAKPLLLLQPLAKGQGRPVSG--CSLLLGYRIAAMDDEQESIQDLSSPEEEMEEEE-----------------VVRWRSRNHLQALIHLEHNYSFSV
XM_014031322 Austrofundulus limnaeus        VPQLTVSVRKSHSRQTKNPRCHLSGFNYPGSNHPGSKDTPPTKSALGLSPLARARGGRGRGARGRWQTSRPVPSKPVRSAKPLLLLRPLPRGQGRPVSG--CGLLLGYRIAAMDDEHESIQDLSSPEEEEEEEEEEE----------EMEEDVMTWRSRNHLQNLIRLEHNYSFSI
XM_017438828 Kryptolebias marmoratus        VPQLTVSVRKSHSRQSKNPRCHLSGFNYPGSNRPGSKGTPPTKSALGSCPLAKGPGGRGRGARGPWQTSRPVPSKPVRSAKPLLLLQPLARGQGRPVSG--CGLLLGYRIAAMDDERESIQDLSSPEEEEETEEDV----------------MRWRSRNHLQNLIRLEHNYSFLM
XM_011619777 Takifugu rubripes              VPQLTVSVQRSRSRQQKSPGCRRSGFNCPESNLPGSKDTPRIRSVPASCRRGRGRGGRGRGAPAHWRTSKPAPSKPAPSARPLLLLRPLETGHHRQGSG--CGLLLGYRIAAVDDEHKSIRDLSSPEEEEEXEEEE-----------EEEGEVVGWRSRDRLQTLIRLEHNYSVPM
XM_020089223 Paralichthys olivaceus         VPQLTVSVRRSRSRQQRNPRCRLSGSNCPESNLPGSKGTPPTRSAHGSCPPVRARGGRGQGAHAPWQTSKPVPNKPGPSARPLLLLQPLATGQGRPQSG--CGLLLGYRIAAMDEEHESTQDQSSPEEEGAEEEEE-----------VEAGEMVGRRSRNQLRALICLEHNYCFSV
XM_008282558 Stegastes partitus             VPQLTVSVQRSRSRQQKNPRCRLSGFNCPESNLHGSKGTPPTRSAQGSCRPMRARGGRGPAGRVPWRTSKPVPSKPGPSARPLLLLQPLATGQGRPASG--CGLLLGYRIAAMDDERESIRDLSSPEEEEEEEETEME---------MEEGEVVGWRSRDRLQALIRLEHNYSFSM
XM_010784192 Notothenia coriiceps           VPQLTVSVRRSRSRQPKSPRCHLSGFNSPESNPPGSKVTQPTRSAPASCPPARARGRRGRAVRGPLRTSKPAPSKPGPSGRPLLLLQPLAKGQGR-GSG--CGLLLGYRIAAMDDERESIQDLSSPEEEEEEEEEEEEEEEEEEVVVVEEGEVVGWRSRDRLQALIRQEHNYSFTV
XM_018701579 Lates calcarifer               VPQLTVSVRRSRSRQQKSPRCRLSGFNCPESNLPGSKGTPPTRSVPGSCPPARAHGGRGRGAHAPWQTSKPVPSKPGPSARPLLLLQPLATGQGRPGSG--CGLLLGYRIAAMDEDRESIQDLSSPEEEEEEEEEEEEEVE-------VEAGGMVGWRSRDRLQALIRLEHNYCFSV
XM_019259947 Larimichthys crocea            VPQLTVSVRRSRSRQPKSPRCRLSGFNCPESNLPGSKGTPPTRSAQGSCPPARARGGRGRGARAPWRTSKPAPSKPGPSARPLLLLQPLATGQGRQGSG--CGLLLGYRIAAMDDERESIQDLSSPEEEEEEEEEEEE----------VEEGEVVGWRSRNRLQALIRLEHNYSFTM
```

ASXL2

Predicted ASXL2 TF peptide sequences following a −2 PRF at the conserved RG_GUC_UCU sequence. The conserved EH[N/S]Y motif is highlighted in purple.

mammals

```
XM_007666728 Ornithorhynchus anatinus             LPGFVFQPPSPVPTEQEPEPWQTSKPKPRWPKH--REQ----RPPPQPPRPPPPRPPSGEPFPGPARAVEEKVPGAGEEEEGEEEEERHLKEGVQDQVNSVQLEIGGAHWNWQELEAGEVREGCFPVLRGPNPKLRPR-PWERHHLTVSAEHNYSRPPRCHPARQSGDPARASHREPPPGDRHRL-
XM_012539839 Sarcophilus harrisii               LPGFVFQPLSLVPTEKEPEPLQISKQKPSWPKP--REQ-----LPPPPLPQLQL-PLLGGLSQVLA---------LEGEEKE-----HQEKEGIQDRVEPVKLEKG-THWNWQELEAGEVRESFYPMVQRLNPKQRPR-SQARHHLTVSLEHNYSKPPLYHPDLQSVEHVQVAPPQPSLTHPPQL-
XM_001380127 Monodelphis domestica              LPGFVFQPLSLVPTEQEPEPLQTSKQKPSWPKP--REQ-----LLPLPLPPPQL-PLLGGPSQGLA---------QEGEEKE-----QQEEEGIQDQAEPVKLEKG-THWNWQELEAGEVRESFYPTVQRLNPKLRPR-PQGRHHLTVSLEHNYSKPPPYQPELQAVEHVQGAPPQPSLTHPHQL-
XM_004582643 Ochotona princeps                  LPGLDFPSPSLVPTEQGPEPWQTSKPKPSWSKH--RGQ-----LLLQPLPLPPP-PLLVGPSLDPALGADKS---REKEVKG-----KVQEEGVQAQTEPMKVDQA-PPWSWQELEAGEVRESFYPVLQRFSPSLWPR-PQARPSFLVALEHNYSKPPQCLQHLSPVEQDQVPPLQPTERD-----
XM_007516745 Erinaceus europaeus                LPGLVSQSPSLVLTEPEPEPLQTSRPKPSWSKH--SGQ-----LPPPPLPPLPP-PQLAEPFQDLAPEVDKA---QEMVEKA-----DLPEQGVPPQTESVKLERA-PHWNWQELEAGEVRESFYPVAQRLSPSLRPG-SQARHSLLVALEHNYSKPPQCLQPLPPVEHVQVPLHQPTLT------
XM_002691461 Bos taurus                         LPGLVFQSPSLVLTEQEPEPLQTSKQKPSLSKH--RGL-----PPPPPPQPLLP-PQLEGPFQDLAQAVDRV---QGRGVKG-----ELLEEGILTQTKSVSPARA-PHWNWQELEAGEVRESFYPLGQTLSPSLRPG-PQASHSLLVALEHNYSKPPQCLPHLPSVEHVQVSPPQPTFMHSRQL-
XM_019970597 Bos indicus                        LPGLVFQSPSLVLTEQEPEPLQXSKQKPSLSKH--RGL-----PPPPPPQPLLP-PQLEGPFQDLAQAVDRV---QGRGVKG-----ELLEEGILTQTKSVSPARA-PHWNWQELEAGEVRESFYPLGQTLSPSLRPG-PQASHSLLVALEHNYSKPPQCLPHLPSVEHVQVSPPQPTFMHSRQL-
XM_005895687 Bos mutus                          LPGLVFQSPSLVLTEQEPEPLQTSKQKPSLSKH--RGP-----PPPPPPPPLLP-PQLEGPFQDLAQAVDRV---QGRGVKG-----ELLEEGILTQTKSVSPARA-PHWNWQELEAGEVRESFYPLGQTLSSSLRPG-PQASHSLIVALEHNYSKPPQCLPHLPSVEHAQVSPPQPTFMHSRQL-
XM_006046228 Bubalus bubalis                    LPGLVFQSPSLVLTEQEPEPLQTSKQKPSLSKH--RGP-----LPPPPPQPLLP-PQLEGPFQDLAQAVDRV---QGRGVKG-----ELLEEGILTQTESVSPARA-PHWNWQELEAGEVRESFYPLGQTLSPSLRPG-PQASHSLIVALEHNYSKPPQCLPHLPSVEHAQVSPPQPTFMHSRQL-
XM_010840577 Bison bison bison                  LPGLVFQSPSLVLTEQEPEPLQTSKQKPSLSKH--RGP-----PPPPPPQPLLP-PQLEGPFQDLAQAVDRV---QGRGVKG-----ELLEEGILTQTKSVSPARA-PHWNWQELEAGEVRESFYPLGQTLSPSLRPG-PQASHSLIVALEHNYSKPPQCLPHLPLVEHAQVSPPQPTFMHSRQL-
XM_004005731 Ovis aries                         LPGLVFQSPSLVLTEQEPEPLQTSKQKPSLSKHTAXGQ----------------PQLEGPFQDLAQAVDRV---QGRGVKG-----ELLEEGILTQTESVSLARA-PHWNWQELEAGEVRESFYPLGQTLSPSLRRG-PQASHSLIVALEHNYSKPPQCLPHLPSVEHVPVSPHQPTFMHSRQL-
XM_012158262 Ovis aries musimon                 LPGLVFQSPSLVLTEQEPEPLQTSKQKPSLSKH--RGQ-----LPPPQLQPLLP-PQLEGPFQDLAQAVDRV---QGRGVKG-----ELLEEGILTQTESVSLARA-PHWNWQELEAGEVRESFYPLGQTLSPSLRPG-PQASHSLIVALEHNYSKPPQCLPHLPSVEHVPVSPHQPTFMHSRQL-
XM_005686939 Capra hircus                       LPGLVFQSPSLVLTEQEPEPLQTSKQKPSLSKH--RGQ-----PPPLQPQPLLP-PQLEGPFQDLAQAVDRV---QGRGVKG-----ELLEEGILTQTESVSLARA-PHWNWQELEAGEVRESFYPLGQTLTPSLRPG-PQASHSLIVALEHNYSKPPQCLPHLPSVEHVPVSPHQPTFMHSRQL-
XM_005978907 Pantholops hodgsonii               LPGLVFQSPSLVLTEQEPEPLQTSKQKPSLSKH--RGQ-----PPPPQPQPLLP-PQLEGPFQDLAQAVDRV---QGRGVKG-----ELLEEGILTQTESVSLARA-PHWNWQELEAGEVRESFYPLGQTLSPSLRPG-PQASHSLIVALEHNYSKPPQCLPHLPSVEHLQVSPHQPAFMPSRQL-
NM_001270988 Mus musculus                       LPGLVFQSPSLVLTEQEPELWQTSKQKPSWSKH--KRQ-----QPQQLLQLLQP-PQLEGPFRDLALGVDKV---QERVVKG-----KLLEEEVQAQTQSVQMEKV-PHWNWPELEAGEVRENFYPVVL----NLRPI-CQAKHSLLVSLEHNYSKPPQCLRDLPAVEHAQVSHYQPT--------
XM_008764542 Rattus norvegicus                  LPGLVFQSPSLVLTEQEPELWQTSKQKPSWSKH--KRQ-----QPQQQLQQLQP-PQLEGPFQDLAPGVDEV---QERVVKG-----KPPEEGVQAQTQSVQMEKV-LHWNWPELEAGEVRENFYPVI----PNLRPI-HQAKHSLLVSLEHNYSKPPQCLQDLPPVEHAQVSHYQPT--------
XM_006981457 Peromyscus maniculatus bairdii     LPGLVFQSPSLVLTEQEPELWQTSKQKPSWSKH--RGQ-----QPLQPLQLLQP-PQLEGPFPDLGLGVDTA---QERVGKG-----KLLEEEVQAQTEPVQLERV-PHWSWQELEAGEVRESFYQAV----PNLRPM-HHAEHSLLVSLEHSYSKPPQCLQHLPTVEHAQASRYHPT--------
XM_005360858 Microtus ochrogaster               LPGLVFQSPSLVLTEQEPELLQTSKQKPSWSKH--RGQ-----QPQQPLQLLQP-PQLEGPFQDLAPGVDTV---QERVAKG-----KLVQEGVQAQTESAQLERV-PHWSWQELEAGEVCESFYPVV----PSLRPI-HQASPSLLVSLEHNYSKPPQCLQHIPSVEHAQASHYQPT--------
XM_005079112 Mesocricetus auratus               LPGLVFQSPSLVLTEREPELWQTSKQKPSWSKH--KGQ-----QLQQPLQLLQP-PQLEGPFQDLALGVDTV---QERVEKE-----KLIEEGVQAQREPVQLERV-SHWSWQDLEAGEVRESFYPVV----PNLRPI-HQAKHSLLVSLEHNYSKPPQCLQHLPSVEHAQASHSQPT--------
XM_003497015 Cricetulus griseus                 LPGLVFQSPSLVLTEQEPELWQTSKQKPSWSKH--KGQ-----QPQQPLQLLQP-PQLEGPSQDLALGVDTV---HKRVERE-----KLLEEGVHAQTEPVQLERV-PHWNWQELEAGEVRESFYPV----APNLRPI-HQAKHSLLVSLEHNYSKTPPCLQHLPSVEHAQTPHYQPT--------
XM_006732866 Leptonychotes weddellii            LPGLVFQSPSLVLTEQEPELLQTSKQKPSWSKH--RGR-----PPPQRPLLPPPRPQLEGPFQDLAPGADKA---PERAVTA-----KPLEEGGQTQTEAANLERA-PPWSWQELEAGEVRESFYPVGQRLSPSLRPR-AQSRHSLMVSLEHNYSKPPRCLQALPAVEPAQGPPPQPALTRPHQL-
XM_004394909 Odobenus rosmarus divergens        LPGLVFQSPSLVLTEQEPELLQTSKQKPSWSKH--RGR-----PPLQLPPPPPPRPQLEGPFQDLAPGADKA---QERAVTA-----KPLEEGGQAQTEAANLERP-PPWSWQELEAGEVRESFYPVGQRLSPSLRPR-AQTRHSLMVSLEHNYSKPPRCLQPLPAVEPVQGPPHQPTLTRPHQL-
XM_006880618 Elephantulus edwardii              LPGLVFQSPSLVLTEQEPEPLQTSKQKPSWSKH--KGQ-----LPPPLPPPLQP-PPLEGPFQDLVQGVDKV---QERPVTG-----KLLEEGARAQTKPLKPEEA-PPWNWQELEAGEVRESFYPVVQRLSPSLRPR-AQARLSLLVSLEHNYSKAPQCLPHLPSAEHAQVSRHHPTLTHPHQL-
XM_004696413 Echinops telfairi                  LPGLVFQSPSLVLTEQEPEPLQTSRQKPSWSKH--REQ-----PLQPPLPLLQP-PQLEGPFQVLGQEVDQVQG-QEKVVKG-----KLLGEGVQAPTEPVILERA-PHWNWQELEAGEVRESFYPVVQRLSPNLRPR-TQARPSLLVSLEHNYSKPPQCLPLRLSAEHAQVSPRQPTPSHPHQI-
XM_008834991 Nannospalax galili                 LPGLVFQSPSLVLTEQEPELSQTSKQKPSWSKH--RGQ-----QLQLQQPLLQP-PQLEGPFQDLALGVDKV---QERVMKG-----KLLEAGVQVQTESVQLERT-PHWNWQELEAGEVRESFYPVDQRLIPNLRPS-LQAKPSFLVCLEHNYSKPPQCLQHLPLLEDAQASHYQPTERNPTGKS
XM_003472847 Cavia porcellus                    LPGLVFQSPSLVLTEQEPELSQTSRQKRSWSKH--REP--QQPPPPPPPQLLQP-PQSEGPFPDQALGVDKV---QERVVKG-----RPLEEGVQAQTESVHLERA-LHWNWQELEAGEVRESFYPVVQTLNPNLRPR-AQARHSLIVFQEHNYSKPPQCLQHLPSVEHAQTPHHQPM--------
XM_004627442 Octodon degus                      LPGLVFQSPSLVLAEQEPGLLQTSKQKRSWSKH--REP-----QPPLLPQPLQP-PLLEGPFLDQALGVDKV---LERVVKG-----KPLEEEVQAQTESVQLERS-LHWNWQELEAGEVRESFYPAVQTLTPNLRSR-PQARHSLIVSLEHNYSKPPQCLQHLPSVEHAQTPHHQPM--------
XM_005400412 Chinchilla lanigera                LPGLVFQSPSLVLTEQEPELSQTSKQKRSWSKH--REL-----QLPLQPQPLQP-PQLEGPFLDQALGVDKV---QERVVKG-----KPLEEGVQAQTESVQLERA-LHWNWQELEAGEVRESFYPAVQTLNPSPRPR-PQARHSLIVSLEHNYSKPPQCLQHLPSVEHAQTPHHQPM--------
XM_004839122 Heterocephalus glaber              LPGLVFQSPSLVLTEQEPELLQTSKQKRSWSKH--REL-----QLQPLPQPLQP-PQLEGPFPAQAPGVDKV---PERVVKG-----KPPEEGVQPQTESVQLERA-LHWNWQELEAGEVRESFYPAVQTLNPNPRPR-PQARHSLIVSLEHNYSKPPQCLQHLPSVEHAQTLHHQPM--------
XM_010604841 Fukomys damarensis                 LPGLVFQSPSLVLTEPEPELLQTSKQKRSWSKH--REL-----QPLPQPQPQQL-PQLEGPFPAQALGVDKV---QERVVRG-----KPLEEGVQAQTESVQLERA-LHWNWQELEAGEVRESFYPAVQTLNPNPRPR-PQARLSLIVSLEHNYSKPPQCLQHLPSVEHAQTLHHQPM--------
XM_012727612 Condylura cristata                 LPGLVFQSPSLVLTEPEPEPLQTSKQKPSWSKH--RGQ-----QRLLQPPPLHM-PQLEGPFQDLVPGVDKV---QETVVKE-----KLLEEGAQTQTESVKLERA-PHWNWQELEAGEVRESFYPVVQRLSPSLRPR-PHARHSLLVSLEHNYSKPPQCLQHLPSLEHAQISHHQPTLMHPQL--
XM_004663740 Jaculus jaculus                    LPGLVFQSPSLVLTEQEPELLQTSKQKPSWSKP--RGQ-----QLQPLLQRLQP-PQLEGPFQDLALEVDTV---QEQAVNG-----KLLEEGVQVQTESVQLERT-PHWNWQELEAGEVRESIYPVVQRLIPNLRPR--QVRHSLLVSLEHNYSKPPQGLQHLP-MEHAQRSHHQPT--------
XM_004745948 Mustela putorius furo              LPGLVFQSPSLVLTEQEPELSQTSKQKPSWSKH--RGL-----PPP---------PPLEGPFRDLAPGADKV---QEREVTG-----KLLEEGVQTQTEAANLERA-PHWNWQELEAGEVRESFYPVVQSLSPSLRPR-AQTRHSLLVALEHNYSKPPQGLQQLPVEERAQASPLQPALTPPQQL-
XM_008254661 Oryctolagus cuniculus              LPGLGFQSPSSVLTEQEPEPSQTSKQKPSWSKH--RGQ-----PLPLPPPPLPP-PPLEGPFQDLAQGVDKV---QEKVAKG-----KLQEEAVQAQTGSVELERA-PHWNWQELEAGEVRESFYPVVQRLSPSLRPR-PQARHSLLVALEHNYSKPPQCLQHLPSAEGAQVSHQQPT--------
XM_013013735 Dipodomys ordii                    LPGLAFQSPSLVLTEQEPELLQTSKQKPSWSKH--RGQ-----QLQPLPQLLQP-PQLEGPFQDLALGVDKV---QERAVRG-----QLPEEGVRAQEESVQLERA-PHWNWQELEAGEVRESFYPVVQRFIPNLRPS-PQAWPSLLVSQEHNYSKPPQGLQYLPTVEQAQVSHQQPT--------
XM_019808476 Ailuropoda melanoleuca             LPGLVFQSPSLVLTEQEPELLQTSKQKPSWSKH--RGXXXXXXXXXXPPPPPPP-PQLEGPFQDLAPGVDKV---QERVVTG-----KRLEEGVQTQTEAANLERA-PHWNWQELEAGEVRESFYPVVQRLSPSLRPR-PQTRHSLVVSLEHNYSKPPQCLPHLPAAEHAQASHHQPTLTHPHQL-
XM_008700579 Ursus maritimus                    LPGLVFQSPSLVLTEQEPELLQTSKQKPSWSKH--RGQ----------PXPLLP-PQLEGPFQDLAPGVDKV---QERVVTG-----KRLEEGVQAQTQAANLERT-PHWNWQELEAGEVRESFYPVVQRLSPSLRPR-PQTRHSLIVSLEHNYSRPPQCLPHLPAAEHAQVSHHQPTLTHPHQL-
XM_019643761 Hipposideros armiger               LPGLVFQSPSLVLTEQEPELLQTSKQKPSWSKH--RGQ-----QPQLLPQPLQP-PPLEGPFQDLAQEVDKV---QERVVTG-----TLLEEGVQTHTESVKLERA-PHWNWQELEAGEVRESFYPVVQRLNPNLRPR-PRARPSLIVSLEHNYSKPPQGLQPLAAVEQASVSHQQLTLTHPHQL-
XM_006835227 Chrysochloris asiatica             LPGLVFQSPSLVLTEPEPEPLQTSKQKPNWSKH--RGQ-----LLQLPPPLLQL-PQLEGPFQDLAQGVDKV---PERVVRG-----KLLGEGVQAQTEPMKLERGPPHWNWQELEAGEVRENFYPVVQRLSPNPRPR-TLARHSLLVSLEHNYSKPPQCLLHLPPVEPAQVSHHQPTLTHPQQL-
XM_003432160 Canis lupus familiaris             LPGLVFQSPSLVLTEQEPELLQTSKQKPSWSKH--RGQ----QQPPPPLPPPQP-PPLEGPFQDLAQGVDKV---QERVVTG-----KLLGDVQTQTEVTHLERA-PHWNWQELEAGEVRESFYPVVQRLSPSLRPR-PQTRHSLLVSLEHNYSKPPQCLQPLPSVEHAQVSHQQPTLMHPHQL-
XM_017666555 Manis javanica                     LPGLVFQSPSLVLTEQEPELLQTSKQKPSWSKP--RGQ-----QLRLQPRPPQP-PQLEGPFQDLAQGVDKV---QERVGKG-----ELLKEGVQTRTEPVKLERA-PHWNWQELEAGEVRESFYPVVQRLSPNLRPR-PQTRHSLIVSLEHNYSKPPQCLQHLPSVDPAQMSHHQPTLTHPHQL-
XM_019712755 Rhinolophus sinicus                LPGLVFQSPSLVLTEQEPELLQTSKQKPSWSKH--RGQ-----QLPLQPQLLQP-PPLEGPFQDLAPGVDKV---QERVVKG-----TLLEGGTQTRTESVKLERA-PHWNWQDLEAGEVRESFYPVGQRLSPNLRAR-PQARHSLAVSLEHNYSKPPQCLQPLPSVEQAQVSHHQLTLTHSHQL-
XM_015064116 Acinonyx jubatus                   LPGLVFQSPSLVLTEQEPELLQTSKQKPSWSKH--RGQ-----QPPPPPPLPPP-PR--APFQDLARGVDKV---QERVVKG-----SLLEEGVQTQTEPVNPERA-PHWNWQELEAGEVRESFYPVFQRLSPSLRPR-PQTRLSLIVSLEHNYSKPPQCLQHLPSVEHAQVSHHLXVVKN-----
XM_019450044 Panthera pardus                    LPGLVFQSPSLVLTEQEPELLQTSKQKPSWSKH--RGQ-----QPPPPPPLPPP-PRLEGPFQDLARGVDKV---QERVVKG-----SLLEEGVQTQTEPVNPERA-PHWNWQELEAGEVRESFYPVFQRLSPSLRPR-PQTRLSLIVSLEHNYSKPPQCLQHLPSVEHAQVSHHQPTLTHPHQL-
XM_006930426 Felis catus                        LPGLVFQSPSLVLTEQEPELLQTSKQKPSWSKH--RGQ-----PPPPPPPLPPP-PRLEGPFQDLARGVDKV---QERVVKG-----SLLEEGVQTQTEPVNPERA-PHWNWQELEAGEVRESFYPVFQRLSPSLRPR-PQTRLSLIVSLEHNYSKPPQCLQHLPSVEHAQVSHHQPTLTHPHQL-
XM_010992973 Camelus dromedarius                LPGLVFQSPSLVLTEQEPELLQTSKQKPSWSKH--RGQ-----QPPPPPPPLQP-PQLEGPFRDLAQAGDKG---QERGVKG-----RLLEEGVQTQTESVKLERA-PHWNWQELEAGEVRESFYPVVQRLSPSLRPR-PQARHSLTVSLEHNYSKPPQRLQHPPSVEHAQVSGHQPTLAHPHQL-
XM_010948539 Camelus bactrianus                 LPGLVFQSPSLVLTEQEPELLQTSKQKPSWSKH--RGQ-----QPPPPPPPLQP-PQLEGPFRDLAQAGDKG---QERGVKG-----RLLEEGVQTQTESVKLERA-PHWNWQELEAGEVRESFYPVVQRLSPSLRPR-PQARHSLTVSLEHNYSKPPQRLQHPPSVEHAQVSGHQPTLAHPHQL-
XM_006190213 Camelus ferus                      LPGLVFQSPSLVLTEQEPELLQTSKQKPSWSKH--RGQ-----QPPPPPPPLQP-PQLEGPFRDLAQAGDKG---QERGVKG-----RLLEEGVQTQTESVKLERA-PHWNWQELEAGEVRESFYPVVQRLSPSLRPR-PQARHSLTVSLEHNYSKPPQRLQHPPSVEHAQVSGHQPTLAHPHQL-
XM_006197176 Vicugna pacos                      LPGLVFQSPSLVLTEQEPELLQTSKQKPSWSKH--RGQ-----QPPPPPPLLQP-PQLEGPFRDLAQAGDKG---QERGVKG-----RLLEEGVQTQTESVKLERA-PHWNWQELEAGEVRESFYPVVQRLSPSLRPR-PQARHSLTVSLEHNYSKPPQRLQHPPSVEHAQVSRHQPTLAHPHQL-
XM_004377581 Trichechus manatus latirostris     LPGLVFQSPSLVLTEQEPEPLQTSKQKPNWSKH--RGQ-----LLQPQPQPLQL-PQLEGPFQDLAQGVDKV---QERVVKG-----KLLGEGVQAQTESVKLERA-PHWNWQELEAGEVRESFYPVVQRLSPNLRPR-TQARHSLIVSLEHNYSKPPQCLPHLPSAEHAQVSHHQPTLTHPRQL-
XM_003411920 Loxodonta africana                 LPGLVFQSPSLVLTEQEPEPLQTSKQKPNWSKH--RGQ-----LLQPQLQPLQP-PQLEGPFQDLAQGVDKD---QERAVKG-----KLLGEGVQAQTESVKLERA-PHWNWQELEAGEVRESFYPVVQRLSPNLRPR-TQARHSLIVSLEHNYSKPPQCLPHLPSVEHAQVSRHQPVLTHPHQL-
XM_013996276 Sus scrofa                         LPGLVFQSPSLVLTEQEPELLQTSKQKPSWSKH--RGQ-----QLLLQPQLLQP-PQLEGPFQDLAQGVDRA---QERGVRG-----KLLEERVQTQTESVKLERA-PHWNWQELEAGEIRESFYPVVQRLSPNLRAR-PQARHSLIVSLEHNYSKPPQWLQHLPSVEPAQESHHQPTLTHPHQL-
XM_008063472 Carlito syrichta                   LPGLVFQSPSLVLTEQEPELLQTSKQKPSWSKH--RGQ-----QPLPLPQLLQP-PQLEGPFQDLAQGVDKV---QGRVVKG-----RLPEEAVQAQTKSVKLESA-SHWNWQELEAGEVRESFYPVVQRLSPNLRPGPPPA--SLIVSLEHNYSKPPQCLQHLLSVEHAQVSHHQPT--------
XM_015479905 Marmota marmota marmota            LPGLVFQSPSLVLTEQEPELLQTSKQKPSWSKH--RGQ-----QLQLPQPPLQP-PQLEGPFQDLALGVDKV---QERVVKE-----KLLEEGIQAQMESVQLERA-PHWNWQELEAGEVRESFYPVVQRLSPNLRPR-PQARHSLIVSLEHNYSKPPQCLQHLPSVEHARVSHHQLT--------
XM_005322571 Ictidomys tridecemlineatus         LPGLVFQSPSLVLTEQEPELLQTSKQKPSWSKH--RGQ-----QLQLPQPPLQP-PQLEGPFQDLALGVDKV---QERVVKE-----KLLEEGVQAQMESVQLERA-PHWNWQELEAGEVRESFYPVVQRLSPNLRPR-PQARHSLIVSLEHNYSKPPQCLQHLPSVEHARVSHHQLT--------
XM_015566378 Myotis davidii                     LPGLVFQSPSLVLTEQEPELLPTSKQKPSWSKH--RGQ-----QPPLPPPLLQP-PQLEGPSQDLAPGVDKA---QERAVKG-----KPPEEGIQTQTESENLERA-PHWNWQELEAGEVRESFYPVVQRLSPNLRPR-PQASHSLLVSLEHNYSKPPQCLR-LPSVEHAHVSHH----------
XM_008162352 Eptesicus fuscus                   LPGLVFQSPSLVLTEQEPELLPTSKQKPSWSKH--RGQ-----QLPLPPQLLPP-PQLEGPFQDLAQGVDKA---QERVVKG-----KLLEEGIQTQTESEKLERA-PHWNWQELEAGEVRESFYPVVQRLGPNLRPT-PQASHSLLVFLEHNYSKPPQCLQHLPSVEHAQVSHH----------
XM_005874900 Myotis brandtii                    LPGLVFQSPSLVLTEQEPELLPTSKQKPSWSKH--RGQ-----QLPLPPQLLQP-PQLEGPFQDLAQGVDKA---QERVVKG-----KPLEEGIQTQTESEKLERA-PHWNWQELEAGEVRESFYPVVQRLSPNLRPR-PQASHSLLVSLEHNYSKPPQCLHHLPSVEHAQVSHH----------
XM_014446882 Myotis lucifugus                   LPGLVFQSPSLVLTEQEPELLPTSKQKPSWSKH--RGQ-----QLPLPPQLLQP-PQLEGPFQDLAQGVDKA---QERVVKG-----KPLEEGIRTQTESEKLERA-PHWNWQELEAGEVRESFYPVVQRLSPNLRPR-PQASHSLLVSLEHNYSKPPQCLQRLPSVEHAQVSHH----------
XM_006162196 Tupaia chinensis                   LPGLVFQSPSLVLTEQEPELLQTSKQRPSWSKH--RGQ-----QPQQQQLLQP-PQLEGPFQDLAQGVDRV---RERVVKG-----RLPEEGVQAQTESVKLERA-PHWNWQELEAGEVRESFYPVVPRLSPNLRPR-PQAQHSLIVSLEHNYSKPPQCLQHLPSVEHAQVSHHPPT--------
XM_007191266 Balaenoptera acutorostrata scammoni LPGLVFQSPSLVLTEQEPELSQTSKQKPSWSKH--RGQ-----QLLLQPQLLQP-PRLEGPFQDLAQGVDKD---QERGLKG-----ELLEEGVQTQTESVKLERA-PHWNWQELEAGEVRESFYPVVQRLSPNLRPR-PQARHSLTVSLEHNYSKPPQCLHHLPSVERAQVSRHQPTLTHPHQL-
XM_007108431 Physeter catodon                   LPGLVFQSPSLVLTEQEPELSQTSKQKPSWSKH--RGQ-----QLLLQPQLLQP-PQLEGPFQDLAQGVDKA---QERGLKG-----ELLEEGVQTQTESVKLERA-PHWNWQELEAGEVRESFYPVVQRLSPNLRPR-PQARHSLTVCLEHNYSKPPQCLHHLPSVERAQVSRHQPTLTHPHQL-
XM_007470793 Lipotes vexillifer                 LPGLVFQSPSLVLTEQEPELSQTSKQKPSWSKH--RGQ-----QLLLQPQLLQP-PQLEGPFQDLAQGVDKV---QERGLKG-----ELLEEGVQTQTESVKLERA-PHWNWQELEAGEVRESFYPVVQRLSPNLRPR-PQARHSLTVSLEHNYSKPPQCLHHLPSVERAQVSRHQPTLTHPHQL-
XM_004312104 Tursiops truncatus                 LPGLVFQSPSLVLTEQEPELSQTSKQKPSWSKH--RGQ-----QLLLQPQLLQP-PQLEGPFQDLAQGVDKV---QERGLKG-----ELLEEGVQTQTESVKLERA-PHWNWQELEAGEVRESFYPVVQRLSPNLRPR-PQARHSLTVSLEHNYSKPPQCLHHLPSVERAQVSRHQPTLTHPHQL-
XM_012532454 Orcinus orca                       LPGLVFQSPSLVLTEQEPELSQTSKQKPSWSKH--RGQ-----QLLLQPQLLQP-PQLEGPFQDLAQGVDKV---QERGLKG-----ELLEEGVQTQTESVKLERA-PHWNWQELEAGEVRESFYPVVQRLSPNLRPR-PQARHSLTVSLEHNYSKPPQCLHHLPSVERAQVSRHQPTLTHPHQL-
XM_016209133 Miniopterus natalensis             LPGLVFQSPSLVLTEQEPELLQTSKQKPSWSKH--RGQ-----QLPLQLQLLQP-PQLEGPFQDLAQGVDKV---QERVAKG-----RLLEEGVQTQVESEKLERA-PHWNWQELEAGEVRESFYPVVQRLSPNLRPR-PQARHSLIVSLEHNYSKSPQCLQHLPSVEHAQVSHHQLTLAHPHQL-
XM_004418269 Ceratotherium simum simum          LPGLVFQSPSLVLTEQEPELLQTSKQKPSWSKH--RGQ-----QLPLQPQLLQP-PQLEGPFQDLAQGVDKV---QERVVKG-----KLLEEGVQTQRESVKLERA-SHWNWQELEAGEVRESFYPVVQTLSPNLRPR-PQARHSLIVSLEHNYSKPPQCLQHLPLVEHAQVSPHQPTLTHPHQL-
XM_014856740 Equus asinus                       LPGLVFQSPSLVLTEQEPELLQTSKQKPSWSKH--RGQ-----QLPLQLPLLQP-PQLEGPFQDLAQGVDKV---QERVVKG-----KLLEEGVQTQKESVKLERA-PHWNWQELEAGEVRESFYPLVQTLSPNLRPR-PQARHSLIVSLEHNYSKPPQCLQYLPSVEHAQVSHHQPTLMHPHQL-
XM_008542771 Equus przewalskii                  LPGLVFQSPSLVLTEQEPELLQTSKQKPSWSKH--RGQ-----QLPLQLPLLQP-PQLEGPFQDLAQGVDKV---QERVVKG-----KLLEEGVQTQKESVKLERA-PHWNWQELEAGEVRESFYPLVQTLSPNLRPR-PQARHSLIVSLEHNYSKPPQCLQYLPSVEHAQVSHHQPTLMHPHQL-
XM_001918196 Equus caballus                     LPGLVFQSPSLVLTEQEPELLQTSKQKPSWSKH--RGQ-----QLPLQLPLLQP-PQLEGPFQDLAQGVDKV---QERVVKG-----KLLEEGVQTQKESVKLERA-PHWNWQELEAGEVRESFYPLVQTLSPNLRPR-PQARHSLIVSLEHNYSKPPQCLQYLPSVEHAQVSHHQPTLMHPHQL-
XM_016121738 Rousettus aegyptiacus              LPGLVFQSPSLVLTEQEPELLQTSKQKPSWSKH--RGQ-----QLPPQPQPLQP-PQLEGPFQDLAQGADKV---QERVVKG-----TPLEEVQTQTESVKLERA-PRWNWQELEAGEVRESFYPVVQRLNPNLRPR-PQTRHSLIVSLEHNYSKPPQCLQHLPSVEHAQVSHHQLTVTHPHQL-
XM_011356536 Pteropus vampyrus                  LPGLVFQSPSLVLTEQEPGLLQTSKQKPSWSKH--RGQ-----QLPPQLQPLQP-PQLEGPFQDLAQGVDKV---QERVVKG-----TPLEEEVQTQTESVKLERA-PHWNWQELEAGEVRESFYPVVQRLNPNLRPR-PQARHSLIVSLEHNYSKPPQCLQHLPSVEHAQVSHHQLTVTHPHQL-
XM_006910290 Pteropus alecto                    LPGLVFQSPSLVLTEQEPELLQTSKQKPSWSKH--RGQ-----QLPPQPQPLQP-PQLEGPFQDLAQGVDKV---QERVVKG-----TPLEEEVQTQTESVKLERA-PHWNWQELEAGEVRESFYPVVQRLNPNLRPR-PQARHSLIVSLEHNYSKPPQCLQHLPSVEHAQVSHHQLTVTHPHQL-
XM_003787447 Otolemur garnettii                 LPGLVFQSPSLVLTEQEPEPLQTSKQKPSWSKH--RGQ-----LLQPPPQLLQP-PQLEGPFQDLAQGVDKV---QERVVKG-----KLLEEAVQAQTESMELEKA-PHWNWQELEAGEVRESFYPVVQKLSPSLRPRPPQTRHSLIVSLEHNYSKPPQCLQHLPSVEHAQMSHHQPT--------
XM_012788149 Microcebus murinus                 LPGLVFQSPSLVLTEQAPELSQTSKQKPSWSKH--RGQ-----QLLQLPQLLQP-PQLEGPFQDLAQGVDKV---QERVVKG-----KLLEEAVQAQTEPVELEKA-PHWNWQELEAGEVRESFYPVVQRLSPNLRPRPPQTRHSLIVSLEHNYSKPPQCLQHLPSVEHAQMSHHQPT--------
XM_012652406 Propithecus coquereli              LPGLVFQSPSLVLTEQEPELSQTSKQKPSWSKH--RGQ-----QLLQPPQLLQP-PQLEGPFQDLAQGVDKV---QERVVKG-----KLLEEAVQAQTESVELEKA-PHWNWQELEAGEVRESFYPVVQRLSPSLRPRPPQTRRSLIVSLEHNYSKPPQCLQHLPSVEHAQMSHHQPT--------
XM_008577069 Galeopterus variegatus             LPGLVLQSPSLVLTEQEPELLQTSKQKPSWSKH--RGQ-----QLQPPPQLLQP-PQLEGPFQDLVQGVDKV---QERAVKG-----KLLEEGVQAQTESVKLERA-PHWNWQELEAGEVRESFYPVVQRFSPNLRPR-PQARHSLIVSLEHNYSKPPQGLQHLPSVEHAQVSHHQPT--------
```

XM_008981035	Callithrix jacchus	LPGLVFQSPSLVLTEQEPELLQTSKQKPNWSKH--RGQ-----QLQLPLQLPQL-PQLEGPFQDLAQGVDKV---QEKVVKG-----RLLEEAVQPQTESVKLERA-PHWNWQELEAGEVRESFYPVVQRLSPSLRPRPPQARHSLLVSLEHNYSKPPQCLQHLPSVEHAQVSHHQPT--------
XM_017527302	Cebus capucinus imitator	LPGLVFQSPSLVLTEQEPELLQTSKQKPNWSKH--RGQ-----QLLQPPQLLQP-PQLEGPFQDLAQGVDKV---QERVVKG-----RLLEEAVQPQTESVKLERA-PHWNWQELEAGEVRESFYPVVQRLSPSLRPRPPQARLSLIVSLEHNYSKPPQCLQHLPSVQHAQASHHQPT--------
XM_010345790	Saimiri boliviensis boliviensis	LPGLVFQSPSLVLTEQEPELLQTSKQKPNWSKH--RGQ-----QLQLPPQLLQP-PQLEGPFQDLGQGVDKV---QERMVKG-----RLLEEAVQPQTESVKPERA-PHWNWQELEAGEVRESFYPVVQRLSPSLRPRPPQARHSLIVSLEHNYSKPPQCLQHLPSVEHAQVSHHQPT--------
XM_012453268	Aotus nancymaae	LPGLVFQSPSLVLTEQEPELLQTSKQKPNWSKH--RGQ-----QLQPPPQLLQP-PQLEGPFQDLAQGVDKV---QERVVKG-----RLLEEAVQPQTESVKLERA-PHWNWQELEAGEVRESFYPVVQRLSPSLRPRPPQARHSLIVSLEHNYSKPPQGLQHLPSVEHAQVSHHQPT--------
XM_015111861	Macaca mulatta	LPGLVFQSPSLVLTEQEPELLQTSKQKLNWSKH--RGQ-----QLPLPPQLLQP-PQLEGPFQDLAQGVDRV---QERVVKG-----RLLEEAVQAQTESVKLERA-AHWNWQELEAGEVRESFYPVVQRLSPSLRPRPPQARHSLIVSLEHNYSKPPQCLQHLPSVEHAQVSHHQPT--------
XM_005576367	Macaca fascicularis	LPGLVFQSPSLVLTEQEPELLQTSKQKLNWSKH--RGQ-----QLPLPPQLLQP-PQLEGPFQDLAQGVDRV---QERVVKG-----RLLEEAVQAQTESVKLERA-AHWNWQELEAGEVRESFYPVVQRLSPSLRPRPPQARHSLIVSLEHNYSKPPQCLQHLPSVEHAQVSHHQPT--------
XM_011950426	Colobus angolensis palliatus	LPGLVFQSPSLVLTEQEPELLQTSKQKLNWSKH--RGQ-----QPPLPPQLLQP-PQLEGPFQDLAQGVDRV---QERVVKG-----RLLEEAVQAQTESVKLERA-PHWNWQELEAGEVRESFYPVVQRLSPSLRPRPPQARHSLIVSLEHNYSKPPQCLQHLPSVEHAQVSHHQPT--------
XM_010383018	Rhinopithecus roxellana	LPGLVFQSPSLVLTEQEPELLQTSKQKLNWSKH--RGQ-----QLPLPPQLLQP-PQLEGPFQDLAQGVDRV---QERVVKG-----RLLEEAVQAQTESVKLERA-PHWNWQELEAGEVRESFYPVVQRLSPSLRPRPPQARHSLIVSLEHNYSKPPQCLQHLPSVEHAQVSHHQPT--------
XM_017876853	Rhinopithecus bieti	LPGLVFQSPSLVLTEQEPELLQTSKQKLNWSKH--RGQ-----QLPLPPQLLQP-PQLEGPFQDLAQGVDRV---QERVVKG-----RLLEEAVQAQTESVKLERA-PHWNWQELEAGEVRESFYPVVQRLSPSLRPRPPQARHSLIVSLEHNYSKPPQCLQHLPSVEHAQVSHHQPT--------
XM_007971691	Chlorocebus sabaeus	LPGLVFQSPSLVLTEQEPELLQTSKQKLNWSKH--RGQ-----QLPLPPQLLQP-PQLEGPFQDLAQGVDRV---QERVVKG-----RLLEEAVQAQTESVKLERA-PHWNWQELEAGEVRESFYPVVQRLSPSLRPRPPQARHSLIVSLEHNYSKPPQCLQHLPSVEHAQVSHHQPT--------
XM_011972683	Mandrillus leucophaeus	LPGLVFQSPSLVLTEQEPELLQTSKQKLNWSKH--RGQ-----QLPLPPQLLQP-PQLEGPFQDLAQGVDRV---QERVVKG-----RLLEEAVQAQTESVKLERA-PHWNWQELEAGEVRESFYPVVQRLSPSLRPRPPQARHSLIVSLEHNYSKPPQCLQHLPSVEHAQVSHHQPT--------
XM_012044335	Cercocebus atys	LPGLVFQSPSLVLTEQEPELLQTSKQKLNWSKH--RGQ-----QLPLPPQLLQP-PQLEGPFQDLAQGVDRV---QERVVKG-----RLLEEAVQAQTESVKLERA-PHWNWQELEAGEVRESFYPVVQRLSPSLRPRPPQARHSLIVSLEHNYSKPPQCLQHLPSVEHAQVSHHQPT--------
XM_003908363	Papio anubis	LPGLVFQSPSLVLTEQEPELLQTSKQKLNWSKH--RGQ-----QLPLPPQLLQP-PQLEGPFQDLAQGVDRV---QERVVKG-----RLLEEAVQAQTESVKLERA-PHWNWQELEAGEVRESFYPVVQRLSPSLRPRPPQARHSLIVSLEHNYSKPPQCLQHLPSVEHAQVSHHQPT--------
XM_011738081	Macaca nemestrina	LPGLVFQSPSLVLTEQEPELLQTSKQKLNWSKH--RGQ-----QLPLPPQLLQP-PQLEGPFQDLAQGVDRV---QERVVKG-----RLLEEAVQAQTESVKLERA-PHWNWQELEAGEVRESFYPVVQRLSPSLRPRPPQARHSLIVSLEHNYSKPPQCLQHLPSVEHAQVSHHQPT--------
XM_012497462	Nomascus leucogenys	LPGLVFQSPSLVLTEQEPELLQTSKQKPNWSKH--RGQ-----QPPLPPQLLQP-PQLEGPFQDLAQGVDKV---QERVVKG-----RLLEEAVQAQTESVKLERA-PHWNWQELEAGEVRESFYPVVQRLSPSLRPRPPQDRHSLIVSLEHNYSKPPQCLQHLPSVEHAQVSHHQPT--------
NM_018263	Homo sapiens	LPGLVFQSPSLVLTEQEPELLQTSKQKPNWSKH--RGQ-----QLPLPPQLLQP-PQLEGPFQDLAQGVDKV---QERVVKG-----RLLEEAVQAQTESVKLERA-PHWNWQELEAGEVRESFYPVVQRLSPSLRPRPPQARHSLIVSLEHNYSKPPQCLQHLPSVEHAQVSHHQPT--------
XM_004028952	Gorilla gorilla gorilla	LPGLVFQSPSLVLTEQEPELLQTSKQKPNWSKH--RGQ-----QLPLPPQLLQP-PQLEGPFQDLAQGVDKV---QERVVKG-----RLLEEAVQAQTESVKLERA-PHWNWQELEAGEVRESFYPVVQRLSPSLRPRPPQARHSLIVSLEHNYSKPPQCLQHLPSVEHAQVSHHQPT--------
XM_016948189	Pan troglodytes	LPGLVFQSPSLVLTEQEPELLQTSKQKPNWSKH--RGQ-----QLPLPPQLLQP-PQLEGPFQDLAQGVDKV---QERVVKG-----RLLEEAVQAQTESVKLERA-PHWNWQELEAGEVRESFYPVVQRLSPSLRPRPPQARHSLIVSLEHNYSKPPQCLQHLPSVEHAQVSHHQPT--------
XM_003827052	Pan paniscus	LPGLVFQSPSLVLTEQEPELLQTSKQKPNWSKH--RGQ-----QLPLPPQLLQP-PQLEGPFQDLAQGVDKV---QERVVKG-----RLLEEAVQAQTESVKLERA-PHWNWQELEAGEVRESFYPVVQRLSPSLRPRPPQARHSLIVSLEHNYSKPPQCLQHLPSVEHAQVSHHQPT--------

sauropsids

XM_007422356	Python bivittatus	LPGPPSQS----SGEQGPELWPTSKPGLGWPGPSEQLLLPKQPQQPPLLLLLPPLWKPSRAQGQEGGKEMTGAVH----------PQGGLMELHWTWQALEAGQVQEGFCPLVQPLSPQQTPQLQNQQPQAVLLEHSYS--RLPQHNPELQAATQAWRLRQSHNQQ---------------
XM_017838512	Lepidothrix coronata	LPGHVSPSPSPVRAGQGPGPWPTSRREPSRPRLRG---WRARRRSRELLAILLEHNYS--QGPPPSPE-----HPAPAPQPRPRH---------------
XM_014524044	Alligator sinensis	LPGPAFQPVSRVPEERGPELWRTSKQKPSRLRPRGQLLLLQQQ--PLPPPPPPLLGELSQGLGQEAAVGLGAEET-RRVAQQQVEPLKLEFGDMHWNCQELEAGEVREGLFPMVQGSSPRWRHRPRTRRHLTVCLEHNYS--KLPQYSPELQPAIKAQTLHHQPYQR---------------
XM_019497675	Alligator mississippiensis	LPGPASQPVSRVPEERGPELWRTSKQKPSRLRPRGQLLLLQQQ--PPPPPPPPLLGELSQGLAQEAAVGLGAEET-RRVAQQQVEPLKLEFGDMHWNCQELEAGEVREGLFPMVQGSSPRWRHRPWTRRHLTVCLEHNYS--KLPQYSPELQPAIKAQTPHHQPYQR---------------
XM_007062817	Chelonia mydas	LPGLAFQPLSPVLEEQGPELWQTSKQKPSWPRPRGQLL--QPP--PLPP---PPMGEPSQGPAQEVVVAQAGEEERRRIVQQQVEPMKLELKAVHWNWQELEAGEVREGLFPIVQGPIPKRRPRPRSGHHLSVSLEHSYS--KRPQYNPELQPATEAKIHHHQPHQH---------------
XM_005284123	Chrysemys picta bellii	LPGLTFQPLSPVLEEQGPGLWQTSKQKPNWPRPRGQLLQQQQP--PLL----PPMGEPSQGLVQEVVVAQEGEEERRRVVQQQMEPMKLELKAVHWNWQELEAGEVREGLFPIVQGPIPKRRPRPQSGHHLSVSLEHNYS--RRPQYNPELQPATKAKIHHHQLHQH---------------
XM_015859722	Coturnix japonica	LPGYVSQPHSSVQPEQAPEPWRTSRPKPSRSGLRGQRPPQPPQ--PLL------RGEQFQG--RAPVGAQRAVEG--QATQRRAELVKLEHEETHWNWQELEAGEVREGFFHAAQGPIPQWR--PRSSQQPPVFLEHNYS--KLPRCNPEVQLAIQVPAAPPQWHQR---------------
XM_010708024	Meleagris gallopavo	LPGYVSQPHSSVQPEQGPEPWRTSRPKPSRSGLRGQRPQQPPP--PLL------RGEQCQG--RAPVGAQRAAGG--RVTQRRVEPAKLEREETHWNWQELEAGEVREGFFHAAQGPIPQWR--LRSSRQPPVFLEHNYS--KLPRCNPEVQLAIQAPTAPPQRYQRLSKSAESNRAPQTWL
NM_001031096	Gallus gallus	LPGYVSQPHSSVRPEQGPEPWRTSRPKPSRSGLRGQRPPQPPP--PLL------RGEQSQG--RVPVGAQRAVEG--RVTQRRVEPAKLEREETHWNWQELEAGEVREGFFHAAQGPIPQWR--PRSSQQPPVFLEHNYS--KLPYCNPEVQLAIQVPTAPPQRYRR---------------
XM_009563752	Cuculus canorus	LPGLASRSHSPVQEEQGPEPWRTSRPRPSKPRLRGQRPLLLLP--PLLLP--PQQEEPSQGPARVVVEAYRVEGE-ARVTQ---SPAELELEGMHWNWQELEAGEVREGFFPI-----PKWGARPQTGHQRTILLEHNYS--KHP-------------------------------------
XM_010142315	Buceros rhinoceros silvestris	LPGHASPSHLPVQEEQGPELWRTSRPKPSKPRLRGQQPLLPP---PLPP---PQQGGLSPGLGQVV---DRVVAE-RRVTRQQAEPTKLEFEGAHWNWQELEAGEVREGFFPMVQGPTATWRARPPPRHHLTVLLEHNYSTLKLPHCSPELRPAVPAPAPPHPRCRH---------------
XM_014961567	Calidris pugnax	LPGRVSPSHFPVREEQGPELWRTSRPKPSKPRLRGQRPPLPPP--PLPP---PQPGGPSRGRDRGAAGPPRAVEE-RRVRQRPSEPTKLELEEPHWNWQELEAGEVREGFFPLVRGHRPKWRPSPRGRHRLAVLLEHNYS--RIPRCRPQLQPAARAPA-PRQRCRH---------------
XM_010002204	Chaetura pelagica	LPGHVSQSHLPVQEEQGPELWQTSRQEPSKPRLRGQQXXXXXX--X------XXXXXXXQGPGRA--GVPRAAEE-RRVTRQQVEPTKLEFEEMHWNWQELEAGEVREGFFPVVQGPIAKWRPRPRTRRRLTALLEHNYS--KLPHCNPQLQPAIQAPPPPHRRYRH---------------
XM_009909487	Picoides pubescens	LPGYVSHSHLPVQEEPGPELWQTSRQKPSKPRLRGQQPPPLPP--PPLPL--PPMGELSQGLDQEVVGPRREVEE-RRVTQHQVEPTQLELEEMHWNWQELEAGEVREGFFPIVQGPTAKWRTRPQSRHHLTILLEHNYS--RLPHCSPELRPAIQAPAPPHPRHRH---------------
XM_009669652	Struthio camelus australis	LPGHVFQPQLPVLEERGPELWQTSRRKPRR-------------------------GELAQGRDQEVVGAHPAVEE-RRVTQQQVESVKLEFEEMHWNWQELEAGEVREGFFPIVQVPIPKWRPRPRTRHHLTVFLEHNYS--KLPHYNPELQPAIQAPTPHHQRYQH---------------
XM_013200279	Anser cygnoides domesticus	LPGHVSQPQLPVREEQGPELWQTSRQKPSKSGPRGQRPPPQPP--PPP-------------------------------------EPTKLELEEMHWNWQELEAGEVREGFFPIVQGPIPKWRPRPRTSHQLTISLEHNYS--KLPHCNPELQAAIRAPTAPHQRHQH---------------
XM_009943397	Opisthocomus hoazin	LPGHVSQSHLPVREEPGPELWPTSRRKPR---QQRAEPAKLEFEEMHWNWQELEAGEVREGFFPIAQGAIPKWRPRPRSRHHLTTLLEHNYS--KLPRCNPELQPAVQAAAPPRQRCRH---------------
XM_005146457	Melopsittacus undulatus	LPGHVSQSHLPVREEGEPELWQTSRQKPSKPRLRGQQPLPPPP--PLLLLLPPRQGGLSQGLDQAVVGAHRVVEE-RRVTQQQVEPPRLELEEMYWNWQELEAGEVREGFLPTVQGAVLTWRPRPQTRHPLTILLEHNYS--KLPPCSPELQPAIQAPAPPHQQQRP---------------
XM_014287174	Falco cherrug	LPGHASQSRSPVREEQGPEPWQTSRQKPSKPRLXX----------------------------XXXVGAHQPVEE-RRVTQPQAEPTKLEFEEMHWNWQELEAGEVREGFFPIVQGPVPKWRPRPRTRHHLTILLEHNYS--KLPHHSPELQPAIPAAAPPPQRYQH---------------
XM_009890372	Charadrius vociferus	LPGHVSPSHSPVREEQGPELWQTSRQKPT---EPTKLEFEETHWNWQELEAGEVREGFFPIVQGHVPKWRPRPRARHRLTILLEHNYS--KLPRCNPELHPATQAPAPPQQRCRH---------------
XM_009467461	Nipponia nippon	LPGHVSHSHSPVQEEQGPELWQTSRQKPSK----------------------------AQGLDRAAAGAHQAAEE-RRVTQQRAEPPKLEFEETHWNWQELEAGEVREGFFPIVQGPTPKWRPRPRTRHHLAVLLEHNYS--KLPHCNPEPQPAIQAPAPPPPR------------------
XM_009275117	Aptenodytes forsteri	LPGHVSQSHSPVREEQGPELWQTSRQKPSKPRLRGQR-LPPPP--PLP----PRRGGLSQGLDRAVVGAHRAAEE-RRVTQQQAEPTKLEFEEMHWNWQELEAGEVREGFFPIVQGPIPKWRPRPQTRHHLTILLEHNYS--KLPHCNPELQPAVQAPAPPPPRYRHWRKSTE---------
XM_011598858	Aquila chrysaetos canadensis	LPGHVSQSHLPVREEQGPELWQTSRQKPSKPRLRGQQPLPLPP--PLPLL--PRRGGLSQGLDRAVVGAHRAVEE-RRVTQQRVEPTKLEFEEVHWNWQELEAGEVREGFFPIVQGPIPKWRPTPRTRHHLTILLEHNYS--KLPHCSPELQPAIQAPAPPHQRYRH---------------
XM_010565910	Haliaeetus leucocephalus	LPGHVSQSHSPVREEQGPELWQTSRQKPSKPRLRGQQPLPPPP--PLLPL--PRRGGLSQGLDRAVVGAHRAVEE-RRVTQQRAEPTKLELEEVHWNWQELEAGEVREGFFPIVQGPIPKWRPRPRTRHHLTILLEHNYS--KLPHCSPELQPAIQAPAPPHQRYRH---------------

amphibians

GFBM010877296	Ambystoma mexicanum	LPGLLFQPPLPVLGEQEPEPWQISRQRLTWPEHNEQLLQLQQPQL-LQSEILSQVLAQ----EAEVQEE-----------GTIDVEVKPMKLEARLSPWNWEELEAGEVRGDLCNLIWTSNPVQKRRPRSHHQLTVGQEHNYSKPSMYNPELQPVPQVYLPHLTPETLLH------------
XM_018089999	Xenopus tropicalis	LPGPIFQPPAPVPAEQEQEPWQTSKPKPSWPEPRGQQRRQRQQQHQLASLSVHQCQGQGLEGEPDLEEEPDLEEEPDLEERRGIQQLAPVKQDTGPEYWTWEVLEAGEVRGEIQPFHHAPRLPGRPRTHCRYHLTVQQEHSYCKNPVYNPDTQ-----------------------------
XM_018264742	Xenopus laevis	LPGPIFRAPVPVPVEQEQGPWQTSKPKPSWLEPREQ---QQQQQHQPASPLLHQSQGQ------DLEEE---EEELGLGQERGIQQMAPVKQDTGLEYWTWDVLEAGEVRGEIQPIHQVARVPGRPRTQCRYHLTVQQEHSYCKNPVYNPETQ-----------------------------
XM_018574722	Nanorana parkeri	LTGSRIQPAATVQAGPEPARWPTSRPEPSWPEQRGQ-----QLQLPMAAPSPGRAPGE------EQRQE-------------AVREVKSMK--AGGGYWTWEVLEAGEVKGELQPLQQLAKTKAKSRLQGRHRLTVQQEHSYCKNPVYNPESQL-----LQDSSRPQNVTFPSTSKTKTYYL
GEGH01064887	Polypedates megacephalus	LTGSHIQPAETVQAEQEPVPLPTSRPKPSLPEQREQ-----QLQLPMEVPSLDQAREE------EQCQE-------------VVQEVKSMK--AQKEYWTWEVLEAGEVKGELQPLHQLAKIKPKSRLHRRYRLTVQQEHSYCKNPVYNPESQLLL---LQDSS------------------
GDDO01077260	Rana catesbeiana	LTGRRIQPPATVQAEQGPVLWPTSRLKPSWPEQREQ-----QPQLPMEVPSLDQALGE------EQCQE-----------------VTSMR--AGREYWTWEVLEAGEVKGELQPLNQLVKTKAKSRLRGRHRLTVQQEHSYCKNPVYNPESQLLLPLEAQDSSRPQSVTLPPPSKIKTHSL

other

XM_015348384	Lepisosteus oculatus	LPGPHSQPPFPVLGAQGLAPWQTSRRRPNWPGPSGRRQQRL--LQRPPRDL--PRGVQCPDRGLGEGAGHRGEPATLGLQSERRSLPEQNWEELEEGELREGFYL------PVQTPRSRSLPVPSVILEHNYSRLPRHRPEPAVLLQPSVHYLQTLHRPPHLQ
XM_007900450	Callorhinchus milii	LQGPLSHRSSLVQGELEPGLSQTSKQKPSLPKLS---------VQQPPQLQ--VELCQAPVR-------------EEGVKSDDRAV-QWKWTELEAGEVLEGPCLIVQDNGPNHWKRPRSSQQPIWIQEHNYSKPPWRNLCLQPTLQAPTDAQLQLQQAQHQH
XM_006005057	Latimeria chalumnae	LPGPLFQLQSPVQGERGPEPWQISKPKPSSQRLRGRQQQPQPPLPLPPQRQGGPSQARVQEVGVQKEEGVVVVPQTELVKMEEMGA-HWNWSELEAGEVREGLFPAKLKPTPERRPRARPCHRLTIALEHNYSQPPTCQPRTQVKTQHP-------KLPHHQQ
